# Deoxynybomycins inhibit mutant DNA gyrase and rescue mice infected with fluoroquinolone-resistant bacteria

**DOI:** 10.1038/ncomms7947

**Published:** 2015-04-24

**Authors:** Elizabeth I. Parkinson, Joseph S. Bair, Bradley A. Nakamura, Hyang Y. Lee, Hani I. Kuttab, Emma H. Southgate, Stéphane Lezmi, Gee W. Lau, Paul J. Hergenrother

**Affiliations:** 1 Roger Adams Laboratory, Department of Chemistry, University of Illinois at Urbana-Champaign, 600 South Mathews Avenue, Urbana, Illinois 61801, USA; 2 College of Veterinary Medicine, Veterinary Medicine Basic Sciences Building, University of Illinois at Urbana-Champaign, 2001 South Lincoln Avenue, Urbana, Illinois 61802, USA

## Abstract

Fluoroquinolones are one of the most commonly prescribed classes of antibiotics, but fluoroquinolone resistance (FQR) is widespread and increasing. Deoxynybomycin (DNM) is a natural-product antibiotic with an unusual mechanism of action, inhibiting the mutant DNA gyrase that confers FQR. Unfortunately, isolation of DNM is difficult and DNM is insoluble in aqueous solutions, making it a poor candidate for development. Here we describe a facile chemical route to produce DNM and its derivatives. These compounds possess excellent activity against FQR methicillin-resistant *Staphylococcus aureus* and vancomycin-resistant *Enterococci* clinical isolates and inhibit mutant DNA gyrase *in-vitro*. Bacteria that develop resistance to DNM are re-sensitized to fluoroquinolones, suggesting that resistance that emerges to DNM would be treatable. Using a DNM derivative, the first *in-vivo* efficacy of the nybomycin class is demonstrated in a mouse infection model. Overall, the data presented suggest the promise of DNM derivatives for the treatment of FQR infections.

## Abstract (web summary)

Fluoroquinolone antibiotics are widely used to treat serious bacterial infections, but resistance is an increasing problem. Here the authors describe the synthesis and characterization of novel deoxynybomycin derivatives that exhibit activity against fluoroquinolone-resistant infections in an *in vivo* model.

## Graphical abstract

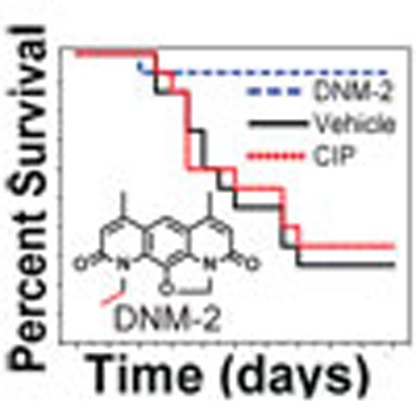

Fluoroquinolones (FQs) were introduced into the clinic in the early 1980s and since then have become one of the most widely prescribed classes of antibiotics^1^,^2^,^3^. Although early FQs were primarily used to treat Gram-negative infections, later generation FQs were also commonly employed against infections caused by Gram-positive pathogens^1^,^4^. FQs are prescribed for severe or antibiotic-resistant urinary tract infections, respiratory tract infections^2^,^4^, gonoccocal infections, tuberculosis and as a prophylactic for anthrax^5^. FQs act by inhibiting bacterial type IIA topoisomerases, specifically DNA gyrase (composed of GyrA and B subunits) and topoisomerase IV (composed of ParC and E subunits). These enzymes catalyse the introduction of negative supercoils and the decatenation of interlinked chromosomes, respectively^6^,^7^,^8^.

Although FQs have demonstrated great utility in the clinic, their widespread use has resulted in significant resistance^4^. Nearly all vancomycin-resistant enterococcus (VRE) and methicillin-resistant *S. aureus* (MRSA) are also resistant to FQs^9^; thus, FQs can no longer be used to treat such infections. FQs are commonly prescribed for *Neiserria gonorrhoeae* and *Pseudomonas aeruginosa* infections, but FQ resistance (FQR) is now observed in a substantial fraction of these isolates, necessitating other treatments^10^. Target-site mutation is the major contributor to FQR^1^,^4^, with high-level resistance observed in bacteria possessing key mutations in both GyrA and ParC^4^. VRE and MRSA both harbour these target-site mutations, with point mutations in the quinolone resistance-determining region (QRDR) of the GyrA subunit of DNA gyrase and the ParC subunit of topoisomerase IV. These mutations alter residues important for the binding of FQs, resulting in an approximately tenfold decrease in binding affinity^11^,^12^. Nearly 100% of MRSA substitute Ser84 of GyrA with Leu^13^,^14^,^15^,^16^,^17^,^18^,^19^,^20^,^21^. Similarly, nearly all FQR VRE substitute Ser83 of GyrA with Ile, Arg or Tyr^22^,^23^,^24^,^25^,^26^.

Nybomycin (NM) is a natural product first identified from a culture of a streptomycete isolated from a Missouri soil sample and found to have antibacterial activity^27^,^28^. During efforts to determine its structure, Rinehart and Renfroe^29^ synthesized a related compound, deoxynybomycin (DNM, Fig. 1a), which was later found to be a natural product and to have more potent activity than NM against a range of bacteria^29^,^30^. More recently, DNM was found to have activity against FQR MRSA with the S84L mutation in GyrA of DNA gyrase^15^. However, isolation of NM and DNM from natural sources is non-trivial 31 and the only reported total synthesis of DNM is very low yielding^32^,^33^. In addition, the low solubility of DNM in any solvent other than concentrated acid presents challenges for its biological evaluation and limits its potential *in-vivo*.

Described herein is an efficient total synthesis of DNM and modifications of this route are used to construct the first DNM derivatives. DNM and several of the derivatives show outstanding antibacterial potency and selectivity against FQR MRSA and VRE clinical isolates. DNM and its derivatives inhibit the mutant DNA gyrase responsible for FQR and resistance to DNM and derivatives results in re-sensitization to FQs, suggesting a resistance cycling that could be useful in the clinic. Finally, using a DNM derivative with superior solubility and pharmacokinetic properties, the first *in-vivo* activity of this class of compounds is demonstrated.

## Results

### Total synthesis of DNM and construction of derivatives

**Figure 1:**
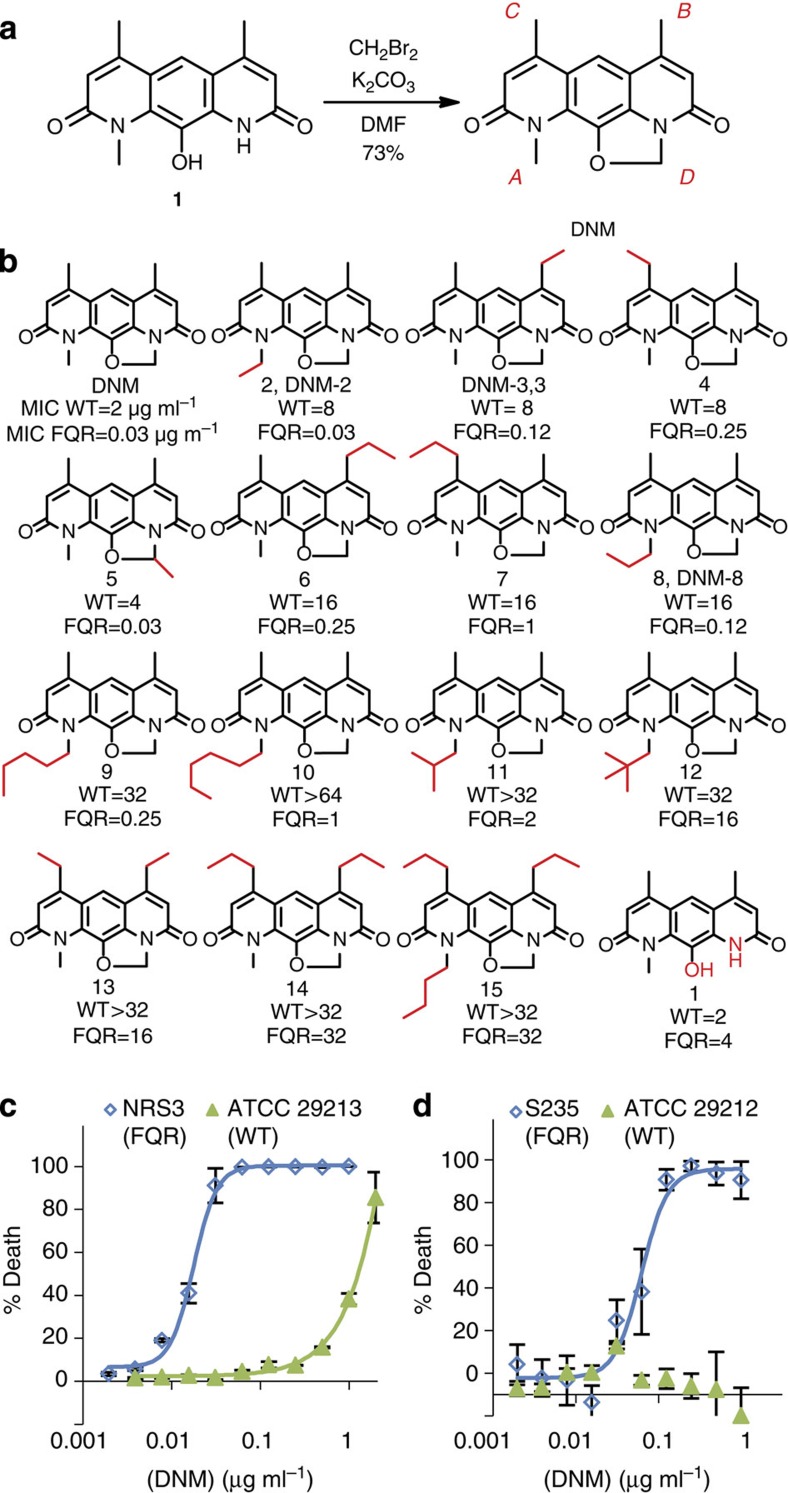
Synthesis and antibacterial activity of DNM and derivatives. (**a**) Final step in the synthesis of DNM. The letters A, B, C and D around the structure of DNM denote sites of derivitization. (**b**) Structure of DNM and derivatives, and their activities against WT *S. aureus* (ATCC 29213, WT) and FQR *S. aureus* (NRS3, FQR). Activity is from three independent replicates of the microdilution broth assay and is reported as the MIC in μg ml^−1^. (**c**) Dose–response curves for FQS *S. aureus* (ATCC 29213) and FQR *S. aureus* (NRS3) treated with DNM. Data shown are from three independent replicates ±s.e.m. (**d**) Dose–response curves for FQS *Enterococcus* (ATCC 29212) and FQR *Enterococcus* (S235) treated with DNM. Data shown are from three independent replicates±s.e.m.

Owing to the documented difficulty of isolating DNM from natural sources^31^, we aimed to develop an efficient, modular and flexible synthesis of DNM that could also be used to construct derivatives. Previously, we reported a synthesis of the natural product deoxynyboquinone (DNQ) that relies on a mixed Suzuki cross-coupling followed by a palladium-catalysed ring closing and deprotection to give diazaanthracenol **1** (Fig. 1a)^34^. To construct DNQ, **1** is oxidized to give the desired quinone^34^. We found that **1** could be converted to DNM in a single step by insertion of the methylene bridge in a reaction inspired by Rinehart's degradation studies and by bridge insertions in similar systems^32^,^33^,^35^. Reaction of **1** with dibromomethane gave DNM in a 73% yield (Fig. 1a). Through this route, DNM was obtained in seven steps with an overall yield of 11%, an improvement over the only other reported total synthesis (0.84% overall yield)^32^,^33^.

This flexible synthetic route also allowed for rapid generation of a variety of derivatives that have not been found as natural products. We hypothesized that addition of alkyl chains would disrupt *π*-stacking between DNM molecules, thus increasing both aqueous and organic solubility, similar to what was observed with DNQ derivatives^36^. By changing the iodoamides used in the Suzuki cross-coupling (see General protocol A in Supplementary Methods), three compounds were synthesized that substituted ethyl for methyl at positions *A*, *B* and *C* (compounds **2**, **3**, and **4**, respectively, Fig. 1b). The derivative with a methyl substitution at *D* was generated by using 1,1-dibromoethane in place of dibromomethane in the final step, to provide compound **5** (Fig. 1b). Other compounds with single sites of derivatization (**6**–**12**) and multiple sites of derivatization (**13**–**15**) were also constructed. Full synthetic routes along with experimental details and characterization data can be found in the Supplementary Methods. Compounds with small alkyl appendages have markedly improved solubility (3- to 13-fold) in pH 7.4 PBS relative to DNM (Supplementary Table 1) and all compounds synthesized have improved dimethyl sulfoxide (DMSO) solubility compared with the parent compound (Supplementary Table 1).

### Evaluation of DNM and derivatives against FQR MRSA and VRE

DNM was evaluated against both FQ-sensitive *S. aureus* (ATCC 29213) and FQR MRSA (NRS3, which has GyrA S84L and ParC S80F). DNM showed modest activity against the FQ-sensitive (FQS) strain 29213. However, DNM showed excellent activity against the FQR NRS3 (Minimum Inhibitory Concentration, MIC=0.03 μg ml^−1^, Fig 1b,c). This MIC compares favourably with standard-of-care treatments for Gram-positive infections including vancomycin (MIC for NRS3=8 μg ml^−1^), daptomycin (MIC for NRS3=8 μg ml^−1^) and linezolid (MIC for NRS3=0.5 μg ml^−1^). The sensitivity of FQR VRE was also explored. DNM had no detectable activity against FQS *Enterococcus* (ATCC 29212, MIC>1.0 μg ml^−1^), but it potently inhibited the growth of FQR VRE (clinical isolate S235, which has GyrA S83I and ParC S80I, MIC=0.125 μg ml^−1^, Fig. 1d). DNM was also evaluated against a panel of Gram-negative bacteria (Supplementary Table 2). It showed no detectable activity against wild-type (WT) or FQR *P. aeruginosa* or *Acinetobacter baumannii*. Moderate activity was seen with a DNM derivative against a permeabilized strain of *Escherichia coli*, suggesting that DNM and its derivatives are unable to penetrate Gram-negative bacteria.

DNM derivatives were evaluated against both FQS *S. aureus* (ATCC 29213) and FQR MRSA (NRS3), and their MIC values are listed in Fig. 1b. Similar to DNM, most derivatives showed significantly enhanced activity against FQR NRS3 compared with FQS 29213. In general, compounds with a single methyl addition retained good activity against NRS3 (**2**–**5**). Further substitution at *B* was relatively well tolerated (**6**), while substitution at *C* was generally less well tolerated (**7**). Compounds possessing longer chains at *A* generally retained potency (**8**–**10**). However, compounds with bulky substitutions at *A* (**11**–**12**), multiple substitutions (**13**–**15**), or without the methylene bridge (**1**) were markedly less active.

### Activity of DNM or derivatives against FQR clinical isolates

**Figure 2:**
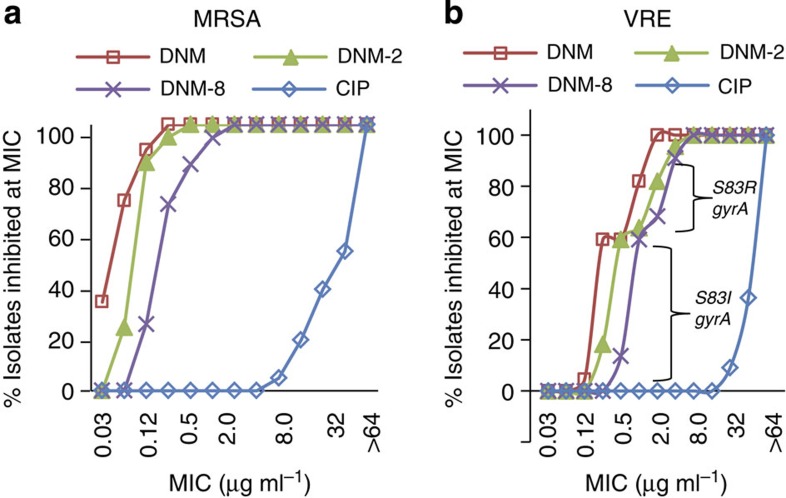
Sensitivity of MRSA and VRE clinical isolates to DNM, DNM-2, DNM-8 and CIP. (**a**) The percentage of MRSA clinical isolates (*n*=21) with an MIC at or lower than the concentration shown. (**b**) The percentage of VRE clinical isolates (*n*=22) with an MIC at or lower than the concentration shown.

DNM and two of the most potent derivatives (DNM-2 and DNM-8) were evaluated against a panel of MRSA and VRE clinical isolates (Figs 2a,b). As shown in Fig. 2, all MRSA and VRE strains were sensitive to these compounds and resistant to ciprofloxacin (CIP). To understand this selectivity, the QRDRs of GyrA and ParC for many of these isolates were sequenced (Supplementary Tables 3 and 4). Although different substitution patterns were found for MRSA ParC, all sequenced strains have the same mutation in GyrA (S84L), consistent with the notion that this mutation sensitizes bacteria to DNM. Similar to the MRSA isolates, the VRE isolates have many different substitutions in ParC, which do not appear to correlate with sensitivity. Unlike the MRSA isolates, the majority of VRE isolates have two different substitutions for GyrA (S83I or S83R). The sensitivity of these strains is affected by this substitution with VRE harbouring the S83I mutation being very sensitive to DNM (MIC =0.125–1 μg ml^−1^) and those with the S83R mutation being less sensitive (MIC ≥1 μg ml^−1^). The activity of the DNM-2 and DNM-8 against these panels of clinical isolates closely mirrors that of DNM (Fig. 2a,b). Full details of the sensitivity of each strain can be found in Supplementary Tables 3 and 4.

### Inhibition of mutant DNA gyrase by DNM and derivatives

**Figure 3:**
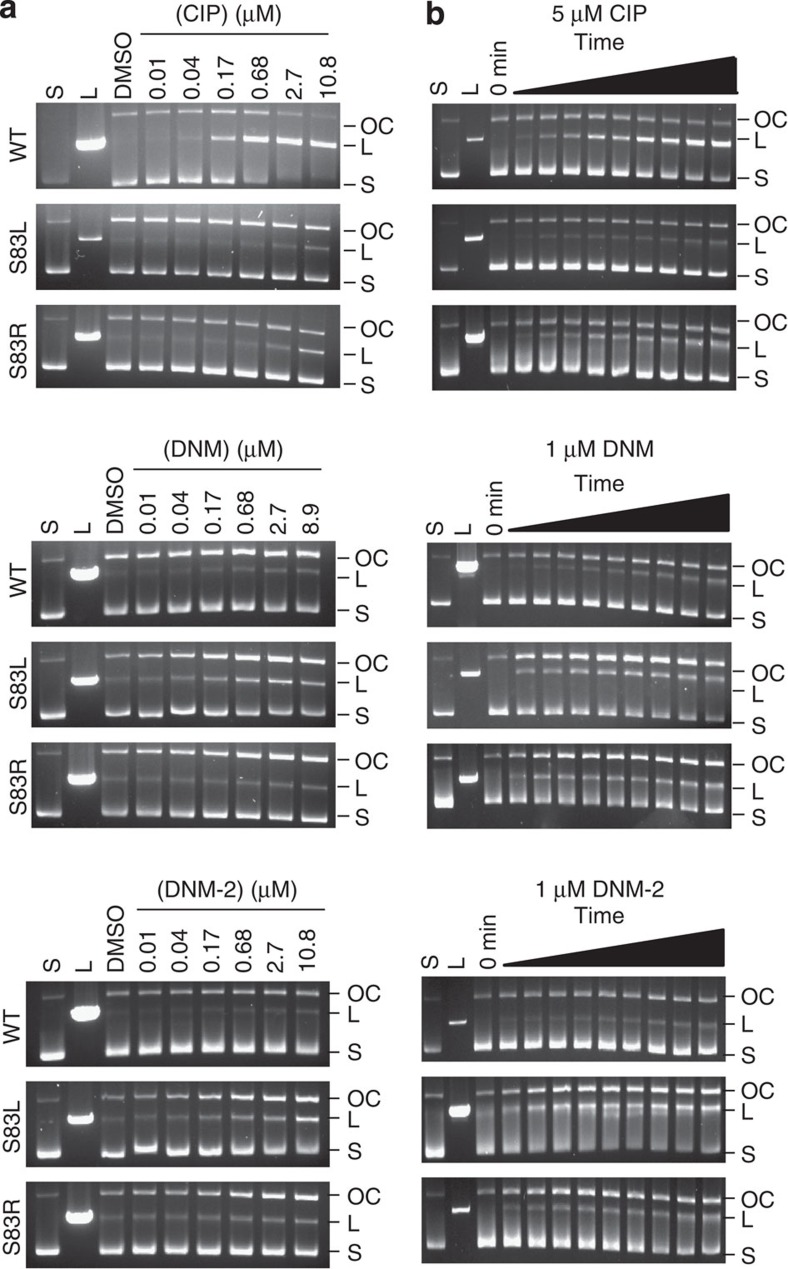
Inhibition of WT and mutant DNA gyrase. (**a**) DNA cleavage assay with WT, S83L and S83R *E. coli* DNA gyrase in the presence of increasing concentrations of CIP, DNM and DNM-2. Concentrations were 0.01, 0.04, 0.017, 0.68, 2.7 and 10.8 μM, except for DNM, which was 8.9 μM for the highest concentration. S, supercoiled; L, linear; OC, open circular or nicked DNA. (**b**) Time course of DNA cleavage with WT, S83L and S83R *E. coli* DNA gyrase in the presence of 5 μM CIP, 1 μM DNM and 1 μM DNM-2. Time points were 0, 1, 3, 5, 10, 15, 20, 30, 60 and 90 min. All gels are representative data from at least three independent experiments.

To further investigate the importance of the GyrA mutation for DNM activity, the ability of DNM, DNM-2 and CIP to inhibit DNA gyrase was determined by using an *in-vitro* DNA cleavage assay. In this assay, DNA gyrase is coincubated with supercoiled DNA and the compound of interest. Inhibition at the cleavage complex of DNA gyrase leads to an increase in either doubly nicked linear (L) DNA (for example, inhibition by CIP^37^) or singly nicked open circular (OC) DNA (for example, inhibition by GSK299423 (ref. 38)). GSK299423 is a recently discovered DNA gyrase inhibitor that is hypothesized to stabilize the DNA–enzyme complex, either pre-cleavage or after the formation of a single-strand break resulting in a buildup of OC DNA^38^. Similar to previous studies, we found that CIP potently inhibits WT DNA gyrase with a greater than sevenfold increase in L DNA being observed at concentrations as low as 0.68 μM (Fig. 3a, full gels in Supplementary Fig. 1, quantification in Supplementary Fig. 2). In addition, in a time-course assay, inhibition of WT DNA gyrase by CIP resulted in a time-dependent buildup of L DNA (Fig. 3b, full gels in Supplementary Fig. 3). Alternatively, when either DNM or DNM-2 was incubated with WT DNA gyrase, neither showed similar increases in L or OC DNA, suggesting that these compounds are poor inhibitors of WT DNA gyrase (Fig. 3a). In addition, during the time-course study with these compounds, buildup of L DNA was only observed at later time points and to a smaller degree (Fig. 3b and Supplementary Fig. 2).

The ability of CIP, DNM and DNM-2 to inhibit S83L or S83R DNA gyrase was then determined. CIP was much less effective at inhibiting either S83L or S83R DNA gyrase compared with WT, with only small increases in L DNA being observed (Fig. 3a). In addition, minimal change was observed with 5 μM CIP at up to 1.5 h (Fig. 3b). Time-course studies performed with an increased concentration of CIP (200 μM) and S83L DNA gyrase revealed a similar pattern of inhibition to that of WT DNA gyrase, suggesting that the residual inhibition goes through a similar mechanism (Supplementary Fig. 4a). DNM and DNM-2 induce only small increases in L DNA with S83L DNA gyrase (Fig. 3a). Instead, DNM inhibition of S83L DNA gyrase led to a significant buildup of OC DNA at 0.17 μM (*P*<0.05), with a similar increase observed for DNM-2 (Fig. 3a and Supplementary Fig. 2). This buildup does not diminish over time (Fig. 3b), suggestive of a mode of inhibition more similar to GSK 299423 than to CIP. Increasing concentrations of DNM-2 to 200 μM and increasing the time up to 2 h confirmed that this OC buildup is not a fleeting event as occurs with CIP (Supplementary Fig. 4b). DNM or DNM-2 inhibition of S83R also led to a buildup of OC DNA similar to that seen with S83L DNA gyrase only at a slightly higher concentration or longer time points, consistent with the activity of these compounds against VRE with the S83R DNA gyrase. Overall, these results are consistent with the clinical isolate data, supporting the critical importance of a mutant DNA gyrase for sensitizing bacteria to DNM. Finally, to determine the selectivity of DNM and derivatives for bacterial DNA gyrase, a decatenation assay with human topoisomerase II was performed. Although doxorubicin inhibited human topoisomerase II at concentrations as low as 3 μM, DNM-2 showed no significant inhibition at concentrations up to 30 μM (Supplementary Fig. 5).

### Development of resistance to DNM

**Figure 4:**
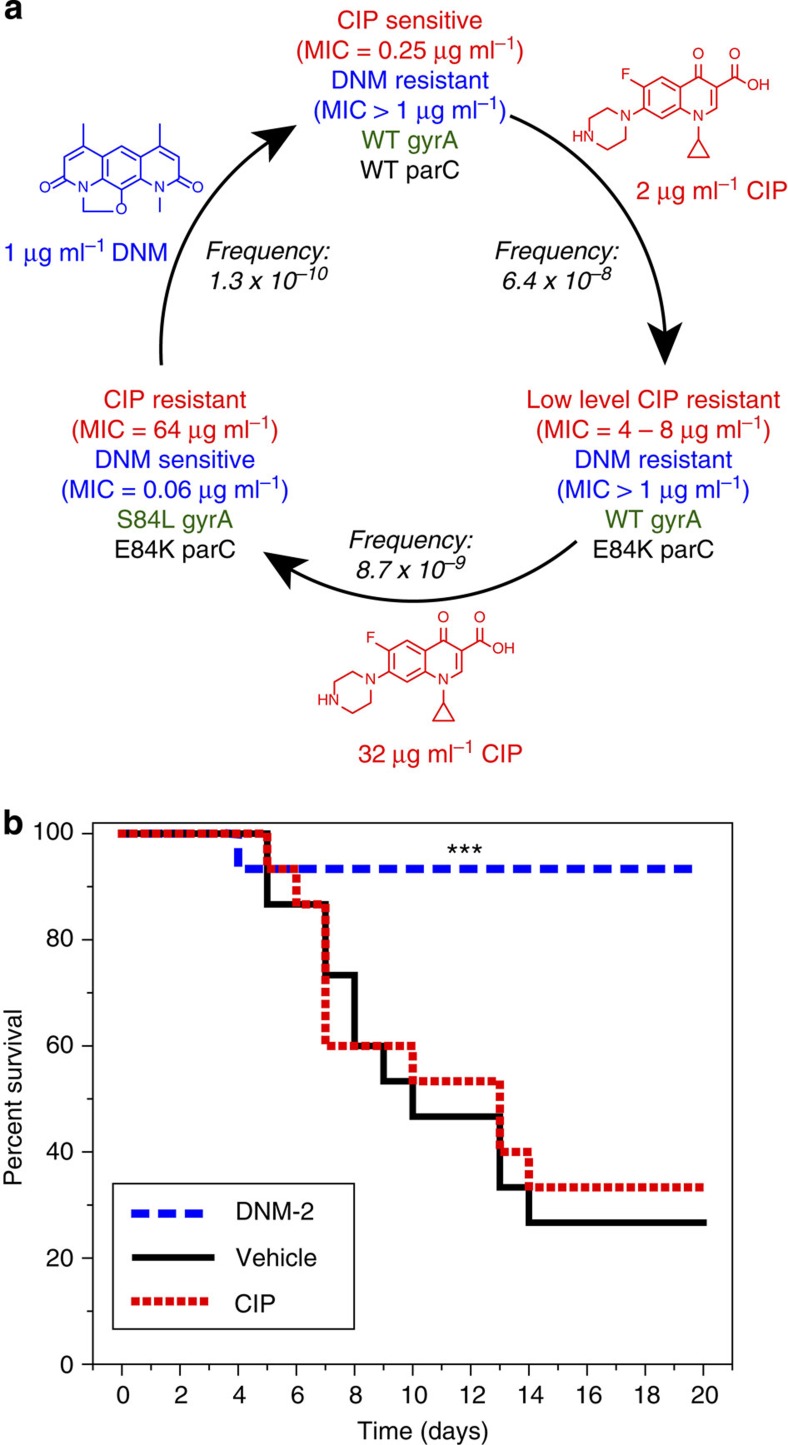
Resistance mechanisms and *in-vivo* activity. (**a**) Representative data of the resistance cycle observed when bacteria are sequentially treated with CIP then DNM. Each strain generated is listed with the MIC of CIP (red), DNM (blue), as well as mutation status of the QRDR of GyrA (green) and ParC (black) shown below. The selection pressure used in each step is shown over the arrow along with the mutation frequency. (**b**) Kaplan–Meier curves showing the survival rates of mice infected with MRSA (NRS3, FQR). The mice received vehicle alone, 50 mg kg^−1^ CIP, or 50 mg kg^−1^ DNM-2 by oral gavage once daily for 10 days; *n*=15 for each group. ***P<0.005 versus vehicle and CIP, log rank survival test.

To explore the development of resistance to both CIP and DNM in *S. aureus*, resistant strains of ATCC 29213 were generated. Consistent with previous reports^39^,^40^, high-level resistance to CIP was generally not achieved in a single step. Instead, low-level resistance (CIP MIC=4–8 μg ml^−1^) was usually achieved with the first step and corresponded to a mutation in ParC (for example, E84K or S80F; Fig. 4a and Supplementary Fig. 6). Unsurprisingly, these low-level resistant strains that do not have the S84L mutation in GyrA were not sensitive to DNM or DNM-2. Development of high-level CIP resistance (CIP MIC=16–64 μg ml^−1^) similar to what is often seen in clinical isolates^13^,^15^,^17^ was observed at the second step and corresponded to an S84L mutation in GyrA. These high-level CIP-resistant strains were extremely sensitive to DNM (MIC=0.03–0.06 μg ml^−1^) and DNM-2 (MIC=0.06–0.12 μg ml^−1^). These FQR bacteria were then exposed to DNM in an effort to create DNM-resistant isolates. The development of DNM resistance in high-level CIP-resistant strains was a rare event, with resistance frequencies ranging from 1 × 10^−10^ to 7 × 10^−10^ (Supplementary Fig. 6). When these strains were found, they showed dramatically improved sensitivity to CIP (MIC=0.25–8.0 μg ml^−1^). All these strains had reverted to WT GyrA (Ser84), with the more CIP-sensitive strains also having WT ParC and the less CIP-sensitive strains retaining ParC mutations (Supplementary Fig. 6). This complete cycle of complementary resistance/sensitivity of CIP and DNM is shown in Fig. 4a and the complete list of resistant strains generated and the sequences of their QRDR is shown in Supplementary Fig. 6.

Resistance development on co-treatment with CIP and DNM-2 was then explored (Supplementary Fig. 7). A low-level CIP-resistant strain (29213-C1) was used in these studies. On treatment with either CIP or DNM-2, resistant colonies were observed. However, no colonies were observed on co-treatment (resistance frequency <1.0 × 10^−10^).

### *In-vivo* efficacy of DNM-2

As a prelude to exploring *in-vivo* efficacy, the toxicity and pharmacokinetic profile of DNM and key derivatives was evaluated. Treatment of red blood cells (RBCs) with DNM and key derivatives indicated that none of these compounds induce haemolysis (Supplementary Fig. 8a). In addition, DNM-2 demonstrated no significant DNA intercalation at concentrations up to 30 μM (Supplementary Fig. 8b). This data combined with previously published data showing that DNM is non-toxic to normal (that is, non-cancerous) cell lines^41^ suggests that these compounds would probably be well tolerated *in-vivo*. Treatment of mice with increasing concentrations of DNM, DNM-2 and DNM-3 showed that all three compounds were well tolerated up to the highest dose evaluated (50 mg kg^−1^ by oral gavage). Pharmacokinetic studies were next performed on DNM, DNM-2 and DNM-3. Although DNM itself showed very low serum exposure (*C*_max_<0.20 μM or 0.060 μg ml^−1^) after a 50-mg kg^−1^ oral dose, DNM-2 showed good bioavailability with a peak serum concentration of 42.6 μM (12.8 μg ml^−1^) and an area under the curve of 44 h μg ml^−1^ (Supplementary Fig. 9 and Supplementary Table 5). DNM-3 showed an intermediate level of bioavailability with a peak serum concentration of 4.3 μM (1.26 μg ml^−1^) and an area under the curve of 4 h μg ml^−1^. The bioavailability of these compounds mirrors the aqueous solubility, suggesting that at least for this limited set of compounds aqueous solubility could be a reasonable predictor of oral bioavailability.

To explore the effect of sustained treatments *in-vivo*, DNM-2 was administered to mice once daily for 10 days (via oral gavage at 50 mg kg^−1^) and markers of haematological and non-haematological toxicity were examined. No clinically significant evidence for myelosuppression, renal injury or hepatic toxicity was identified (Supplementary Table 6). Kidney, brain, lung, liver, spleen, heart, and stomach sample examinations also did not reveal any evidence of toxicity. In small intestine sections, mild intestinal dilation associated with villi atrophy were noted (Supplementary Fig. 10). Increased vacuolation of white and brown adipocytes with a minimal increase in triglyceride levels were noted as well. These changes were probably indirectly related to the drug and possibly due to the antibiotic effects on the intestinal flora. As none of the mice showed any clinical symptoms, these changes were considered of minimal significance. With this indication that DNM-2 offered good exposure on oral dosing with no observable toxicity, an *in-vivo* model of mouse sepsis was conducted. Mice were infected with FQR MRSA (NRS3) via tail vein injection. Mice were treated with CIP (50 mg kg^−1^, oral gavage), DNM-2 (50 mg kg^−1^, oral gavage) or vehicle control once daily for 10 days. As shown by the Kaplan–Meier survival curve in Fig. 4b, mice treated with DNM-2 showed a significant survival difference relative to both CIP and vehicle-treated control (*P*<0.005, Fig. 4b).

## Discussion

Using a synthetic route building on a palladium-catalysed mixed cross-coupling and a methylene bridge insertion, hundreds of milligrams of the natural-product DNM were prepared as described herein. The modular nature of the synthesis also allows access to non-natural DNM derivatives, many of which display similar antibacterial efficacy but with significantly better solubility properties than the parent. Specifically, small alkyl appendages greatly improve aqueous solubility: DNM has aqueous solubility of only 9 μM compared with 121 μM for DNM-2. This improved solubility is probably because of the ability of the short alkyl chains to break up *π*-stacking similar to what was observed with derivatives of DNQ^36^,^42^. Longer or more alkyl chains do not display similar increases in aqueous solubility probably because of the increased hydrophobicity of these compounds. With these compounds in hand, a structure–activity relationship was established and derivatives were found to have comparable potencies to the parent against FQR MRSA and VRE in cell culture and against mutated DNA gyrase *in-vitro*.

The major mechanism of FQR for bacteria involves the mutation of FQ targets DNA gyrase and topoisomerase IV. Although nearly all FQR bacteria found to date have such mutations, the exact mutation can vary based on the bacterium. For MRSA, nearly 100% have the S84L mutation in DNA gyrase 13,14,15,16,17,18,19,20,21. Similarly, *Bacillus anthracis*, *E. coli* and *A. baumannii* also have the analogous serine mutated to leucine^43^,^44^,^45^. In VRE the serine is mutated to multiple different residues (Ile, Arg and Tyr), while in *Streptococcus pneumoniae*, *Klebsiella pneumonia* and *N. gonorrhoeae* this Ser is often changed to either Phe or Tyr^22^,^23^,^24^,^25^,^26^,^46^,^47^,^48^. *P. aeruginosa* differs in that it naturally has a Thr instead of the Ser. However, *P. aeruginosa* is similar to VRE in that the Thr is mutated to an Ile in FQR strains. We now show that DNM has excellent activity versus Ser→Leu or Ser→Ile mutants with moderate activity in Ser→Arg strains, with these cell culture results correlating with the potencies of the compounds in *in-vitro* assays with DNA gyrase and mutants. The ability of DNM to target these different mutants suggests that this natural product or its derivatives could be broadly applicable against FQR bacteria regardless of the exact nature of the Ser mutation.

FQs inhibit DNA gyrase causing double-stranded breaks that appear as a buildup of L DNA in a DNA gyrase cleavage assay (Fig. 3 and ref. 37). Kampranis and Maxwell^37^ have demonstrated that FQs initially stabilize a single phosphotyrosine bond as evidenced by an initial buildup of OC DNA, also observed in Fig. 3b. However, the FQ stabilization of a single-strand break causes an even faster second cleavage event that is also stabilized by FQs, thus explaining the rapid buildup of linear DNA^37^. Another DNA gyrase inhibitor, GSK299423, acts via a different mechanism^38^. Unlike FQs that bind within the two active sites, it binds between the active sites stabilizing either an uncleaved or a single-stranded cleaved DNA. The stabilization induced by GSK299423 differs from that of CIP in that it does not result in a second cleavage event and instead causes a buildup of OC DNA. The more potent activity of DNM against FQR-mutant DNA gyrase suggests that DNM probably binds similarly to FQs, near the mutated residues and thus near the two active sites. Despite this similarity in binding position, the phenotype of DNM in the DNA cleavage assay (that is, the buildup of OC DNA) suggests that its overall mode of inhibition is more similar to that of GSK299423. The mutational status of ParC does not affect sensitivity to DNM, as shown by the data from the clinical isolates and resistance mutants.

In this study, we showed that an entire resistance/sensitization cycle is possible beginning with *S. aureus* (ATCC 29213) that is FQS/DNM resistant. After multiple rounds of selection against CIP, a FQR/DNM-sensitive strain was generated. Next, after a selection round with DNM, a FQS/DNM-resistant strain was found. These results suggest intriguing clinical possibilities for DNM, either alone or in combination with FQs. As surveillance data show the ubiquity of FQR in MRSA and VRE, a DNM compound could be an outstanding therapeutic option for these infections; indeed, a new orally available treatment for MRSA and VRE is a well-recognized clinical need^49^ and would be a welcome addition to the antibiotic arsenal. As resistance to DNM emerges, the data predict that such bacteria would be sensitive to FQs. At this point, a diagnostic test could be used to choose between FQ or DNM, or a co-treatment with both a FQ and DNM would be possible. As reported herein, co-resistance was not generated in cell culture, with no colonies being observed on treatment with 4 μg ml^−1^ CIP and 6 μg ml^−1^ DNM-2.

Before this study, little to no data existed about the administration of DNM to animals. However, a related but less potent compound NM (*C*=CH_2_OH) has been examined in mice. It was found to be well tolerated when dosed either subcutaneously, orally or by intraperitoneal injection^27^. However, it showed no activity in mice infected with various bacteria (*K. pneuomoniae*, *S. aureus* or *Mycobacterium tuberculosis*), leading Brock and Sokolski^27^ to suggest that this high tolerability and lack of efficacy is probably a result of the very poor solubility of NM (similar to DNM, it is only soluble in concentrated acid) and thus lack of absorption. We demonstrated that DNM also has a high maximal tolerated dose (>50 mg kg^−1^ oral gavage), but pharmacokinetic analysis indicate that it is not absorbed to any appreciable degree. However, DNM-2, which has improved solubility, was also very well tolerated (maximal tolerated dose >50 mg kg^−1^) and showed favourable pharmacokinetic properties when dosed orally. In addition, in this study we found that orally administered DNM-2 is effective in treating mice infected with MRSA, thus showing the first *in-vivo* efficacy for this class of compounds.

FQR pathogens are now a significant medical problem and the data presented herein reveal the considerable translational potential of DNM derivatives, including the following: (1) a short and efficient synthetic route has been developed that can readily supply large amounts of compound. (2) DNM-2 has outstanding pharmacokinetic (PK) properties with a peak serum concentration (∼50 μM when given at 50 mg kg^−1^ orally) far exceeding the MIC. (3) DNM-2 is extremely well tolerated in mice, with no signs of toxicity at the dose levels tested. This is consistent with our data showing these compounds do not induce haemolysis or inhibit human topoisomerase II. (4) DNM-2, when given orally, has outstanding efficacy in a mouse model of MRSA infection. An oral drug for MRSA and VRE is a well-recognized clinical need and DNM-2 has tremendous promise in this regard. (5) Finally, resistance to DNM is much more difficult than resistance to CIP, as shown by the resistance frequencies in Fig. 4a. A problem with novel antibacterials is that bacterial resistance typically necessitates the development of a new drug to treat those drug-resistant pathogens. However, when the inevitable resistance to DNM/derivatives does arise clinically, these bacteria will be sensitive to FQs, a widely used and well-understood class of antibiotics.

## Methods

### Synthesis of DNM and derivatives

Synthesis of the diazaanthracenols of DNM and the derivatives and chemical characterizations are described in the Supplementary Methods. The NMR spectra are shown in Supplementary Note 1.

### Aqueous solubility determination

Initially, a small amount of solid compound (generally 0.5–1.5 mg) was measured into a 1.7-ml Eppendorf tube. PBS (pH 7.4) was added to give a maximum final concentration of 1 mg ml^−1^ of compound. The compound was vortexed for ∼30 s before being placed into a bath sonicator (Cole Parmer, ultrasonic cleaner) for 1 h. Longer incubation times (up to 24 h) were performed with select compounds and no difference in solubility was observed; thus, 1 h was used for all subsequent testing. The tubes were vortexed again for 30 s before being centrifuged at maximum speed (13,000* × g*) for 10 min. The supernatant was then filtered through a 0.22-μm syringe filter (Millipore Millex MP). The filtrate was then analysed by liquid chromatography–mass spectrometry (*λ*=254 nm, electrospray ionization–time-of-flight in positive mode, Agilent Technologies 6230 TOF LC/MS). The filtrate was diluted 1:2 and 1:4 and all three samples (1 × , 0.5 × and 0.25 × ) were analysed in triplicate. Three independent replicates of each compound were performed. A calibration curve for each compound was generated from 1 to 40 μM by dissolving the compound in DMSO and making dilutions of the stock in DMSO. The calibration curve (measured by ultraviolet absorbance) was linear over this range. The concentration of the samples was calculated based on the calibration curves.

### Bacterial strains

MRSA and *P. aeruginosa* isolates were from Cubist Pharmaceuticals (Lexington, MA). VRE isolates were from a previously published collection^50^. *E. coli* strains were obtained either from ATCC or Professor Cari Vanderpool (UIUC). *A. baumannii* isolates were obtained from Dr John Quale^51^.

### Antibiotic susceptibility tests

Susceptibility testing was performed in triplicate, using the microdilution broth method as outlined by the Clinical and Laboratory Standards Institute^52^. Mueller Hinton (MH) broth was used.

### DNA amplification and sequencing analysis

A single colony of *S. aureus* grown on MH agar or a single colony of *Enterococcus* grown on Brain-Heart Infusion (BHI) agar was suspended in 50 μl of the PCR mixture containing the primers (Supplementary Table 7) and PCR master mix (Platinum *Taq*DNA Polymerase, Invitrogen). PCR amplification was performed using an initial denaturation step of 94 °C for 2 min followed by 35 cycles of 94 °C for 30 s, 52 °C (*S. aureus*. or *Enterococcus faecalis*) or 58 °C (*Enteroccocus faecium*) for 30 s, 72 °C for 50 s. PCR products were purified further on a 1% agarose gel and DNA was extracted (QIAquick Gel Extraction Kit, Qiagen). DNA sequencing was performed by the W. M. Keck Center for Comparative and Functional Genomics (UIUC). The NCBI standard nucleotide BLAST database was used to verify the identity of the PCR products and determine mutations within the sequences.

### Site-directed mutagenesis

pTRCHisA-GyrA plasmid containing the gene for *E. coli* gyrase A was kindly provided by Professor David Hooper^53^. Primers for mutagenesis were designed using QuikChange Primer Design (Agilent) and their sequences can be found in Supplementary Table 7. Site-directed mutagenesis was carried out with the QuikChange Lightning Site-Directed Mutagenesis Kit (Agilent), according to the manufacturer's instructions, with the modification that NEB Turbo Competent *E. coli* were used as the host strain. All clones were confirmed by sequencing.

### DNA gyrase expression

Expression of WT *E. coli* gyrase A and gyrase B was performed as previously described^53^. Briefly, pTRCHisA-GyrA, pTRCHisA-GyrAS83L, pTRCHisA-GyrAS83R, or pTRCHisA-GyrB were introduced into One Shot BL21 Star (DE3) (NEB) by chemical transformation. Transformed cells were selected for on an Luria-Bertani (LB) ampicillin plate. Single colonies from a fresh plate were inoculated into 50 ml of LB with 50 μg ml^−1^ ampicillin and incubated aerobically at 37 °C with shaking at 250 r.p.m. overnight (14–16 h). The overnight culture was then used to inoculate 1 litre LB with 50 μg ml^−1^ ampicillin. The culture was grown aerobically with shaking at 250 r.p.m. until *A*_600_ reached 0.4–0.6. Protein expression was induced with a final concentration of 0.5 mM of IPTG (isopropyl-β-D-thiogalactoside) at 37 °C with shaking at 250 r.p.m. for 4 h. The culture was harvested by centrifugation at 5,000* × g* for 5 min at 4 °C. Cell pellets were frozen at –20 °C, thawed on ice for 30 min and resuspended in TGN_150_ (20 mM Tris-HCl pH 7.5, 10% glycerol, 150 mM NaCl) with 0.5 mg ml^−1^ lysozyme with 2 μg ml^−1^ aprotinin, 1 μg ml^−1^ leupeptin, 1 μg ml^−1^ pepstatin A and 100 μM phenylmethanesulfonylfluoride. Cells were lysed by sonication at 35% amplitude (10 s pulse with 30 s rest, 6 times). The lysate was cleared by centrifugation at 35,000* × g* for 30 min at 4 °C. The supernatant was batch loaded onto 1 ml of 1:1 Ni-NTA agarose (Qiagen) at 4 °C for 30 min with inversion. The resin was washed with 20 ml TGN_150_ with 10 mM imidazole followed by 10 ml of wash buffer (20 mM Tris-HCl pH 7.5, 10% glycerol, 300 mM NaCl, 10 mM imidazole) and eluted with TGN_150_ containing imidazole concentrations of 25, 50, 100, 200, 300 and 500 mM. Eluted fractions were assessed by SDS–PAGE, using 4–20% TGX Mini-PROTEAN gels (Bio-Rad). Fractions containing pure protein were pooled and dialysed against TDEN buffer (50 mM Tris-HCl pH 7.5, 5 mM dithiothreitol (DTT), 1 mM EDTA, 150 mM NaCl) overnight at 4 °C, using a Slide-A-Lyzer Dialysis Cassette, 10 000 MWCO (Thermo Scientific) and concentrated to ∼0.5–1 ml, using an Amicon Ultra-15 50 K Centrifugal Filter Device. The concentration was determined by Bradford assay (Sigma) using BSA (Thermo Scientific) as the control. Expression of S83L and S83R GyrA was performed identically to expression of the WT GyrA.

### DNA gyrase cleavage

DNA gyrase cleavage assays were performed as previously described with minor changes^12^,^37^,^54^. Ten micrograms per millilitre supercoiled DNA (pBR322, Inspiralis) was added to buffer (35 mM Tris-HCl pH 7.5, 24 mM KCl, 4 mM MgCl_2_, 2 mM DTT, 1.8 mM spermidine, 6.5% glycerol, 0.1 mg ml^−1^ albumin) with compound or vehicle. Compound concentrations were 0.01, 0.04, 0.17, 0.68, 2.7 and 10.8 μM, except for DNM, which was 8.9 μM for its highest concentration. DNA gyrase was added to a final concentration of 16 nM gyrA and 32 nM gyrA (giving a final concentration of *A*_2_*B*_2_ of 8 nM) for 25 min at 30 °C. Linear product was revealed by addition of 0.2% SDS and 0.1 μg ml^−1^ proteinase K for 30 min at 37 °C. DNA loading dye (Thermo Scientific) was added to the samples and they were run on 1% agarose gels containing 0.5 μg ml^−1^ ethidium bromide. Gels were imaged on a Molecular Imager Gel Doc XR+ (Biorad) and bands were quantified using ImageJ^4^. Per cent of type of DNA was calculated with total DNA in each lane being 100%. For time-course cleavage assays, the same protocol was followed, except that the initial incubation was for varying times (0, 1, 3, 5, 10, 15, 20, 30, 60, 90, 120 and 180 min) instead of 25 min.

### Human topoisomerase decatenation assay

The decatenation assay was performed with the Human Topo II Decatenation Assay Kit (Inspiralis), according to the manufacturer's instructions, with minor modifications. First, a master mix was made containing 2 μl of 10 × assay buffer (500 mM Tris-HCl pH 7.5, 1250, mM NaCl, 100 mM MgCl_2_, 50 mM DTT, 1,000 μg ml^−1^ albumin), 0.67 μl of 30 mM ATP, 1.34 μl of 0.1 ng μl^−1^ kDNA, and 14.3 μl of nuclease-free water per sample is made. DMSO or 30 × compound is added to a 0.5-ml Eppendorf tube (0.67 μl per tube). The master mix is then added to each tube (18.3 μl per tube). Finally, 1 U of human topoisomerase (1 μl of 1 U μl^−1^ stock) is added to each tube for a final volume of 20 μl. The tubes are then incubated at 37 °C for 30 min. Reactions are stopped by the addition of 20 μl of 24:1 chloroform:isoamyl alcohol and 20 μl of stop dye (40% sucrose, 1 mM EDTA, 100 mM Tris-HCl pH 7.5, 0.5 μg ml^−1^ bromophenol blue). Samples were run on 1% agarose gels containing 0.5 μg ml^−1^ ethidium bromide for 1 h at 110 V or until the dye front was approximately halfway down the gel. Gels were imaged on a Molecular Imager Gel Doc XR+ (Biorad).

### Resistant mutant generation

Agar plates (15 cm) were prepared containing MH broth and antibacterial compounds at concentrations detailed in the Supplementary Fig. 6. Forty millilitres of an overnight bacterial culture was centrifuged at 3,000 × g for 10 min and resuspended in 0.4 ml of sterile PBS. Plates were inoculated with 100 μl of bacteria in PBS by spreading with beads. Inoculated plates were then incubated at 37 °C for 72 h and the number of resistant colonies was counted. To determine the number of viable colonies spread onto each plate, dilutions of the overnight culture in sterile PBS were spread onto non-selective MH agar plates and plates were incubated overnight at 37 °C before counting colonies.

### *In-vitro* haemolysis assay

Haemolysis assays were performed as previously described^55^. Briefly, assays were performed using human erythrocytes within 3 days of receipt. One millilitre of human blood purchased from Bioreclamation, Inc. (Hicksville, NY) was centrifuged (10,000* × g*, 2 min). The pellet was washed three times with sterile saline (0.9% NaCl in water) by repeated gentle suspension and centrifugation. The pellet was resuspended in RBC buffer (10 mM Na_2_HPO_4_, 150 mM NaCl, 1 mM MgCl_2_ pH 7.4). To evaluate haemolytic activity of DNM and derivatives, 1 μl either 3.2 mg ml^−1^ DMSO stock (or the most concentrated stock of the compound available if not soluble at 3.2 mg ml^−1^ in DMSO) was transferred to 0.5 ml Eppendorf tubes containing 19 μl RBC buffer. Negative control tubes contained 1 μl DMSO and 19 μl RBC buffer, and positive control tubes contained 1 μl DMSO and 19 μl sterile deionized water. A suspension of washed erythrocytes (10 μl) was added to each tubes and samples were incubated at 37 °C for 2 h. Samples were centrifuged at 10,000* × g* for 2 min and the supernatants from each sample (25 μl) were transferred to a clear, sterile 384-well plate. The absorbance of these supernatants was measured at 540 nm using a SpectraMaxPlus384 absorbance plate reader (Molecular Devices). Per cent haemolysis of each sample was calculated relative to the average absorbance values measured for positive controls.

### Intercalation assay

Intercalation assays were performed as previously described^34^. The ability of DNM-2 to intercalate into DNA was measured by an ethidium bromide displacement assay. Herring Sperm DNA (34 μg ml^−1^ final) was premixed with buffer containing ethidium bromide (50 mM Tris base, 100 mM NaCl, 1 mM EDTA, 5 μM EtBr pH 7.5). Ninety-five microlitres of this solution was added to a 96-well plate containing 5 μl of DMSO solutions of compounds. In addition to vehicle controls, wells lacking either DNA or EtBr were also used to ensure that these did not have an effect on fluorescence. Doxorubicin was used as a positive control. The reactions were allowed to incubate for 30 min. Fluorescence was then read on a Gemini microplate reader (Molecular Devices, excitation=545 nm, emission=595 nm).

### Pharmacokinetic assessment

The animal studies (PK, *in-vivo* toxicity and *in-vivo* efficacy) were carried out in strict accordance with the recommendations in the Guide for the Care and Use of Laboratory Animals of the National Institutes of Health. The protocol was approved by the Institutional Animal Care and Use Committee (IACUC) at the University of Illinois at Urbana-Champaign (Protocol Number: 13406). In these studies, 10- to 12-week-old female C57BL/6 mice purchased from Charles River were used. DNM, DNM-2 and DNM-3 were formulated as slurries at 8.3 mg ml^−1^ in 25% Cremophor RH40/water (v/v). Before beginning the pharmacokinetic assessment, mice were first tested for their ability to tolerate the DNM, DNM-2 and DNM-3 at 50 mg kg^−1^ (p.o.). After establishing that this dose was well tolerated, mice were treated with DNM, DNM-2 or DNM-3 (all 50 mg kg^−1^) via oral gavage, with three mice per time point (15, 30, 60, 120, 240 and 480 min). At specified time points, mice were killed and blood was collected, centrifuged and the serum was frozen at −80 °C until analysis. The proteins in a 50-μl aliquot of serum were precipitated by the addition of 50 μl of acetonitrile and the sample was centrifuged to remove the proteins. Serum concentrations of DNM, DNM-2 and DNM-3 were determined by HPLC. PK parameters were determined using GraphPad Prism Version 5.00 for Windows.

### *In-vivo* toxicity assessment

The protocol was approved by the IACUC at the University of Illinois at Urbana-Champaign (Protocol Number: 14032). Six-week-old male pathogen-free BALB/c mice were purchased from Taconics Biosciences (Albany, NY). All animals were housed in a pathogen-free environment and received sterile food and water. Mice (*n*=5) were treated once daily for 10 days, with 50 mg kg^−1^ DNM-2 or vehicle (25% Cremophor RH 40/PBS (v/v)), by oral gavage. Toxicity was assessed as previously described^56^. Specifically, heparinized whole blood was collected for assessment of total white blood cells, neutrophils, lymphocytes, haematocrit, platelets, creatinine, blood urea, nitrogen, albumin, alanine aminotransferase, alkaline phosphatase and total bilirubin. Mice were euthanized by overdosing with ketamine/xylazine and the heart, lung, kidney, liver, spleen, gastrointestinal tract and the brain were collected for histopathology. Tissue samples were fixed 24 h in 10% neutral buffered formalin, processed and paraffin embedded, sectioned (5 μm thickness) and stained with haematoxylin and eosin. All slides were systematically evaluated by a single board-certified veterinary anatomic pathologist (SL) for evidence of acute or chronic inflammation and toxicity. All lesions were characterized, recorded and scored for severity (minimal=1, mild=2, moderate=3, marked=4, and severe=5).

### *In-vivo* efficacy

The protocol was approved by the IACUC at the University of Illinois at Urbana-Champaign (Protocol Number: 14032). Six-week-old male pathogen-free BALB/c mice were purchased from Harlan Sprague-–Dawley (Indianapolis, IN). All animals were housed in a pathogen-free environment and received sterile food and water. For the inoculation, overnight cultures of *S. aureus* clinical isolate NRS3 were diluted 1:100 into fresh tryptic soy broth and grown for 2 h at 37 °C. Bacteria were washed and resuspended in sterile PBS. The mice were anaesthetized with ketamine and xylazine. The mouse tails were pre-warmed in 45 °C for 5 min before 1.2 × 10^8^ colony-forming units of *S. aureus* in 50 μl of PBS were injected into a tail vein using a 29-gauge needle. This number of bacteria was determined from a series of preliminary studies in which groups of mice were infected with a range of 10^6^–10^9^ colony-forming units of *S. aureus*. Infected mice (15 mice per group) were then treated once daily for 10 days with 50 mg kg^−1^ DNM-2, 50 mg kg^−1^ CIP or vehicle (25% Cremophor RH 40/PBS (v/v)), by oral gavage. For survival analyses, a Kaplan–Meier Log Rank Survival Test was performed using OriginPro 9 (Northampton, MA).

## Author contributions

P.J.H., E.I.P. and J.S.B. conceived the study. J.S.B. designed the synthesis of DNM and all derivatives, except compound **5**. E.I.P. and E.H.S. designed the synthesis of derivative **5**. E.I.P., B.A.N., H.I.K. and E.H.S. executed the synthesis of the compounds used in this study. E.I.P. designed and executed the biological experiments including MIC determinations, sequencing of the QRDR of clinical isolates, *in vitro* analysis of DNA gyrase inhibition and development of resistant mutants. H.Y.L. performed the pharmacokinetic analysis. G.W.L. designed and executed the mouse model of infection. S.L. performed clinical pathology and histopathology toxicity evaluations. P.J.H. and E.I.P. analysed the data and wrote the manuscript, with the assistance of J.S.B., B.A.N., H.Y.L. S.L., and G.W.L.

## Additional information

**How to cite this article**: Parkinson, E. I. *et al*. Deoxynybomycins inhibit mutant DNA gyrase and rescue mice infected with fluoroquinolone-resistant bacteria. *Nat. Commun*. 6:6947 doi: 10.1038/ncomms7947 (2015).

## Supplementary Material

Supplementary Information Supplementary Figures 1-10, Supplementary Tables 1-7, Supplementary Note 1, Supplementary Methods and Supplementary References
