# Supplementary material for: Deoxynybomycins inhibit mutant DNA gyrase and rescue mice infected with fluoroquinolone-resistant bacteria
Source: Nat Commun. 2015 Apr 24;6:6947. doi: 10.1038/ncomms7947 (PMC4421842; doi:10.1038/ncomms7947)
Supplement: Supplementary Information — Supplementary Figures 1-10, Supplementary Tables 1-7, Supplementary Note 1, Supplementary Methods and Supplementary References [file ncomms7947-s1.pdf]

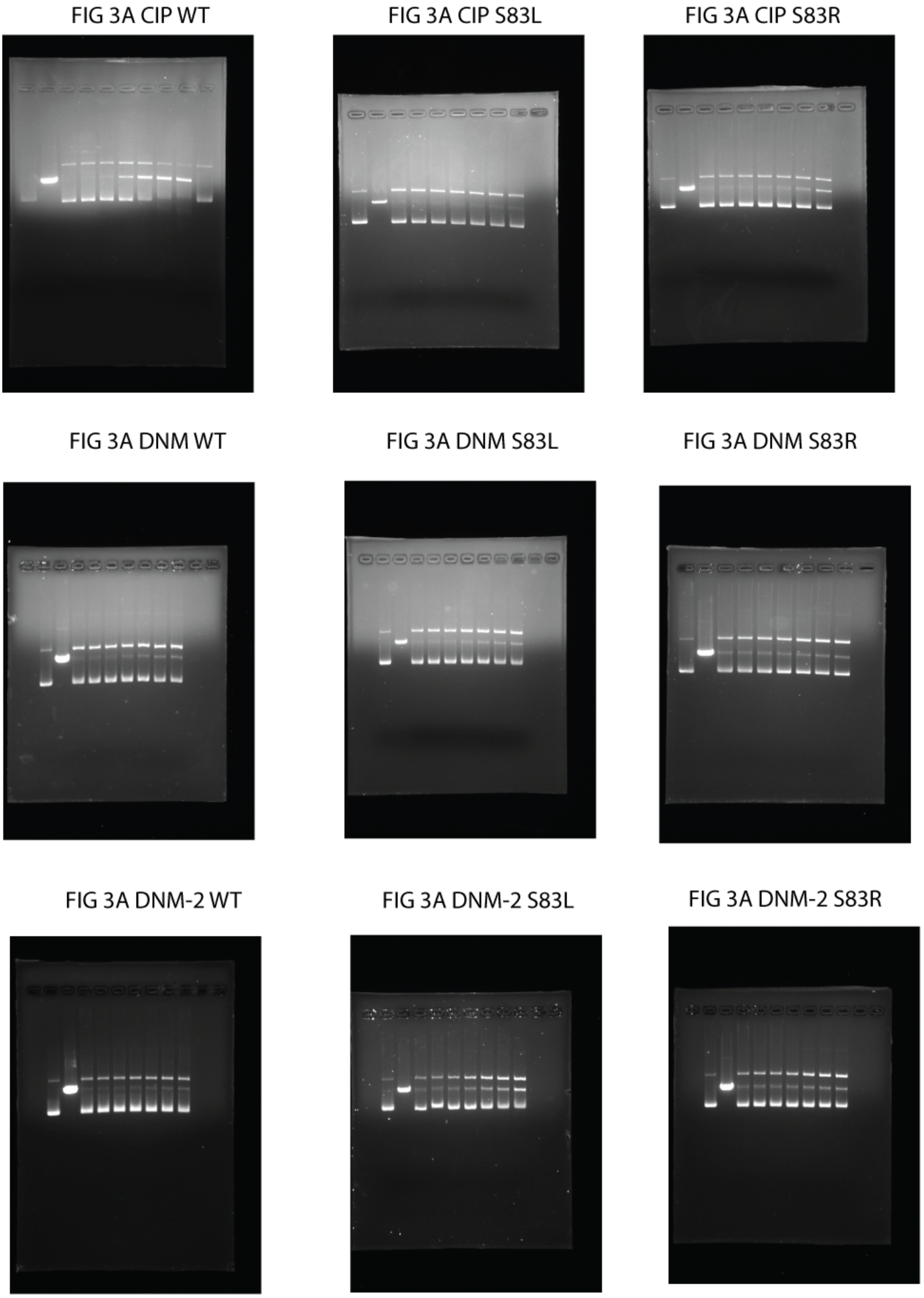

Supplementary Figure 1. Full gels from Figure 3A

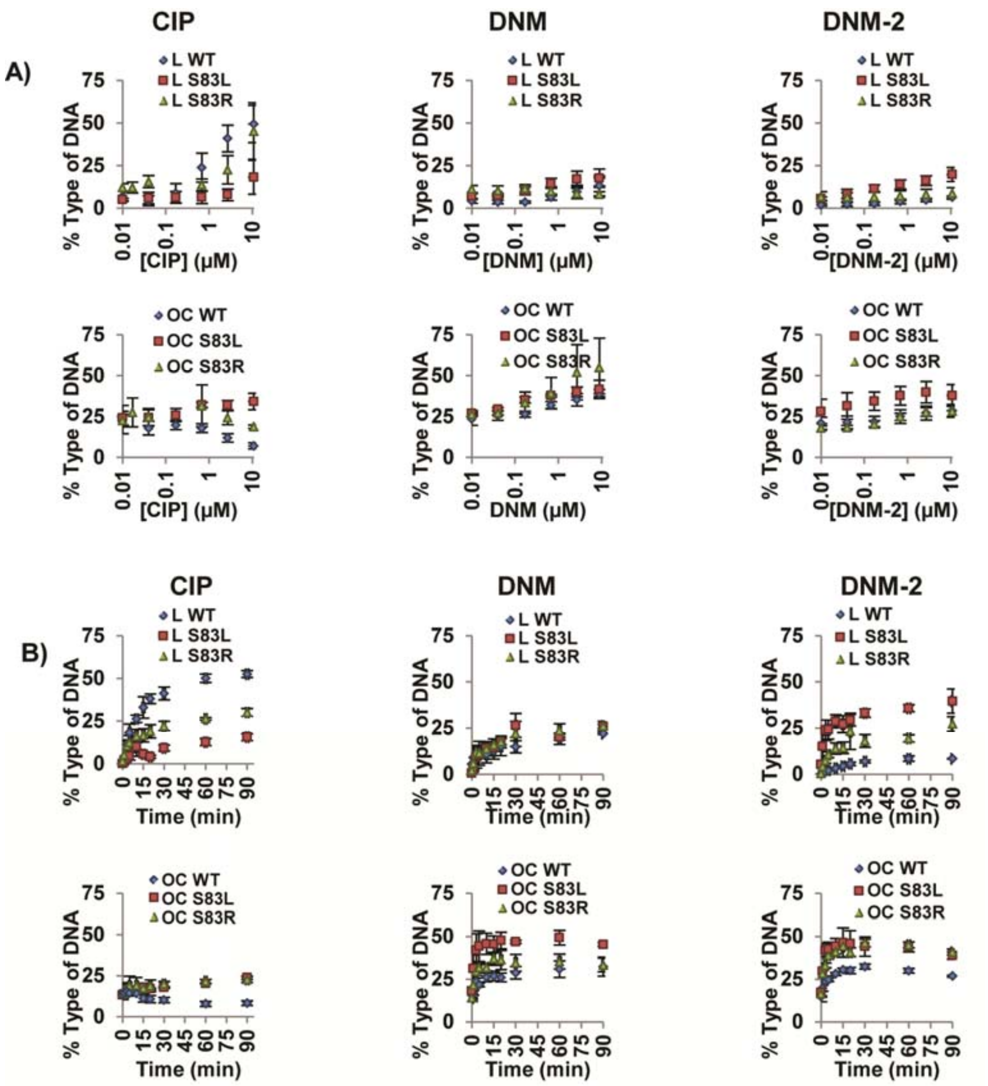

Supplementary Figure 2. Quantification of gels from Figure 3 (Inhibition of wt and mutant DNA gyrase) *(A)* Quantification of DNA cleavage assay with WT, S83L, and S83R *E. coli* DNA gyrase in the presence of increasing concentrations of CIP, DNM, and DNM-2. Concentrations were 0.01, 0.04, 0.017, 0.68, 2.7, and 10.8 µM except for DNM which was 8.9 µM for the highest concentration. Top row is the quantification of the linear band and bottom row is quantification of the open circular (nicked) band. *(B)* Quantification of time course of DNA cleavage of 5 µM CIP, 1 µM DNM, and 1 µM DNM-2 with WT, S83L, and S83R DNA gyrase. Time points were 0, 1, 3, 5, 10, 15, 20, 30, 60, and 90 min. Top row is the quantification of the linear band and bottom row is quantification of the open circular (nicked) band. Data shown is from three independent replicates ± SEM. Quantification was performed using ImageJ software.

FIG 3B CIP WT

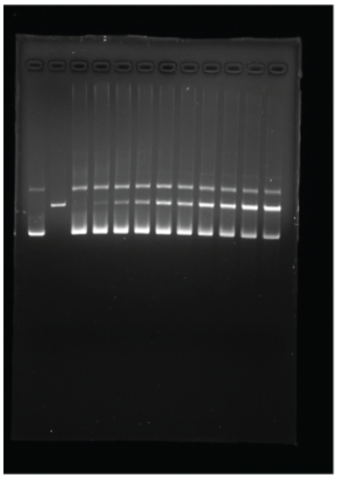

FIG 3B CIP S83L

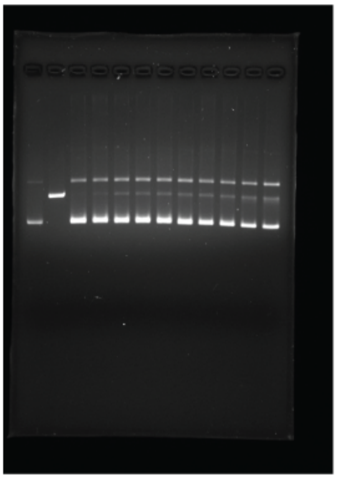

FIG 3B CIP S83R

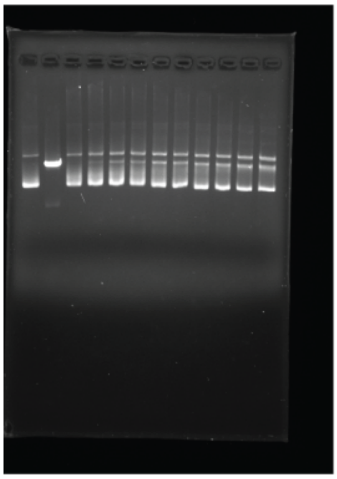

FIG 3B DNM WT

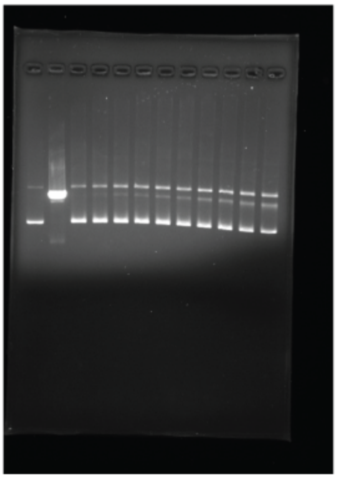

FIG 3B DNM S83L

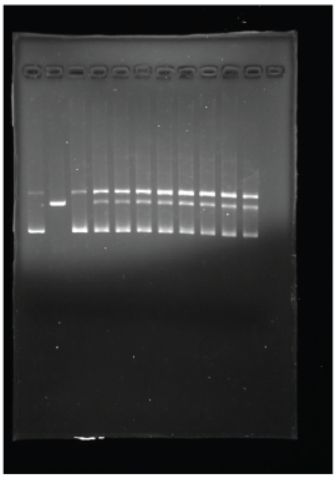

FIG 3B DNM S83R

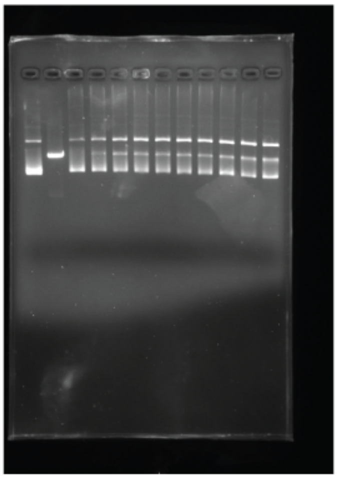

FIG 3B DNM-2 WT

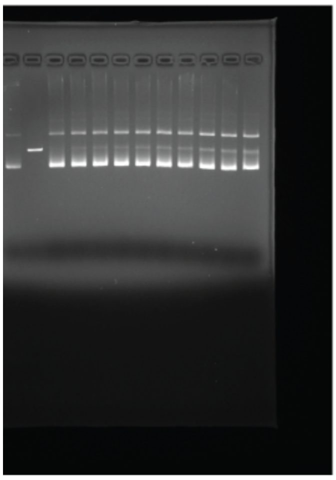

FIG 3B DNM-2 S83L

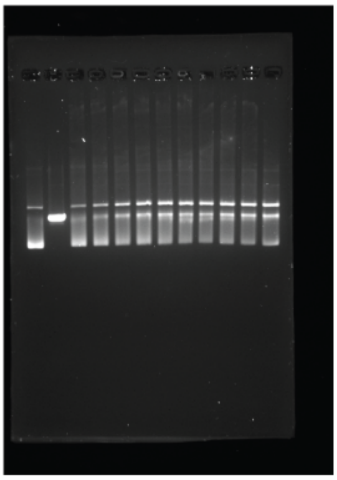

FIG 3B DNM-2 S83R

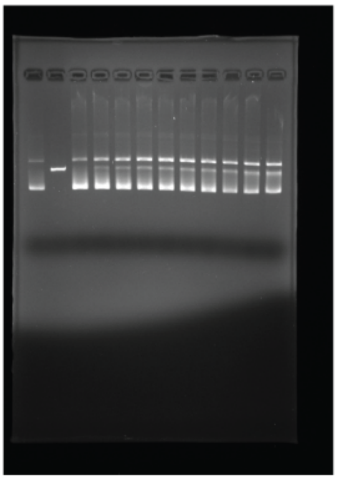

Supplementary Figure 3. Full gels from Figure 3B

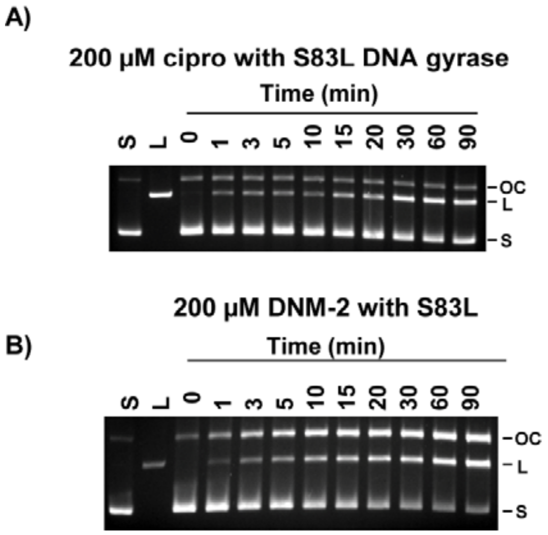

Supplementary Figure 4. Cleavage assay time course with increased concentrations of CIP and DNM-2. *(A)* Time course of DNA cleavage of 200 µM CIP with S83L DNA gyrase. Time points were 0, 1, 3, 5, 10, 15, 20, 30 ,60, and 90 min. *(B)* Time course of DNA cleavage of 200 µM DNM-2 with S83L DNA gyrase. Time points were 0, 1, 3, 5, 10, 15, 20, 30 ,60, and 90 min. Longer time points (120 and 180 min) were also investigated with little change being observed (data not shown). One representative gel shown out of at least three independent experiments.

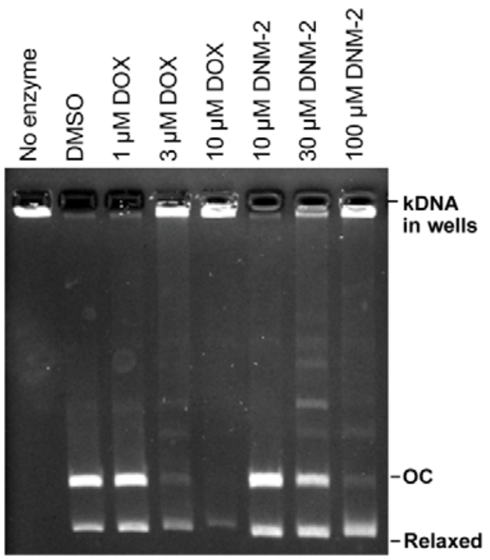

Supplementary Figure 5. Inhibition of human topoisomerase II. A decatenation assay was performed with human topoisomerase II in the presence of either DMSO, doxorubicin (DOX) or DNM-2 at the indicated concentrations. One representative gel shown out of at least three independent experiments.

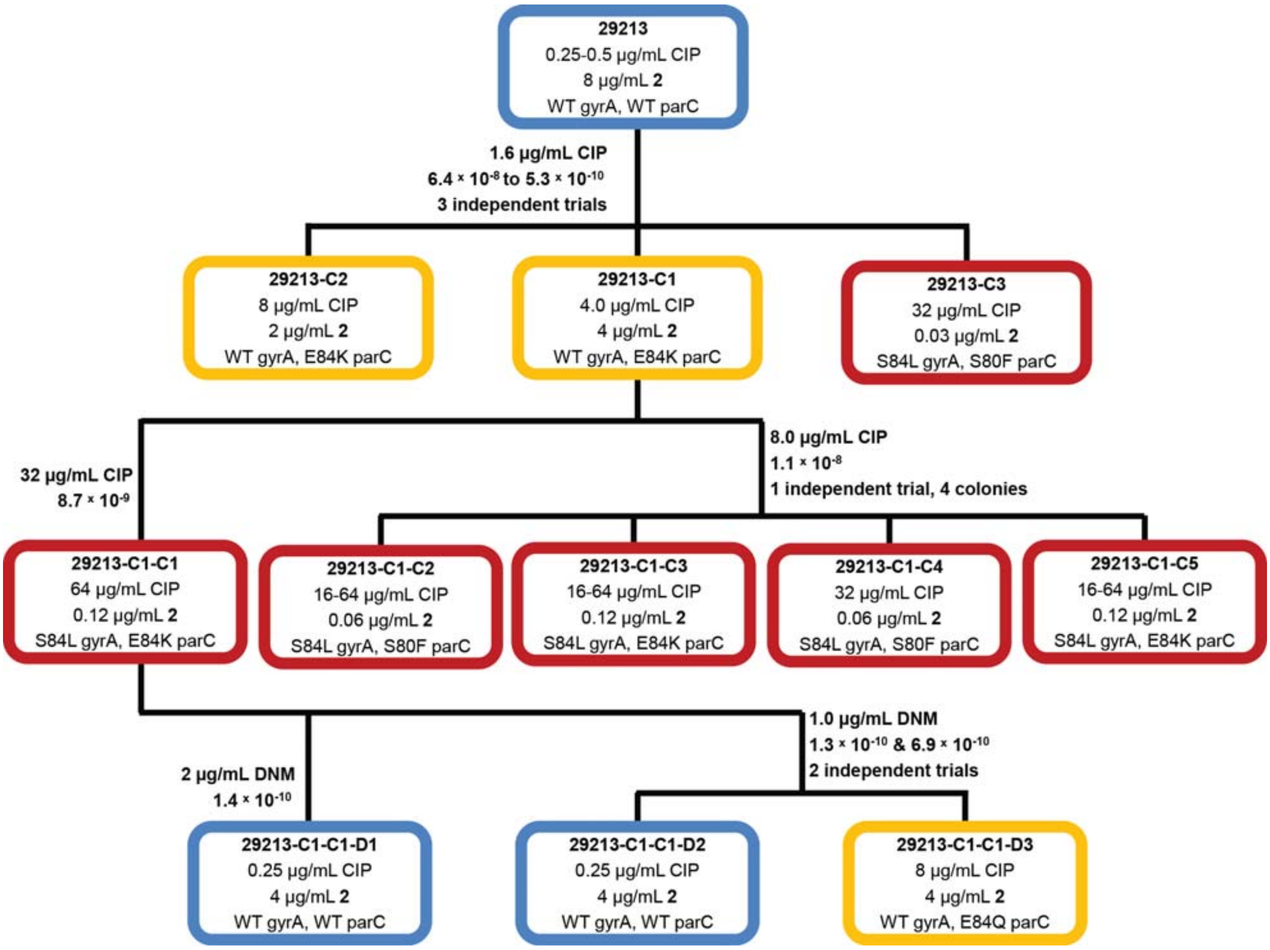

Supplementary Figure 6. Resistance cycling of CIP and DNM with *S. aureus* strain ATCC 29213. Conditions for selection along with resistance frequency are indicated above the boxes to which they correspond. In each box is the strain name, MIC for CIP, MIC for DNM-2, and the mutations in the QRDR of gyrA and parC. Blue = CIP sensitive (MIC < 4 µg mL^{-1}), Yellow = low level CIP resistance (16 > MIC ≥ 4 µg mL^{-1}), Red = high level CIP resistance (MIC ≥ 16 µg mL^{-1}).

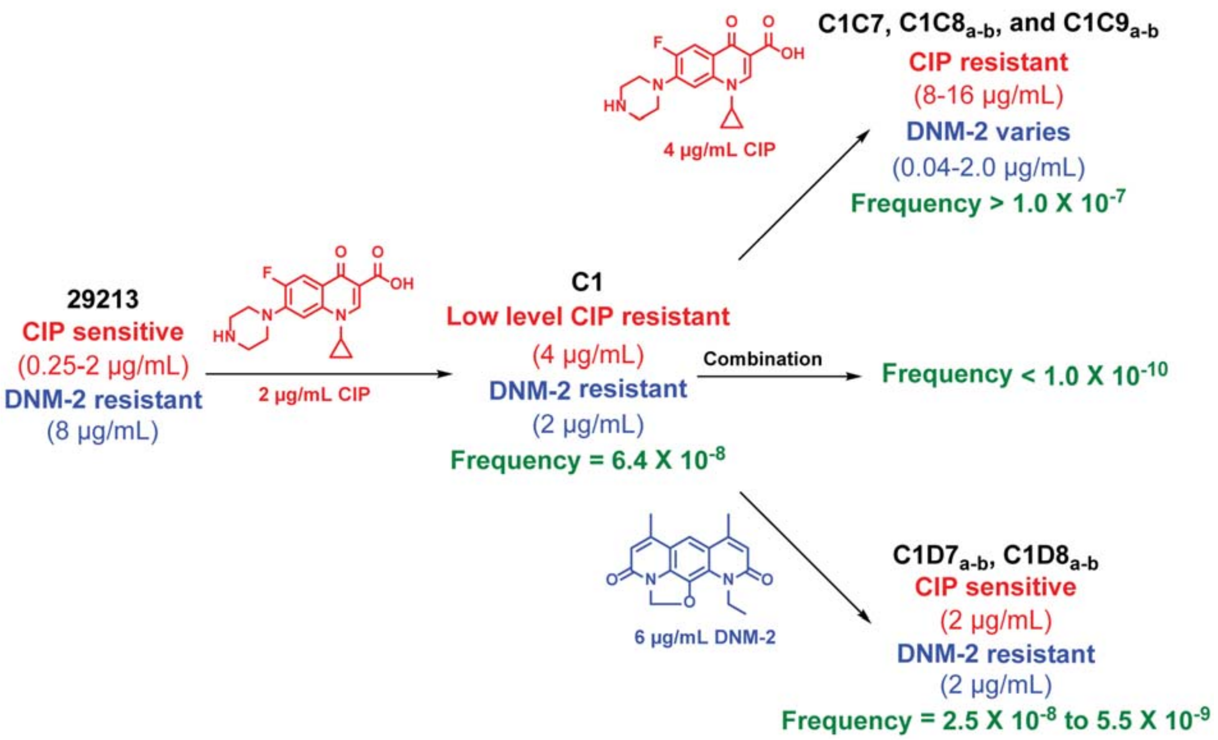

Supplementary Figure 7. Development of co-resistance to CIP and DNM-2 with *S. aureus* strain ATCC 29213. Initially, a low level CIP resistant mutant (C1) was generated. This strain was then treated with CIP alone, DNM-2 alone, or a combination. Along each arrow is indicated the conditions used to select for resistance. Below each strain (black) is listed the CIP MIC (red), DNM-2 MIC (blue), and the frequency of the mutation observed (green)

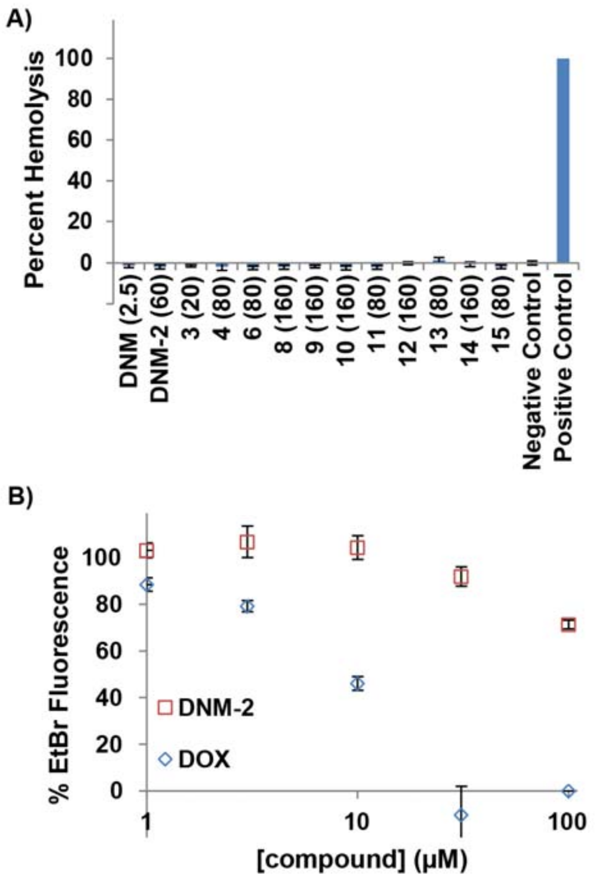

Supplementary Figure 8. In vitro toxicity. A) Hemolysis assay. Human red blood cells were co-incubated with compound. After incubation for 2 h at 37°C, the supernatant was analyzed for hemolysis. Each compound was tested either at 160 μg mL^{-1} or at the highest concentration which its solubility allowed. Concentrations (μg mL^{-1}) are indicated in parenthesis. The negative control is DMSO and the positive control is water. Data shown is from three independent replicates ± SEM. B) Ethidium bromide (EtBr) intercalation assay. Compounds were incubated with Herring Sperm DNA, ethidium bromide, and compound of interest for 30 minutes. The solutions were then analyzed for ethidium bromide fluorescence. Any decrease in percentage fluorescence is indicative of compound intercalation. Doxorubicin (DOX) was used as a positive control.

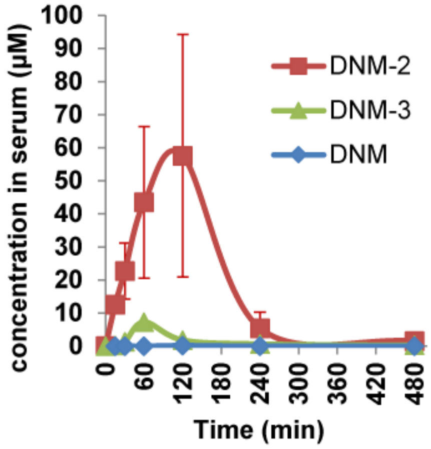

Supplementary Figure 9. Pharmacokinetic analysis of DNM, DNM-2, and DNM-3. C57/BL6 mice were treated with 50 mg kg^{-1} DNM, DNM-2, or DNM-3 via oral gavage. After the indicated time points (15, 30, 60, 120, 240, and 480 min), mice were sacrificed and the serum concentrations of DNM, DNM-2, and DNM-3 was determined by HPLC.

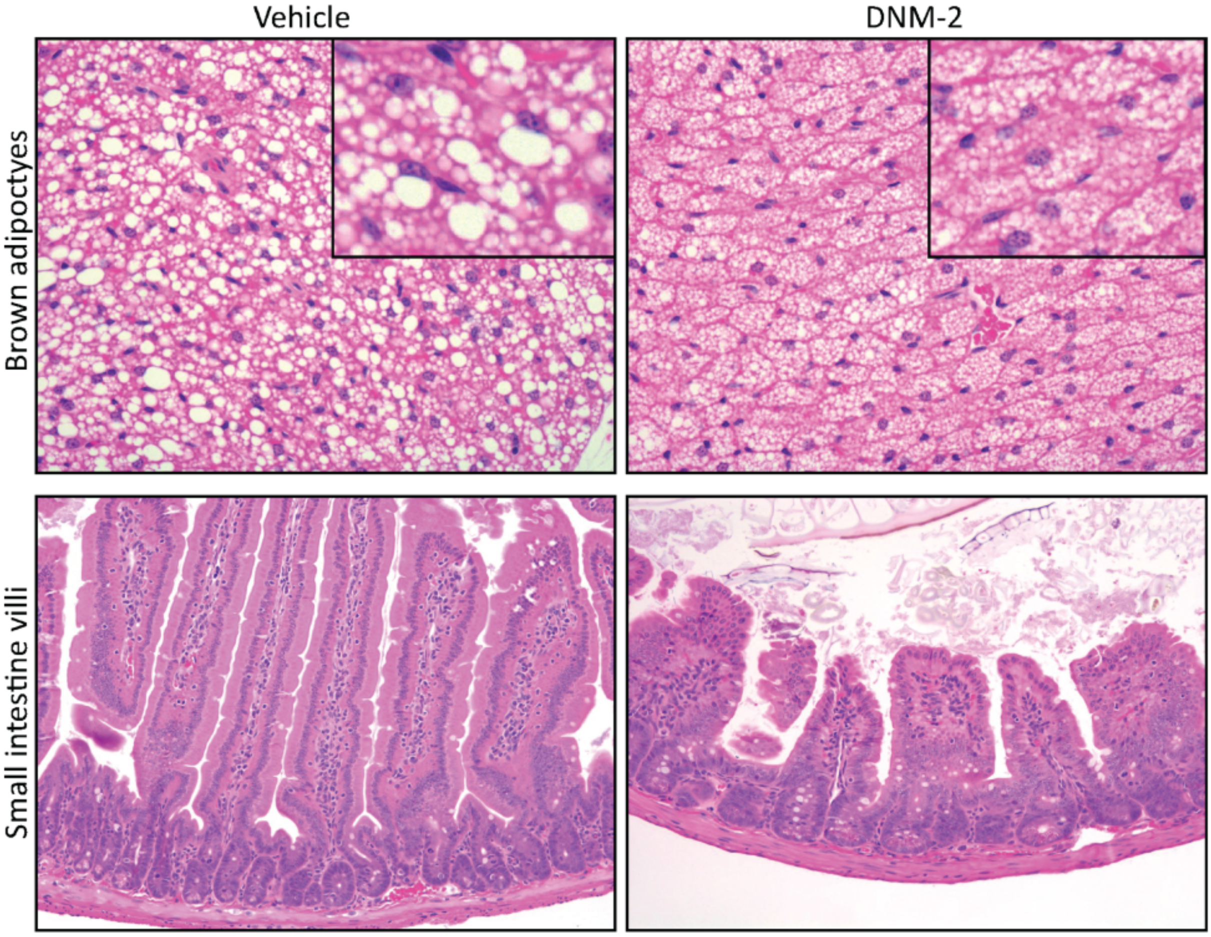

Supplementary Figure 10. In vivo toxicity studies. After euthanasia, mouse organs were collected for histopathological analyses. Tissue sections were stained with hematoxylin and eosin. All slides were systematically evaluated for evidence of acute or chronic inflammation and toxicity. No long-term pathologic effects were noted in kidneys, brains, lungs, livers, spleens, hearts, and stomachs. In small intestine sections, mild intestinal dilation associated with villi atrophy was noted. Also noted was increased vacuolation of white and brown adipocytes. These changes were considered of minimal significance.

Supplementary Table 1: Solubility and Activity of DNM and its derivatives

CIP	DAPT	VANC	LINEZ	DNM	2	3	4	5	6	7	8	9	10	11	12	13	14	15
**Solubility in pH 7.4 PBS (μM)**																		
				9 ± 3	121 ± 4	27 ± 1	53 ± 8	ND	ND	ND	68 ± 9	ND	ND	ND	ND	48 ± 8	<1	ND
**Solubility in DMSO (mM)**																		
				0.2	4	1.3	4.7	3.4	6.4	5.8	11.3	26.9	56.7	7.1	38.7	5.2	18.3	4.2
**MIC against *S. aureus* 29213 (μg mL^{-1}) WT GyrA and WT ParC**																		
0.25-0.5	2	1	2	>1	8	8	8	4	16	16	16	32	>64	>32	32	>32	>32	>32
**MIC against *S. aureus* NRS3 (μg mL^{-1}) S84L GyrA and S80F ParC**																		
>64	8	8	0.5	0.03	0.03	0.12	0.25	0.03	0.25	1	0.12	0.25	1	2	16	16	32	32

The solubility in PBS (pH 7.4) was determined by weighing a small amount of compound (0.5 – 2.0 mg) into a 1.7 mL Eppendorf tube. Enough PBS was added to make a 1 mg/mL solution. Compounds were then assessed by LC-MS and compared to a calibration curve to determine the solubility. More details can be found in the Supplementary Materials and Methods. Data shown is from three independent replicates ± SEM. ND = not determined. DMSO solubility of compounds was determined by weighing a small amount of compound (typically 1-2 mg) into a glass vial and adding DMSO dropwise until the compound was fully dissolved. Between DMSO additions, the vial was vortexed and sonicated. MICs with ciprofloxacin (CIP), daptomycin (DAPT), vancomycin (VANC), linezolid (LINEZ), deoxynybomycin (DNM), and DNM derivatives were determined using the microdilution broth method as outlined by the Clinical and Laboratory Standards Institute.

Supplementary Table 2: Activity of DNM and its derivatives against Gram-negative bacteria							
Strain	CIP (S/R)	CIP MIC (μg mL^{-1})	DNM MIC (μg mL^{-1})	DNM-2 MIC (μg mL^{-1})	DNM-8 MIC (μg mL^{-1})	gyrA mutation	parC mutation
E. coli ATCC 25922	S	0.025	>4	>24	>32	none	none
E. coli MG1655	S	0.025	ND	>24	ND	none	none
E. coli MG1655 ΔAcrB	S	0.008	ND	2	ND	none	none
P. aeruginosa PAO1	S	0.5	>4	>24	>32	none	none
PA1000	R*	ND	>4	>24	>32	ND	ND
PA1586	R*	ND	>4	>24	>32	ND	ND
A. baumannii ATCC 19606	S	1	>4	>24	>32	none	none
A. baumannii KB349	R*	>32*	>4	>24	>32	S83L	none
A. baumannii KB304	R*	24*	>4	>24	>32	S83L	none

The ciprofloxacin (CIP) sensitivity where a strain was considered sensitive (S) or resistant (R) based on the CLSI guidelines for each bacteria. MICs with CIP, DNM, DNM-2, and DNM-8 were determined using the microdilution broth method as outlined by the Clinical and Laboratory Standards Institute. *E. coli* MG1655 and the ΔAcrB strain were obtained from Prof. Cari Vanderpool at UIUC. *P.* aeruginosa clinical isolates were obtained from Cubist Pharmaceuticals (Lexington, MA).[1] *A. baumannii* clinical isolates were obtained from Dr. John Quale at the Division of Infectious Diseases at SUNY Downstate Medical Center.[2] ND = not determined. *These strains were previously determined to be CIP resistant [1-2].

Supplementary Table 3: Sensitivity of MRSA clinical isolates to CIP, DNM, DNM derivatives, and other antibiotics and tabulated data from Figure 2A										
Strain ID	CIP S/I/R	CIP MIC (μg mL^{-1})	DNM MIC (μg mL^{-1})	DNM-2 MIC (μg mL^{-1})	DNM-8 MIC (μg mL^{-1})	Vanc MIC (μg mL^{-1})	Amp MIC (μg mL^{-1})	Novo MIC (μg mL^{-1})	gyrA mutations	parC mutations
SAU.42	S	0.25	1	2	8	1	2	0.5	ND	ND
SAU.1118	S	0.5	1	2	8	2	>64	0.5	ND	ND
SAU.3017	S	1	1	4	4	1	>64	0.5	ND	ND
SAU.3021	R	8	0.031	0.063	0.125	1	>64	0.25	ND	ND
SAU.446	R	16	0.25	0.125	0.25	2	>64	0.5	ND	ND
SAU.491	R	16	0.125	0.125	0.5	2	>64	0.5	Ser84Leu	Ser80Phe
SAU.555	R	16	0.125	0.25	0.5	1	>64	0.25	ND	ND
SAU.493	R	32	0.063	0.125	0.25	1	>64	0.5	Ser84Leu	Ser80Phe
SAU.710	R	32	0.031	0.063	0.125	2	>64	0.5	Ser84Leu	Ser80Phe
SAU.3024	R	32	0.031	0.125	0.125	2	>64	0.5	ND	ND
SAU.3026	R	32	0.125	0.125	0.25	1	>64	0.5	ND	ND
SAU.419	R	64	0.125	0.063	0.25	4	>64	0.25	ND	ND
SAU.846	R	64	0.031	0.25	0.5	8	>64	0.125	ND	ND
SAU.2996	R	64	0.25	0.5	2	1	>64	0.5	ND	ND
SAU.447	R	>64	0.063	0.125	1	2	>64	0.25	ND	ND
SAU.489	R	>64	0.031	0.125	0.25	2	>64	0.25	Ser84Leu	Ser80Tyr
SAU.492	R	>64	0.063	0.125	ND	1	>64	0.5	Ser84Leu	Ser80Tyr
SAU.494	R	>64	0.063	0.125	0.25	1	>64	0.125	Ser84Leu	Ser80Phe
SAU.495	R	>64	0.063	0.125	0.25	2	>64	0.5	Ser84Leu	Ser80Phe
SAU.496	R	>64	0.063	0.125	0.25	1	>64	0.5	Ser84Leu	Ser80Phe
SAU.669	R	>64	0.031	0.063	0.125	2	>64	0.5	ND	ND
SAU.708	R	>64	0.063	0.125	1	2	64	0.25	Ser84Leu	Ser80Phe Glu84Lys
SAU.709	R	>64	0.063	0.125	0.25	1	>64	0.5	Ser84Leu	Ser80Tyr Glu84Gly
SAU.3025	R	>64	0.031	0.063	0.125	1	64	0.25	ND	ND

The ciprofloxacin (CIP) sensitivity where a strain was considered sensitive (S) if it had an MIC ≤ 4 μg mL^{-1}, intermediate (I) with a 16 > MIC > 4 μg mL^{-1}, or resistant (R) with a MIC ≥ 16 μg mL^{-1}. MICs with CIP, DNM, DNM-2, DNM-8, Vancomycin (Vanc), Ampicillin (Amp), and Novobiocin (Novo) were determined using the microdilution broth method as outlined by the Clinical and Laboratory Standards Institute. QRDR mutations were determined as described in the text using primers (primer sequences can be found in Supplementary Table 7). Clinical isolates were obtained from Cubist Pharmaceuticals (Lexington, MA). ND = not determined. CIP resistant strains are graphed in Figure 2A.

Supplementary Table 4: Sensitivity of ATCC strains and VRE clinical isolates to CIP, DNM, and DNM derivatives and tabulated data from Figure 2B

Strain	Species	CIP (S/I/R)	Cipro MIC (μg mL^{-1})	DNM MIC (μg mL^{-1})	DNM-2 MIC (μg mL^{-1})	DNM-8 MIC (μg mL^{-1})	gyrA mutations	parC mutations
ATCC 29212	*E. faecalis*	S	0.5-2	>1	8	8	WT	WT
ATCC 19433	*E. faecalis*	S	2	>1	8	16	WT	WT
S235	*E. faecium*	R	>64	0.125	0.5	1	Ser83Ile	Ser80Ile
S51	*E. faecium*	R	>64	0.25	0.5	1	Ser83Ile	Ser80Ile
S122	*E. faecium*	R	64	0.25	0.5	1	Ser83Ile	Ser80Ile
S226	*E. faecium*	R	64	0.25	0.5	1	Ser83Ile	Ser80Ile
S344	*E. faecium*	R	>64	0.25	0.5	1	Ser83Ile	Ser80Ile
S234	*E. faecalis*	R	32	0.25	0.25	0.5	Ser83Ile	Ser80Ile Glu84Asp
S557	*E. faecium*	R	>64	0.25	0.25	0.5	Ser83Ile	Ser80Ile
C27569	*E. faecium*	R	>64	0.25	0.25	1	Ser83Ile	Ser80Ile
C28535	*E. faecalis*	R	32	0.25	0.25	1	Ser83Ile	Ser80Ile
D1	*E. faecium*	R	>64	0.25	0.5	0.5	Ser83Ile	Ser80Ile
C21667	*E. faecalis*	R	>64	0.25	0.5	1	Ser83Ile	Ser80Ile
C28036	*Ent. Spp.*	R	64	0.25	0.5	1	Ser83Ile	Ser80Ile
SL152	*E. faecium*	R	>64	0.25	0.5	1	Ser83Ile	Ser80Ile
S206	*E. faecalis*	R	64	1	2	4	Ser83Arg	Ser80Ile
U63	*E. faecium*	R	>64	1	4	4	Ser83Arg	Ser80Ile
U275	*E. faecium*	R	>64	1	2	4	Ser83Cys Glu87Gly	Ser80Ile
U464	*E. faecium*	R	>64	1	2	2	Ser83Arg	Ser80Ile
S34	*E. faecium*	R	64	1	1	2	Ser83Ile	Ser80Arg
C27282	*E. faecium*	R	64	>1	4	8	Ser83Arg	Ser80Arg
U503	*E. faecium*	R	>64	>1	4	4	Ser83Arg	Ser80Ile
U563	*E. faecium*	R	>64	>1	2	4	Ser83Arg	Ser80Ile
C21190	*E. faecium*	R	>64	>1	8	8	Ser83Arg	Ser80Ile

The ciprofloxacin (CIP) sensitivity where a strain was considered sensitive (S) if it had an MIC ≤ 4 μg mL^{-1}, intermediate (I) with a 16 > MIC > 4 μg mL^{-1}, or resistant (R) with a MIC ≥ 16 μg mL^{-1}. MICs with CIP, DNM, DNM-2, and DNM-8 were determined using the microdilution broth method as outlined by the Clinical and Laboratory Standards Institute CLSI. QRDR mutations were determined as described in the text using primers (primer sequences can be found in Supplementary Table 7). CIP resistant strains are graphed in Figure 2B.

Supplementary Table 5: Pharmacokinetic parameters for DNM, DNM-2, and DNM-3				
	$t_{1/2}$ (hr)	AUC (hr ng mL^{-1})	C_{max} (ng mL^{-1})	T_{max} (hr)
DNM			< 60	
DNM-2	0.9	44000	12800	1.2
DNM-3	1.2	4000	1260	1.1

Pharmacokinetic parameters were determined from curves presented in Supplementary Figure 9.

Supplementary Table 6: Hematologic Toxicity of DNM-2			
	**Vehicle**	**DNM-2 (50 mg kg^{-1})**	**Normal values[3] (Range)**
**RBC ($X10^6/\mu L$)**	7.35 ± 0.2*	8.5 ± 0.2*	9.07 ± 0.49 (7.77 - 9.77)
**Hemoglobin (g/dL)**	12.8 ± 0.5	14.2 ± 0.3	13.4 ± 0.616 (12.0 - 14.5)
**Hematocrit (%)**	38.3 ± 1	40 ± 1	44.9 ± 2.09 (39.8 - 48.6)
**Platelet (cells/µL)**	330,000 ± 162,000*	60,000 ± 10,000*	1,310,000 ± 188,000 (990,000 - 1,840,000)
**Mean Cell Volume (fl)**	52.1 ± 0.6	47.3 ± 0.4	50 ± 0.64 (49 - 51)
**WBC (cells/µL)**	4970 ± 1240	3800 ± 300	5800 ± 810 (4400 - 7200)
**Neutrophil (% of WBC)**	11.2 ± 4.5	18 ± 3	14 ± 7.9 (2.0 - 30)
**Lymphocyte (% of WBC)**	85.2 ± 3	81 ± 3	81 ± 8.7 (60 - 98)
**ALT (U/L)**	32 ± 4	36 ± 1	39 ± 7.9 (28 - 57)
**ALP (U/L)**	160 ± 50	60 ± 10	72 ± 13 (40 - 90)
**Albumin (g/dL)**	2.2 ± 0.2	3.2 ± 0.1	2.7 ± 0.23 (2.4 - 3.0)
**Globulin (g/dL)**	2.7 ± 0.1	2.62 ± 0.05	1.2 ± 0.25 (0.8 - 1.5)
**Total Bilirubin (mg/dL)**	0.43 ± 0.07	0.7 ± 0.1	0.2 ± 0.04
**BUN (Urea, mg/dL)**	29 ± 1	32.0 ± 0.4	29 ± 4.9 (24 - 40)
**Creatine (mg/dL)**	0.17 ± 0.03	0.2 ± 0.0	0.6 ± 0.2 (0.4 - 1.0)

Hematologic toxicity of DNM-2. No clinically significant evidence for myelosuppression, renal injury, or hepatic toxicity was identified in any of the treatment groups. *Platelet cell counts were low because many platelet clumps were observed. This was reflected in lower RBC counts. Total bilirubin increases were observed for both vehicle and DNM-2-treated mice due to hemolysis during blood collection. Normal values were reported by Schnell and Wilson [3].

Supplementary Table 7: Primers used for sequencing and for site directed mutagenesis		
Primer	Sequence	Reference
Enterococcus faecalis_gyrA_F	5'-ATGAGTGAAGAAATTAAAGAAAACATTCA-3'	Sato, Kenichi et. al. *Antimicrob. Agents Chemother.* **2002**, *46* , 1800-1804
Enterococcus faecalis_gyrA_R	5'-ACTCATACGTGCTTCGGTATAACGC-3'	Sato, Kenichi et. al. *Antimicrob. Agents Chemother.* **2002**, *46* , 1800-1804
Enterococcus faecalis_parC_F	5'-GTGACAATTTTGGAAAAACGCCAAG-3'	Sato, Kenichi et. al. *Antimicrob. Agents Chemother.* **2002**, *46* , 1800-1804
Enterococcus faecalis_parC_R	5'-CACCACTTAACTGTGATAAACGAGC-3'	Sato, Kenichi et. al. *Antimicrob. Agents Chemother.* **2002**, *46* , 1800-1804
Enterococcus faecium_gyrA_F	5'-CGGGATGAACGAATTGGGTGTGA-3"	Torres et. al. *J. Chemotherapy.* **2011**, *23* , 87-91
Enterococcus faecium_gyrA_R	5'-AATTTTACTCATACGTGCTTCGG-3'	Torres et. al. *J. Chemotherapy.* **2011**, *23* , 87-91
Enterococcus faecium_parC_F	5'-TTCCCGTGCATTTCGATCAGTACTTC-3'	Torres et. al. *J. Chemotherapy.* **2011**, *23* , 87-91
Enterococcus faecium_parC_R	5'-CGTATGACAAAGGATTCGGTAAATC-3'	Torres et. al. *J. Chemotherapy.* **2011**, *23* , 87-91
Saureus_gyrA_F	5'-GGATTAAATGAACAAGGTATGACACCG-3'	Hiramatsu et. al. *Int. J. Antimicrob. Agents* **2012**, *39* , 478-485
Saureus_gyrA_R	5'-TAGTCATACGCGCTTCAGTATAACG-3'	Hiramatsu et. al. *Int. J. Antimicrob. Agents* **2012**, *39* , 478-485
Saureus_parC_F	5'-TTAGGTGATCGCTTTGGAAGATATAG-3'	Hiramatsu et. al. *Int. J. Antimicrob. Agents* **2012**, *39* , 478-485
Saureus_parC_R	5'-TACCATTGGTTCGAGTGTCG-3'	Hiramatsu et. al. *Int. J. Antimicrob. Agents* **2012**, *39* , 478-485
E coli S83L sense	5'-AATACCATCCCCATGGTGACTTGGCGGTCTATG-3'	Agilent QuikChange Primer Design
E coli S83L antisense	5'-CATAGACCGCCAAGTCACCATGGGGATGGTATT-3'	Agilent QuikChange Primer Design
E coli S83R sense	5'-CATCCCCATGGTGACAGGGCGGTCTATGACAC-3'	Agilent QuikChange Primer Design
E coli S83R antisense	5'-GTGTCATAGACCGCCCTGTCACCATGGGGATG-3'	Agilent QuikChange Primer Design

Supplementary Note 1: The NMR spectra

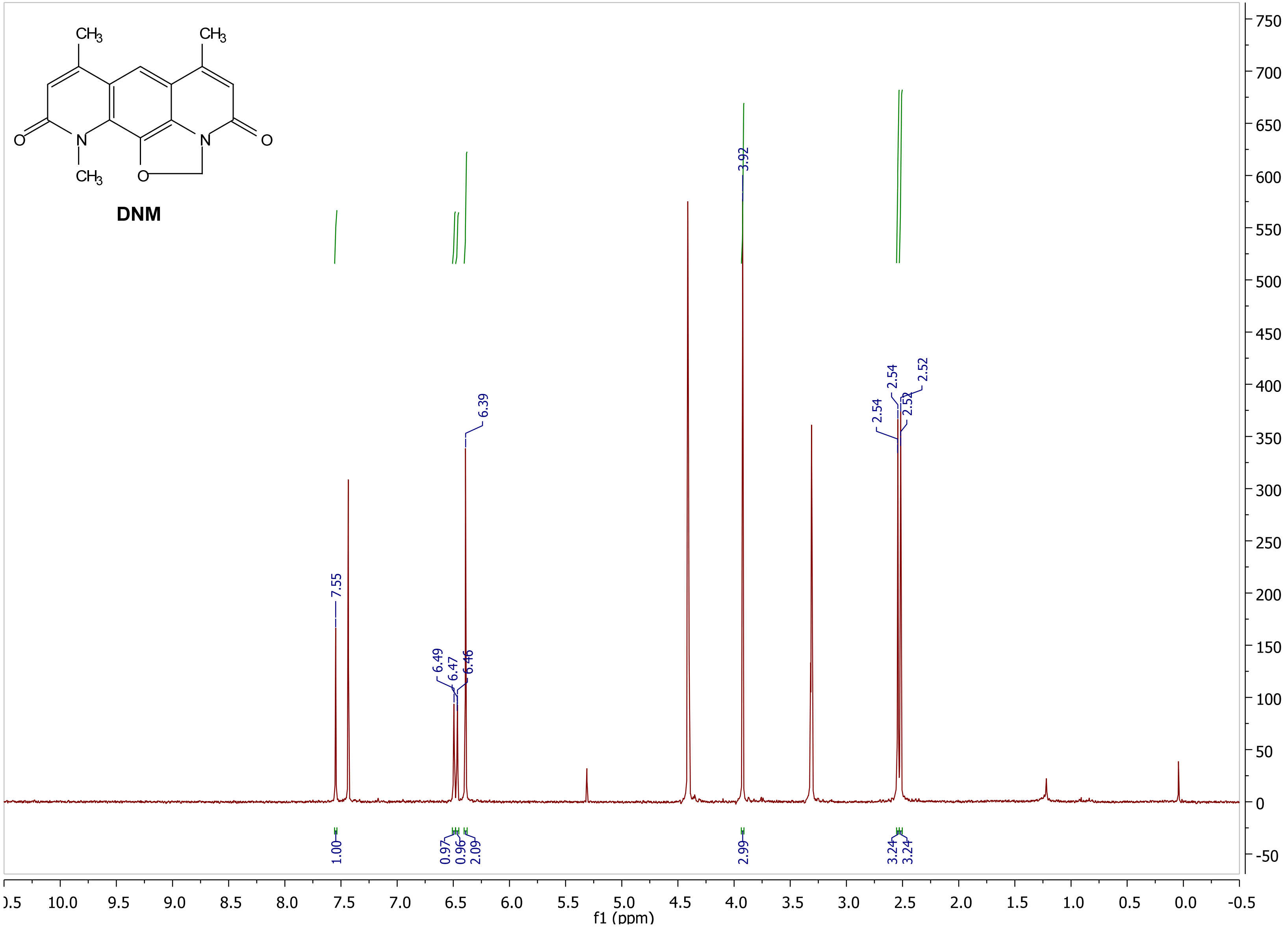

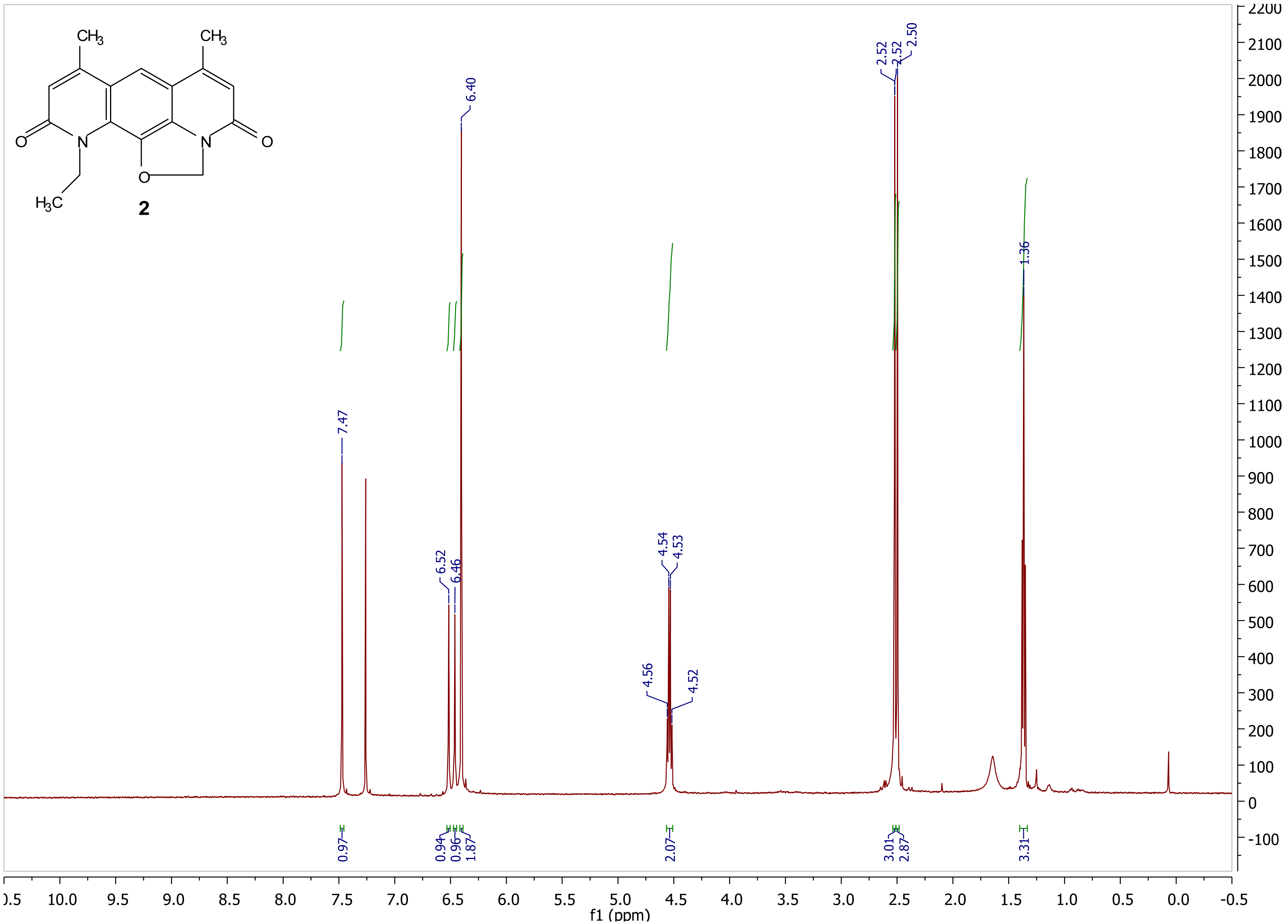

CH3
CH3
O
N
N
O
O
H3C
2
7.47
6.52
6.46
6.40
4.56
4.54
4.53
4.52
2.52
2.52
2.50
1.36
0.97
0.94
0.96
1.87
2.07
3.01
2.87
3.31
f1 (ppm)

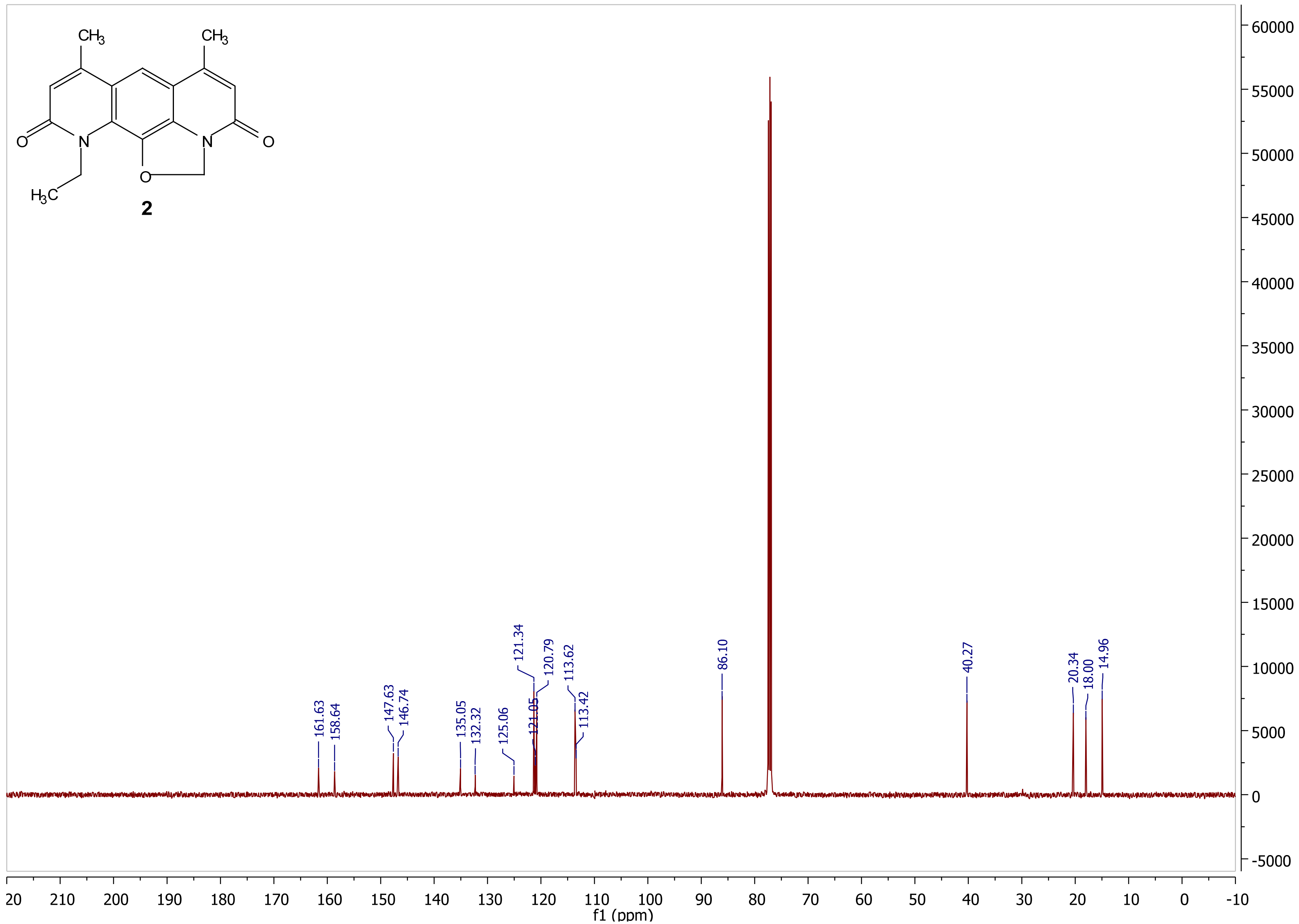
CH3
CH3
O
N
N
O
O
H3C
2
161.63
158.64
147.63
146.74
135.05
132.32
125.06
121.34
121.05
120.79
113.62
113.42
86.10
40.27
20.34
18.00
14.96
f1 (ppm)

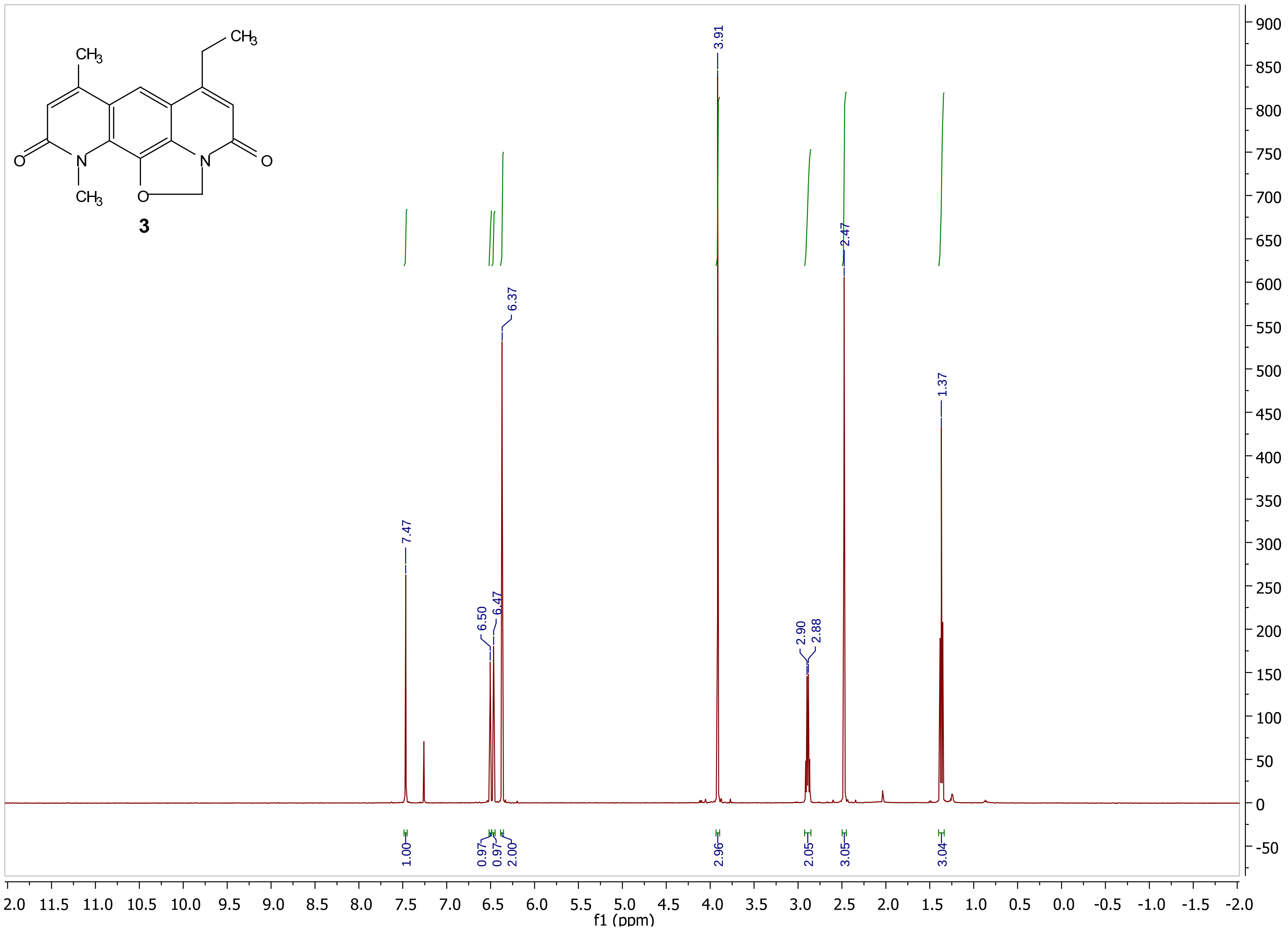

CH3
CH3
O
N
N
O
CH3
O
3
7.47
6.50
6.47
6.37
3.91
2.90
2.88
2.47
1.37
1.00
0.97
0.97
2.00
2.96
2.05
3.05
3.04
f1 (ppm)

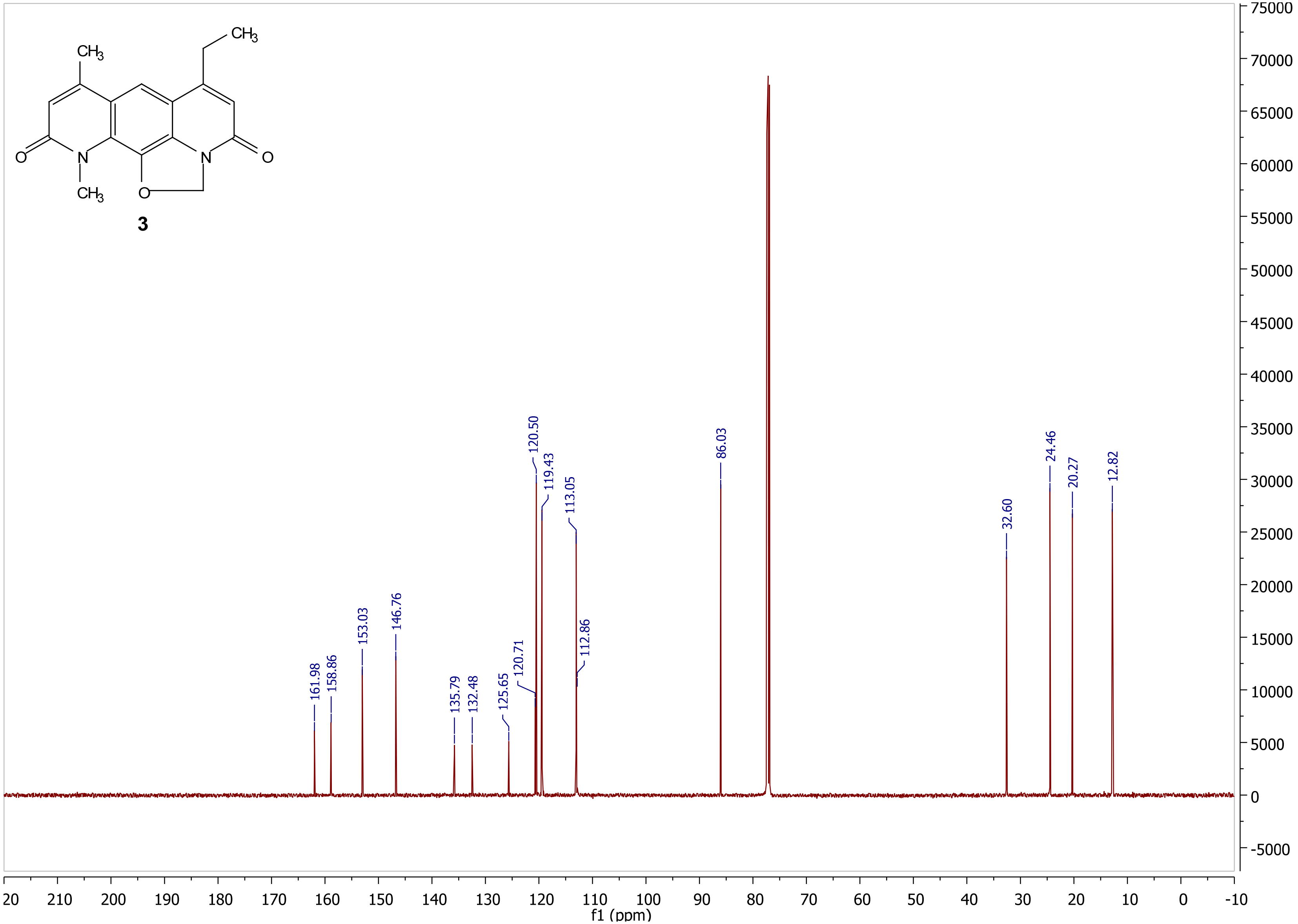

CH3
CH3
O
N
N
O
CH3
O
3
161.98
158.86
153.03
146.76
135.79
132.48
125.65
120.71
120.50
119.43
113.05
112.86
86.03
32.60
24.46
20.27
12.82
f1 (ppm)

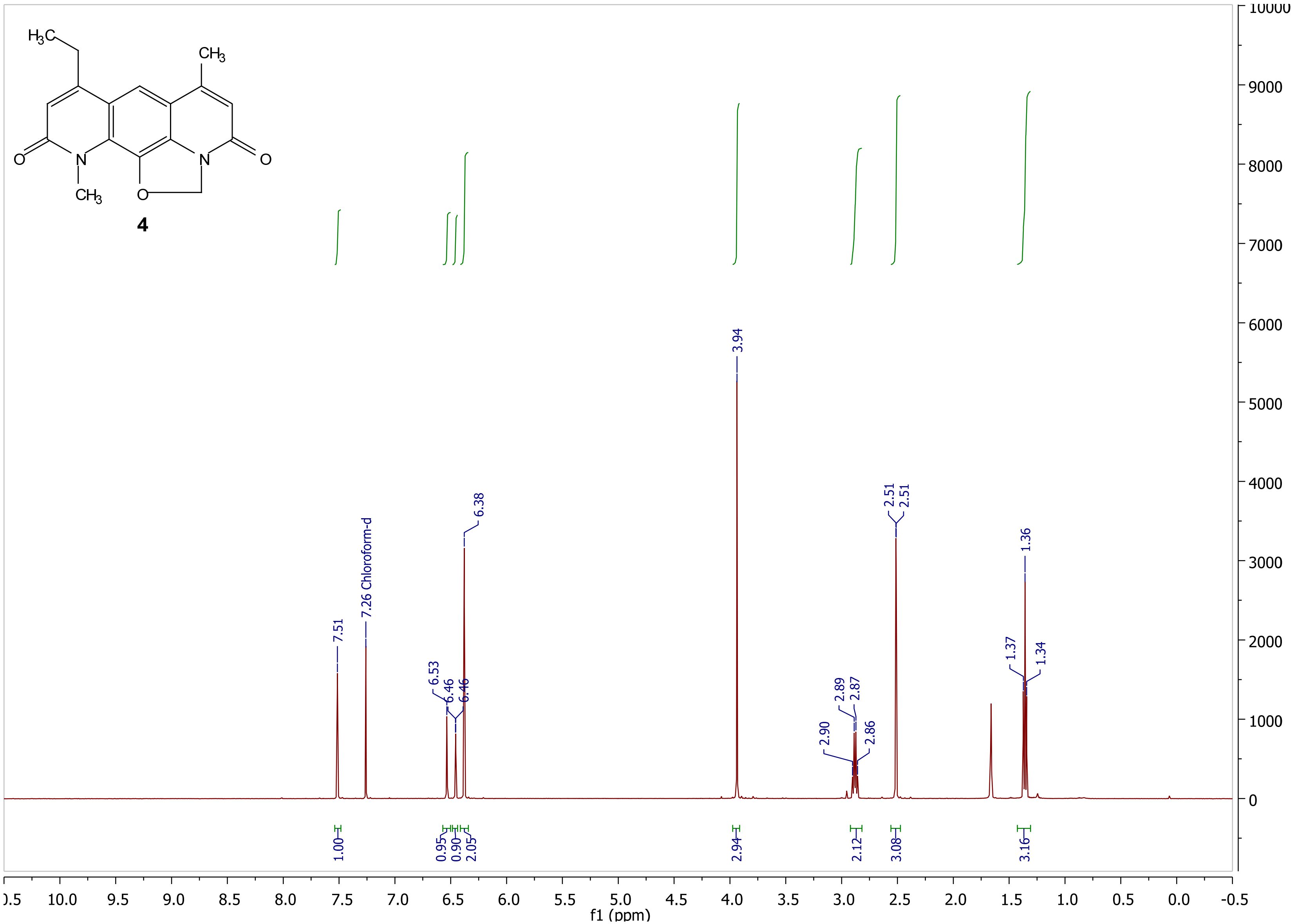
H3C
CH3
O
N
N
O
CH3
O
4
7.51
7.26 Chloroform-d
6.53
6.46
6.46
6.38
3.94
2.90
2.89
2.87
2.86
2.51
2.51
1.37
1.36
1.34
1.00
0.95
0.90
2.05
2.94
2.12
3.08
3.16
f1 (ppm)

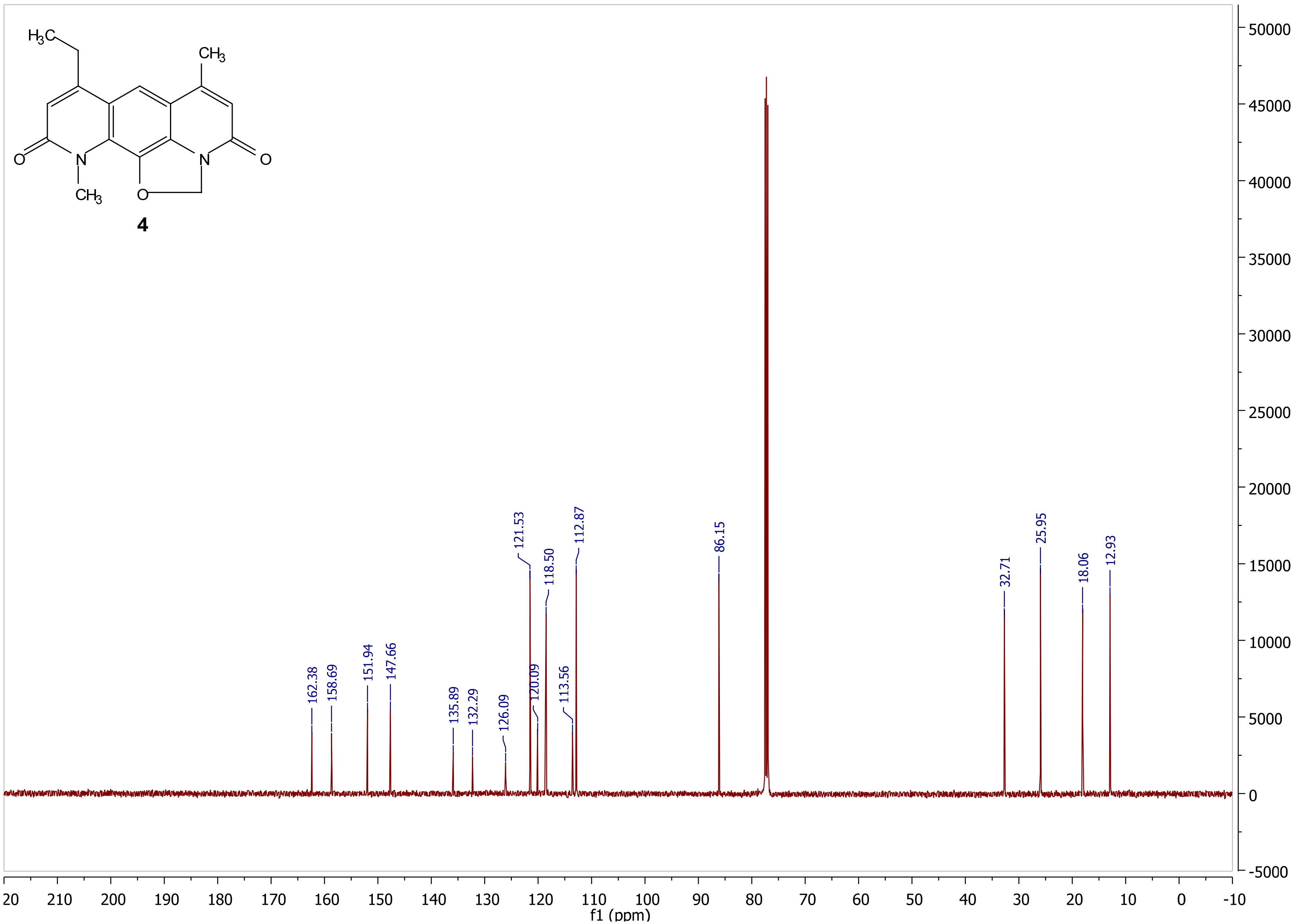
H3C
CH3
O
N
N
O
CH3
O
4
162.38
158.69
151.94
147.66
135.89
132.29
126.09
121.53
120.09
118.50
113.56
112.87
86.15
32.71
25.95
18.06
12.93
f1 (ppm)

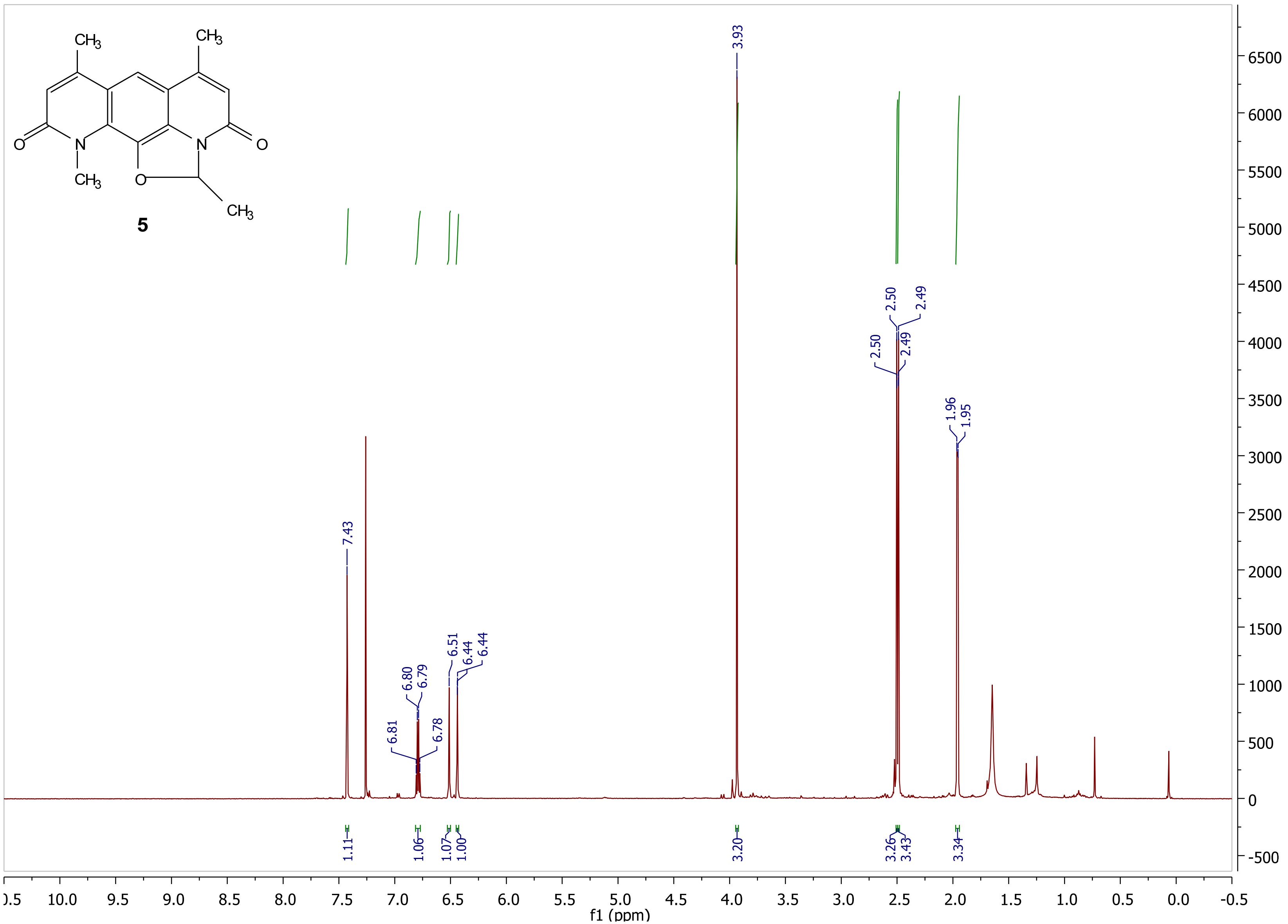
CH3
CH3
O
N
N
O
CH3
O
CH3
5
7.43
6.81
6.80
6.79
6.78
6.51
6.44
6.44
3.93
2.50
2.50
2.49
2.49
1.96
1.95
1.11
1.06
1.07
1.00
3.20
3.26
3.43
3.34
6500
6000
5500
5000
4500
4000
3500
3000
2500
2000
1500
1000
500
0
-500
10.0
9.5
9.0
8.5
8.0
7.5
7.0
6.5
6.0
5.5
5.0
4.5
4.0
3.5
3.0
2.5
2.0
1.5
1.0
0.5
0.0
-0.5
f1 (ppm)

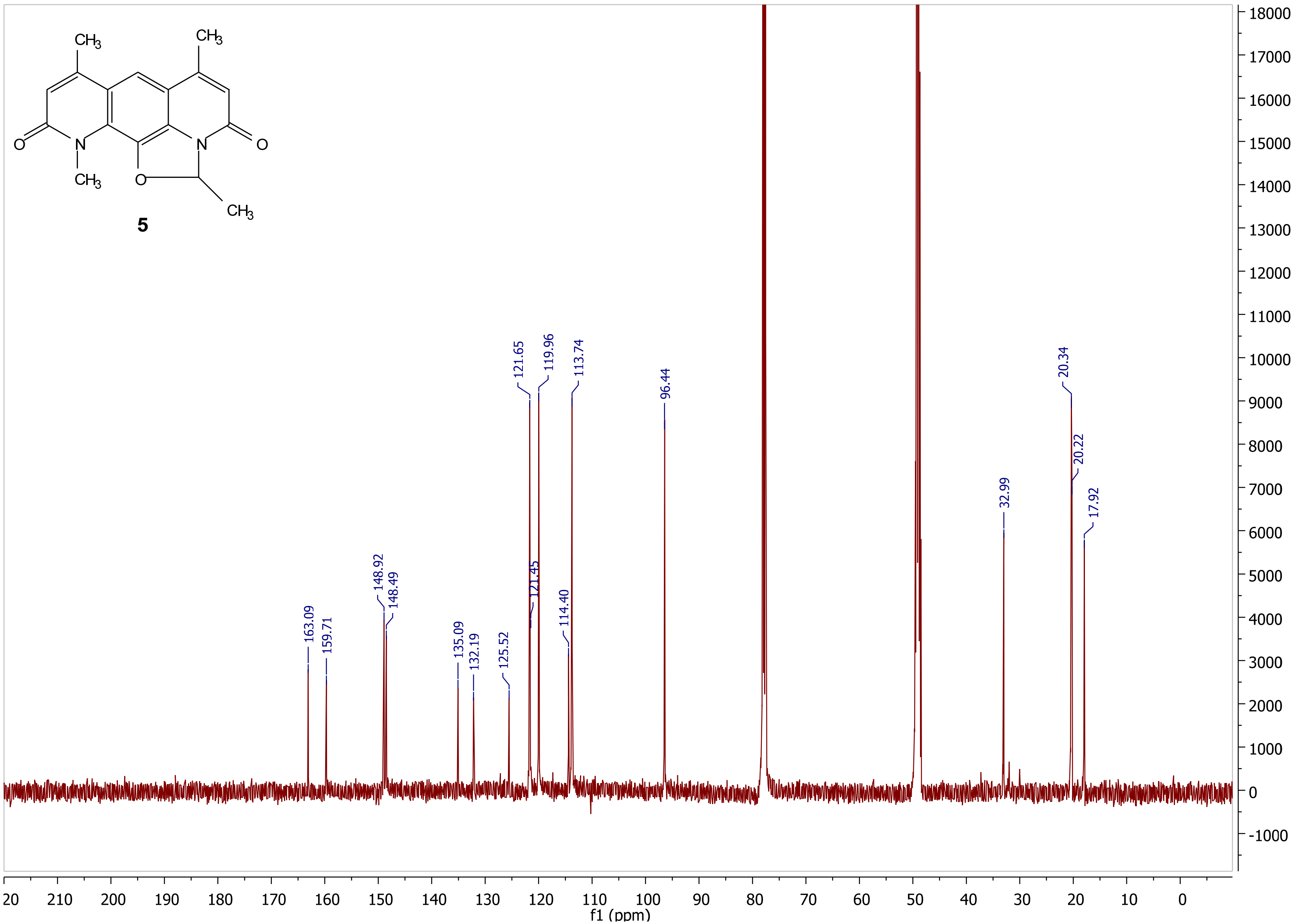
CH3
CH3
O
N
N
O
CH3
O
CH3
5
163.09
159.71
148.92
148.49
135.09
132.19
125.52
121.65
121.45
119.96
114.40
113.74
96.44
32.99
20.34
20.22
17.92
f1 (ppm)

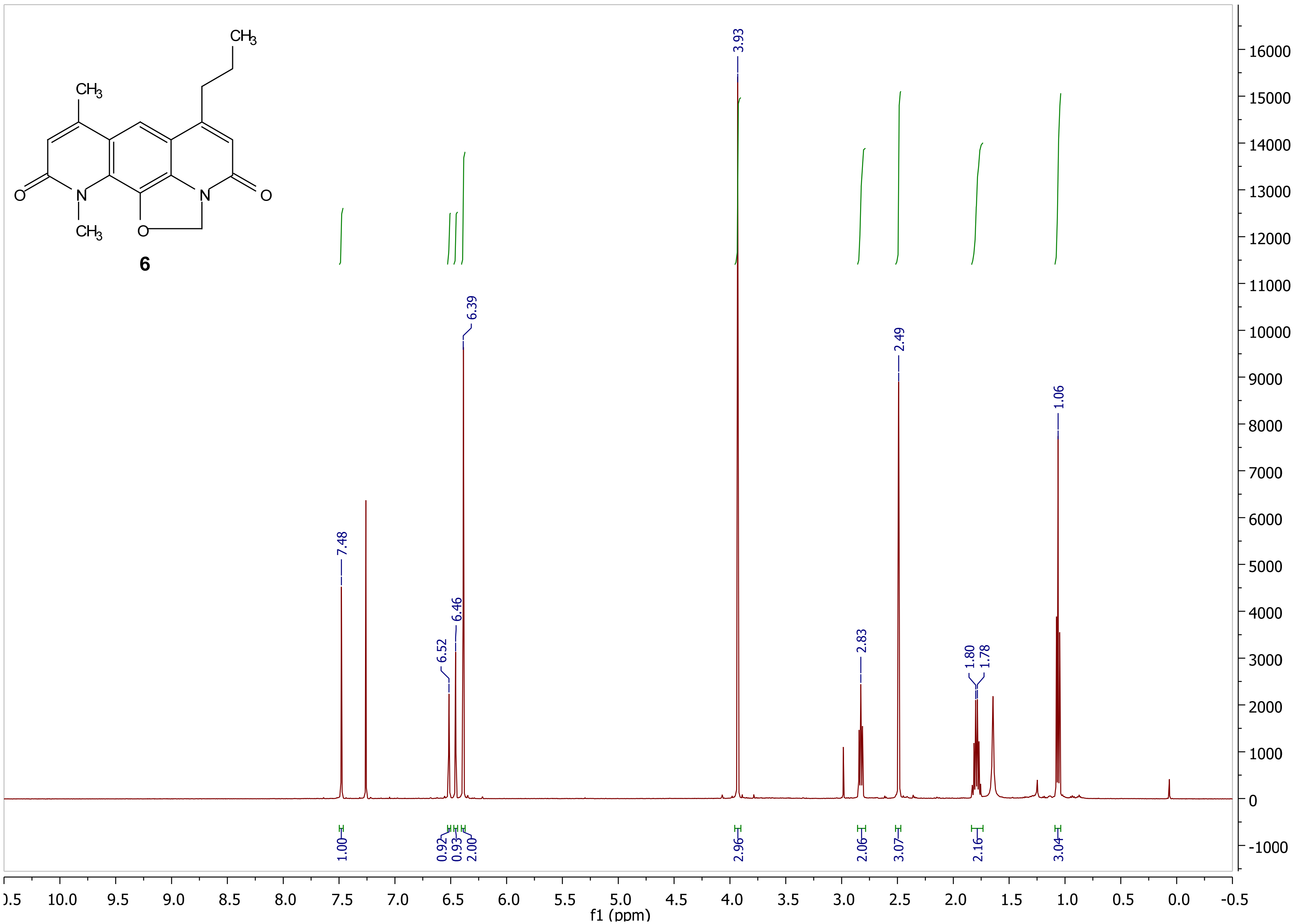

CH3
CH3
O
N
N
O
CH3
O
6
7.48
6.52
6.46
6.39
3.93
2.83
2.49
1.80
1.78
1.06
1.00
0.92
0.93
2.00
2.96
2.06
3.07
2.16
3.04
f1 (ppm)

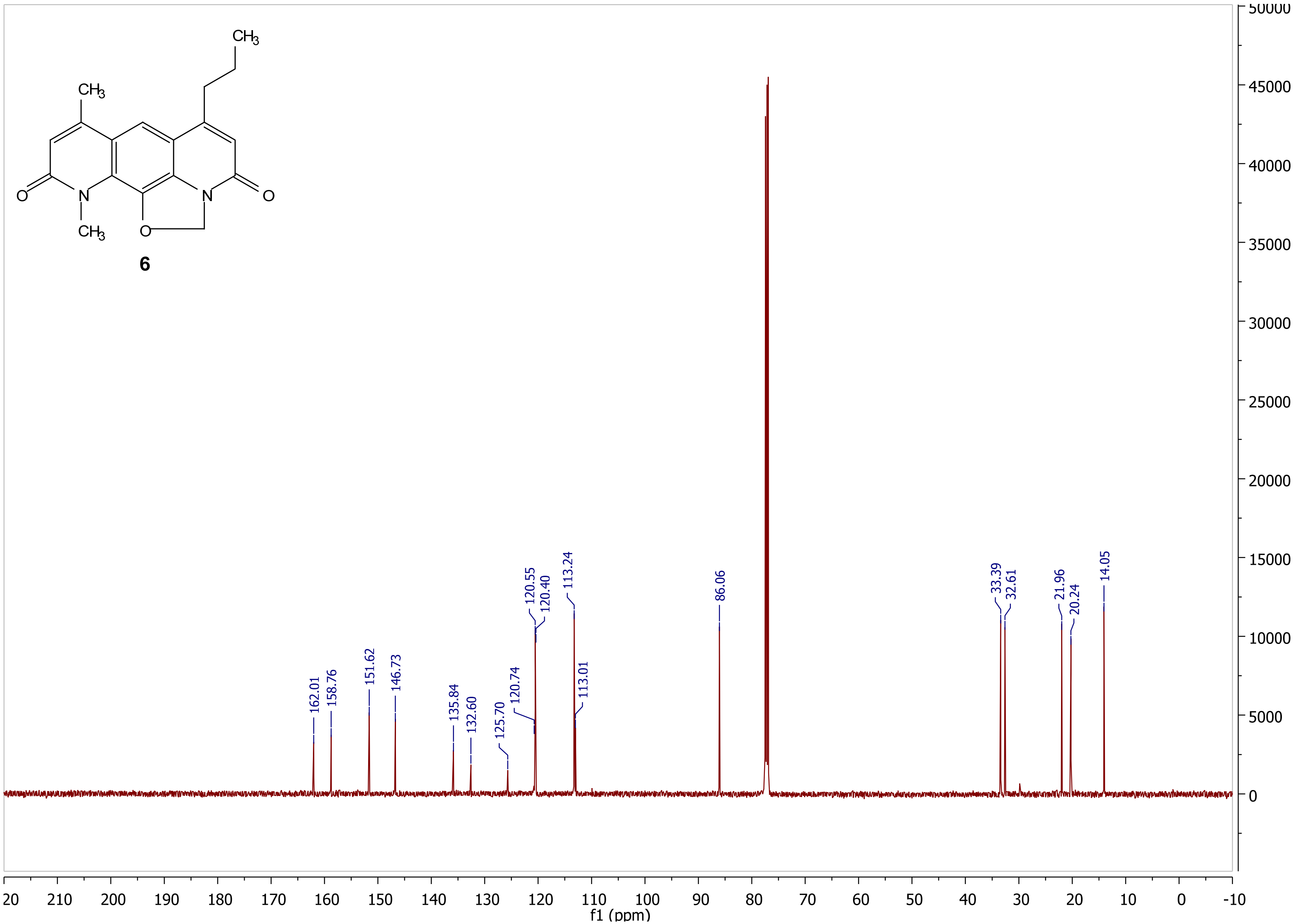
CH3
CH3
O
N
N
O
CH3
O
6
162.01
158.76
151.62
146.73
135.84
132.60
125.70
120.74
120.55
120.40
113.24
113.01
86.06
33.39
32.61
21.96
20.24
14.05
50000
45000
40000
35000
30000
25000
20000
15000
10000
5000
0
220 210 200 190 180 170 160 150 140 130 120 110 100 90 80 70 60 50 40 30 20 10 0 -10
f1 (ppm)

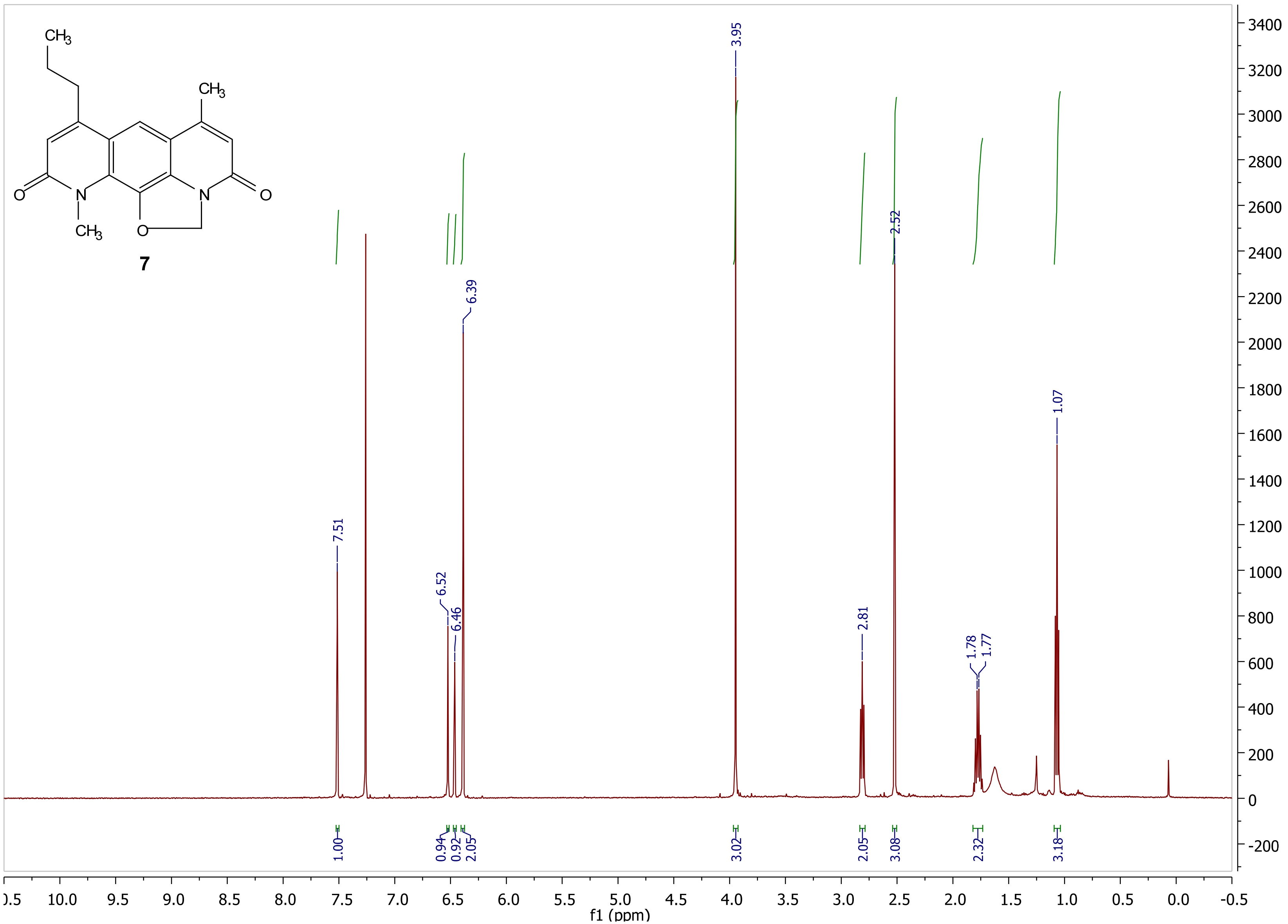
CH3
CH3
O
N
N
O
CH3
O
7.51
6.52
6.46
6.39
3.95
2.81
2.52
1.78
1.77
1.07
1.00
0.94
0.92
2.05
3.02
2.05
3.08
2.32
3.18
f1 (ppm)

7

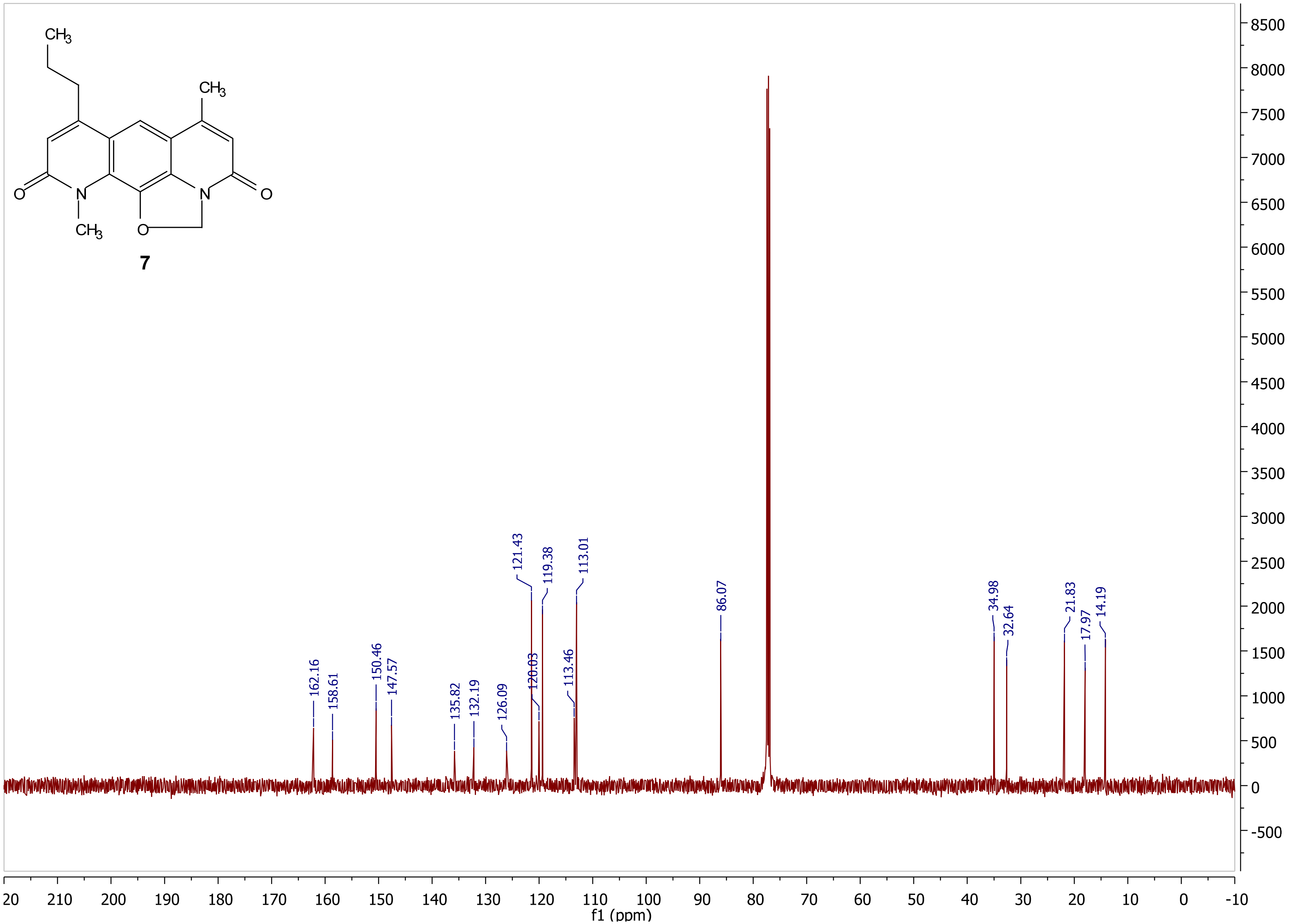
CH3
CH3
O
N
CH3
O
N
O
7
162.16
158.61
150.46
147.57
135.82
132.19
126.09
121.43
120.03
119.38
113.46
113.01
86.07
34.98
32.64
21.83
17.97
14.19
f1 (ppm)

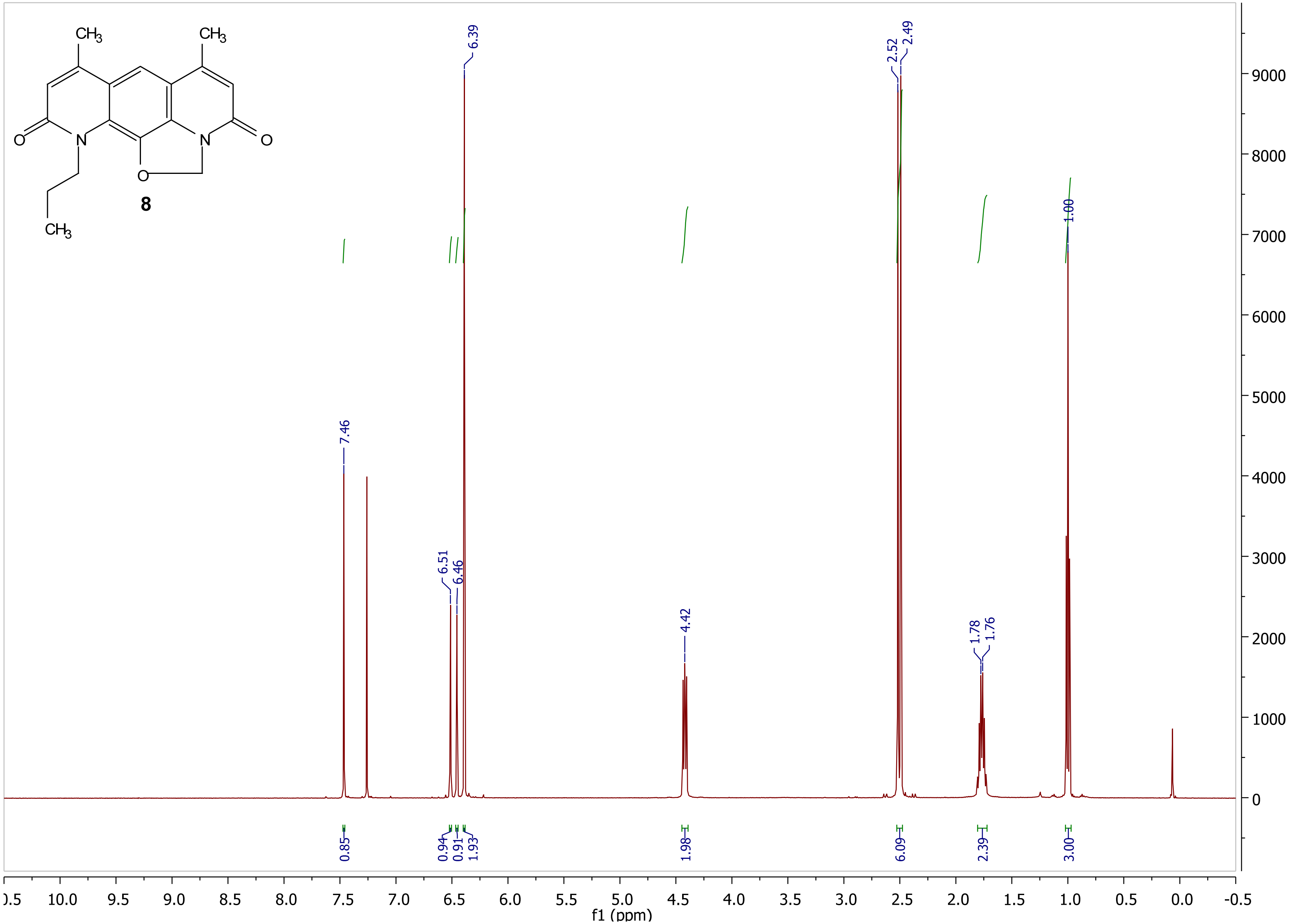

7.46
6.51
6.46
6.39
4.42
2.52
2.49
1.78
1.76
1.00
0.85
0.94
0.91
1.93
1.98
6.09
2.39
3.00
9000
8000
7000
6000
5000
4000
3000
2000
1000
0
10.0 9.5 9.0 8.5 8.0 7.5 7.0 6.5 6.0 5.5 5.0 4.5 4.0 3.5 3.0 2.5 2.0 1.5 1.0 0.5 0.0 -0.5
f1 (ppm)

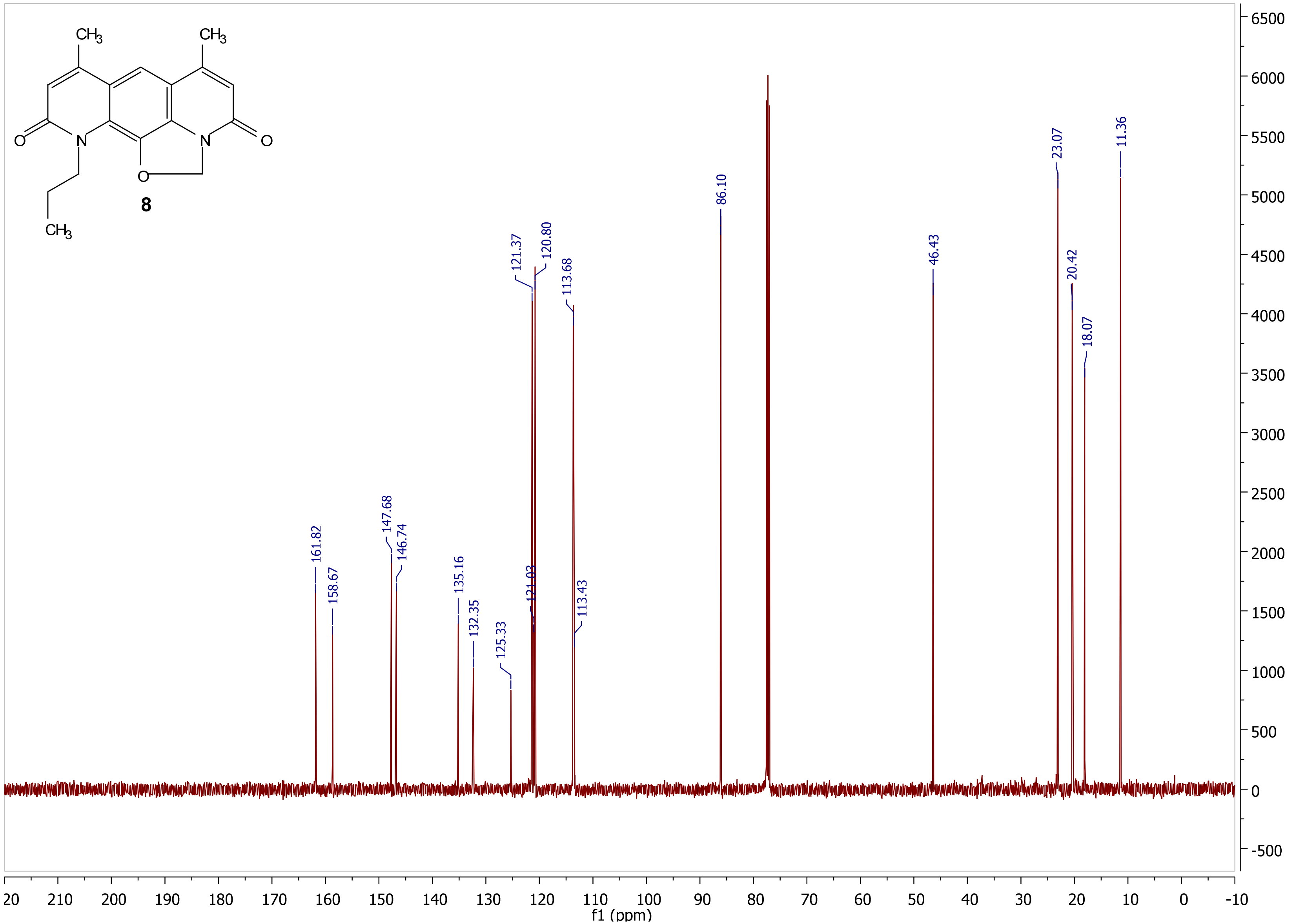
CH3
CH3
O
N
N
O
O
CH3
8
161.82
158.67
147.68
146.74
135.16
132.35
125.33
121.37
121.03
120.80
113.68
113.43
86.10
46.43
23.07
20.42
18.07
11.36
f1 (ppm)
6500
6000
5500
5000
4500
4000
3500
3000
2500
2000
1500
1000
500
0
-500
20
210
200
190
180
170
160
150
140
130
120
110
100
90
80
70
60
50
40
30
20
10
0
-10

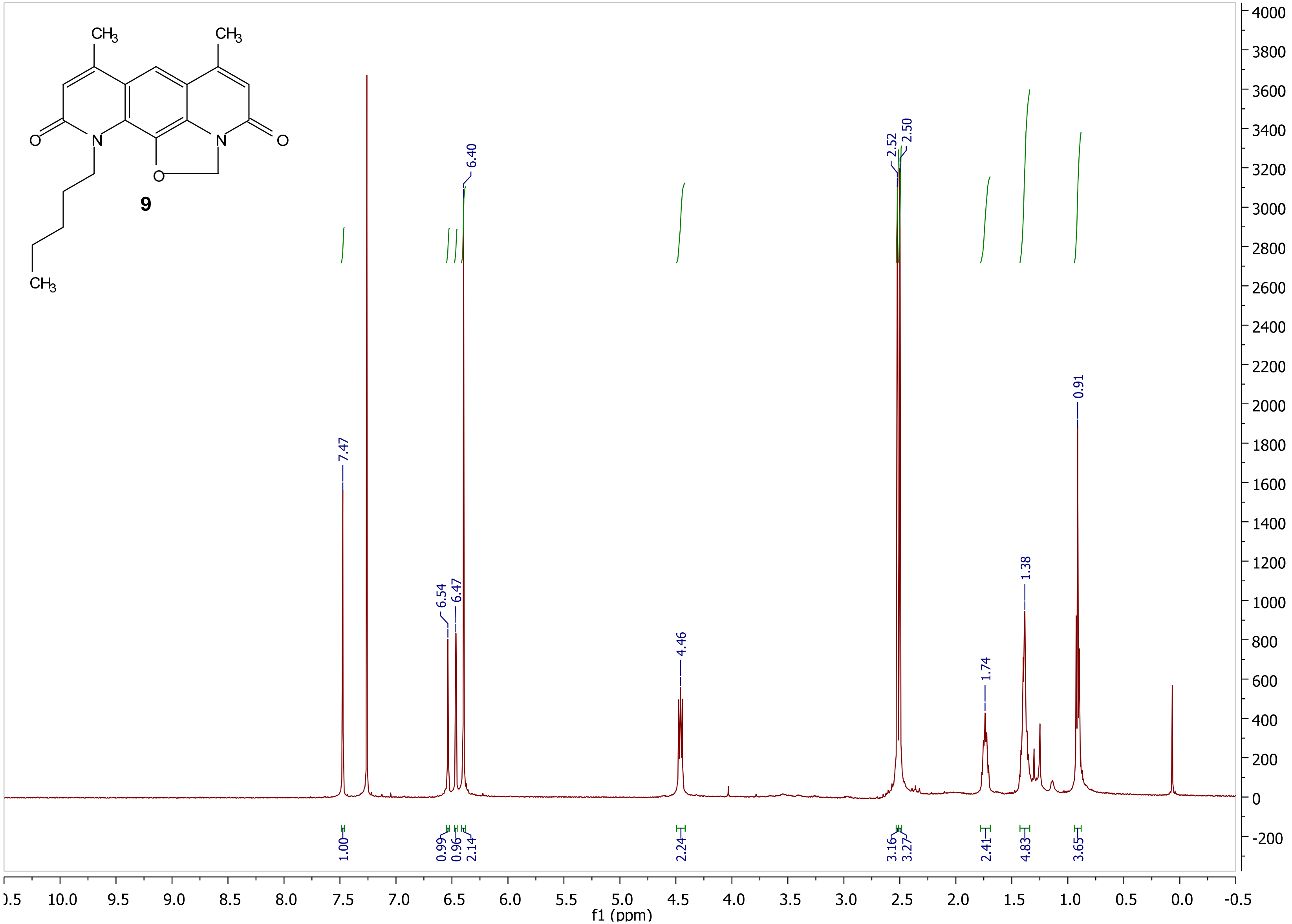

CH3
CH3
O
N
N
O
O
CH3
9
7.47
6.54
6.47
6.40
4.46
2.52
2.50
1.74
1.38
0.91
1.00
0.99
0.96
2.14
2.24
3.16
3.27
2.41
4.83
3.65
f1 (ppm)

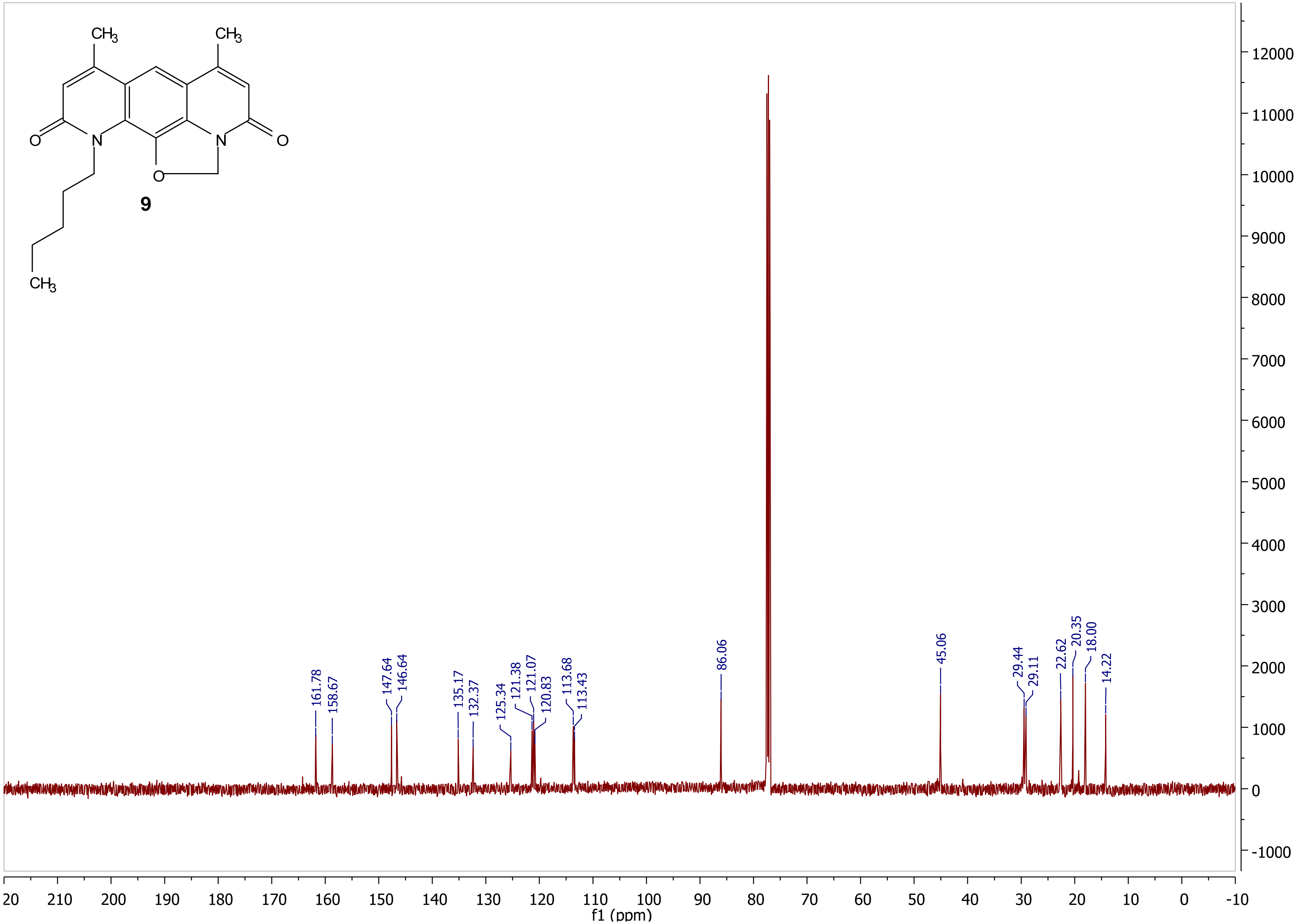

CH3
CH3
O
N
N
O
O
CH3
9
161.78
158.67
147.64
146.64
135.17
132.37
125.34
121.38
121.07
120.83
113.68
113.43
86.06
45.06
29.44
29.11
22.62
20.35
18.00
14.22
f1 (ppm)

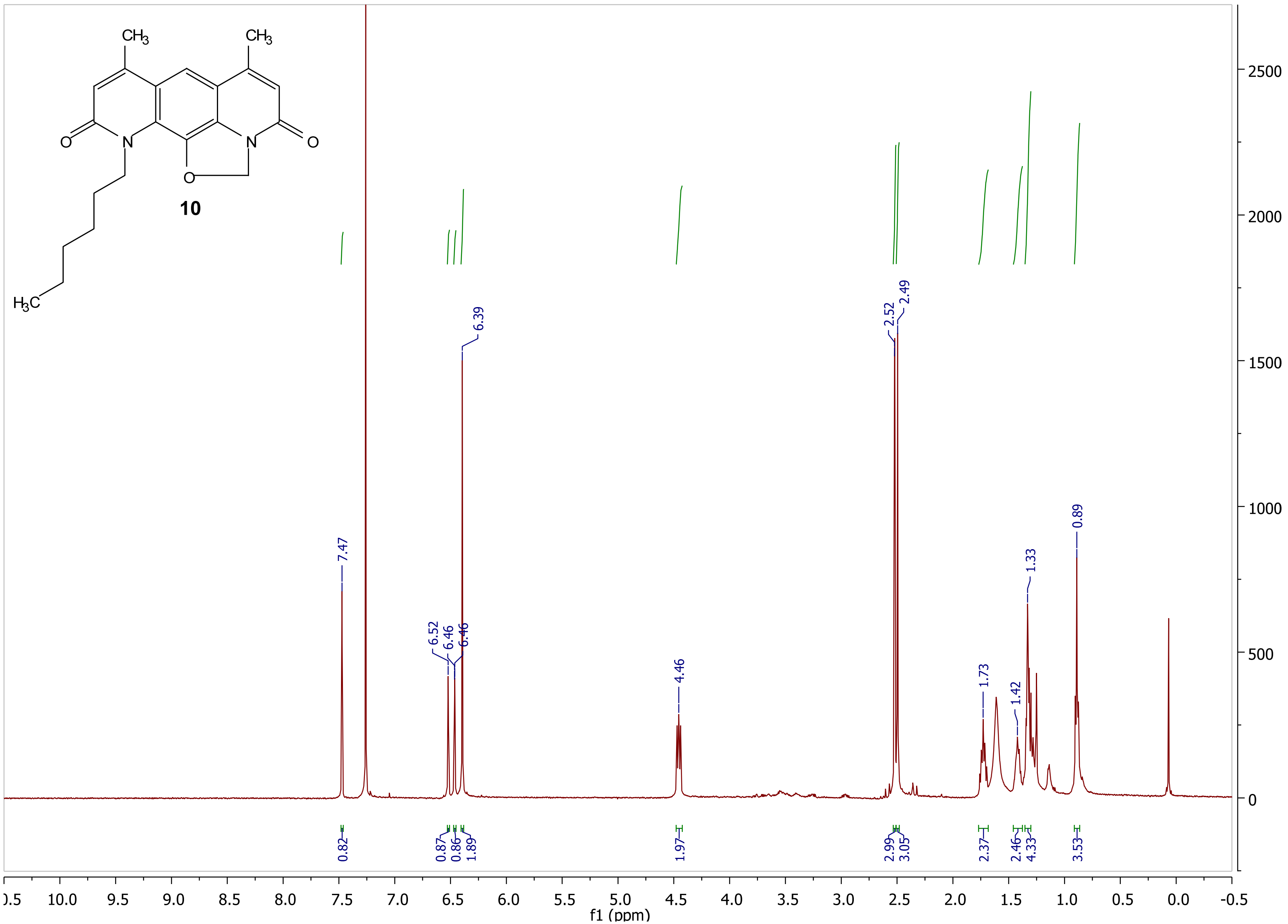
CH3
CH3
O
N
N
O
O
H3C
10
7.47
6.52
6.46
6.46
6.39
4.46
2.52
2.49
1.73
1.42
1.33
0.89
0.82
0.87
0.86
1.89
1.97
2.99
3.05
2.37
2.46
4.33
3.53
2500
2000
1500
1000
500
0
f1 (ppm)

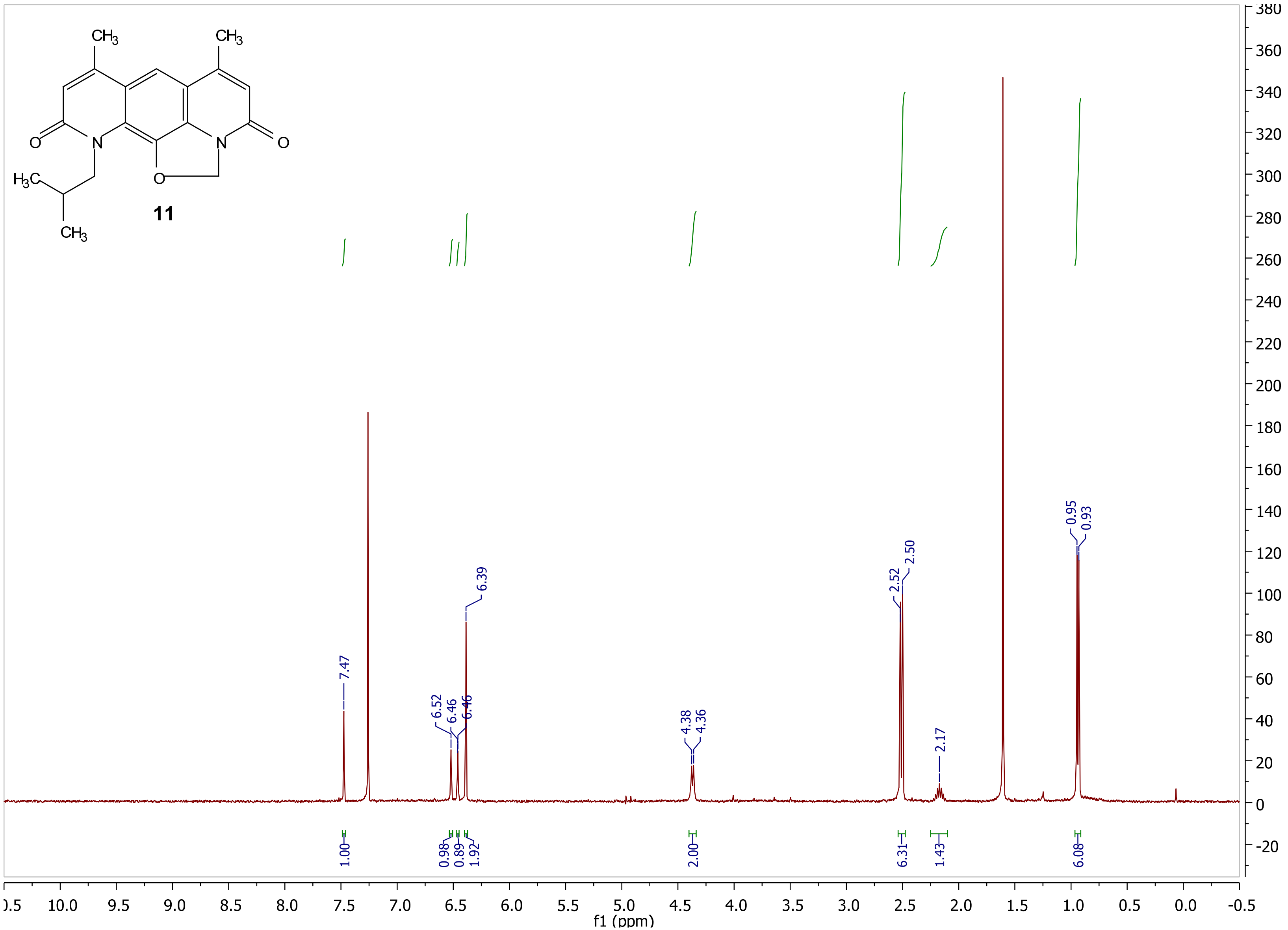
CH3
CH3
O
N
N
O
H3C
O
CH3
11
7.47
6.52
6.46
6.46
6.39
4.38
4.36
2.52
2.50
2.17
0.95
0.93
1.00
0.98
0.89
1.92
2.00
6.31
1.43
6.08
f1 (ppm)

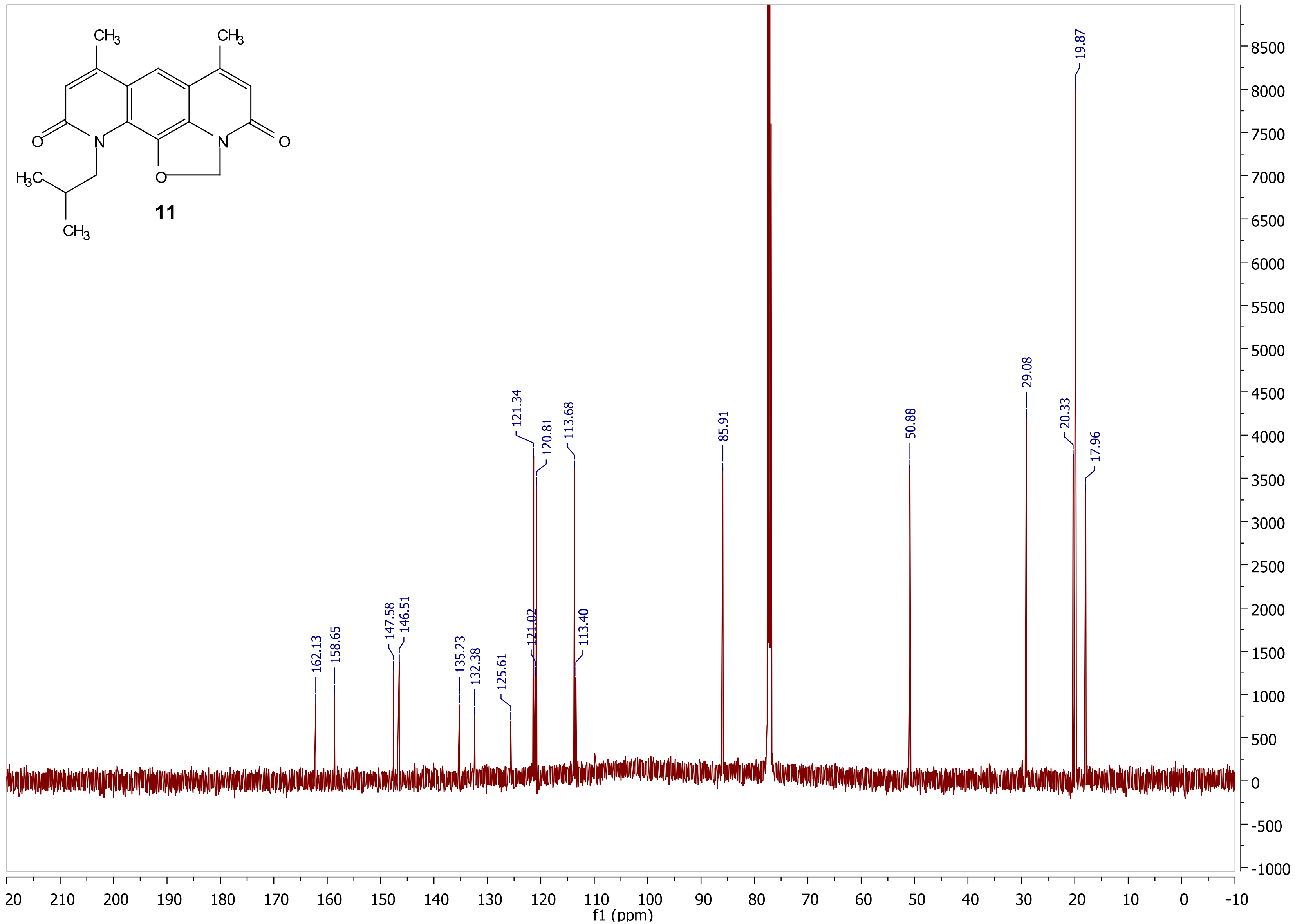
CH3
CH3
O
N
N
O
H3C
O
CH3
11
162.13
158.65
147.58
146.51
135.23
132.38
125.61
121.34
121.02
120.81
113.68
113.40
85.91
50.88
29.08
20.33
19.87
17.96
8500
8000
7500
7000
6500
6000
5500
5000
4500
4000
3500
3000
2500
2000
1500
1000
500
0
-500
-1000
20
210
200
190
180
170
160
150
140
130
120
110
100
90
80
70
60
50
40
30
20
10
0
-10
f1 (ppm)

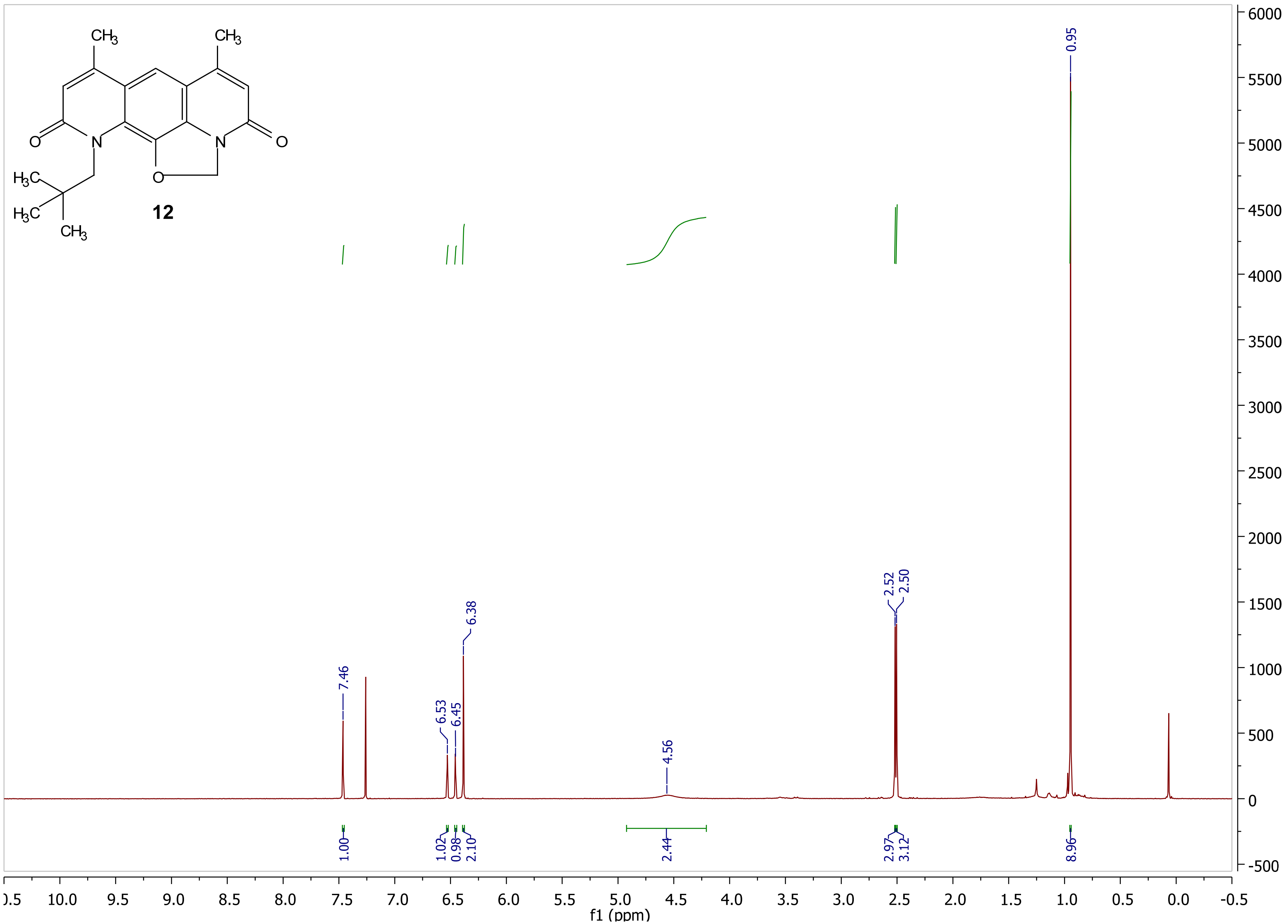
12
7.46
6.53
6.45
6.38
4.56
2.52
2.50
0.95
1.00
1.02
0.98
2.10
2.44
2.97
3.12
8.96
f1 (ppm)

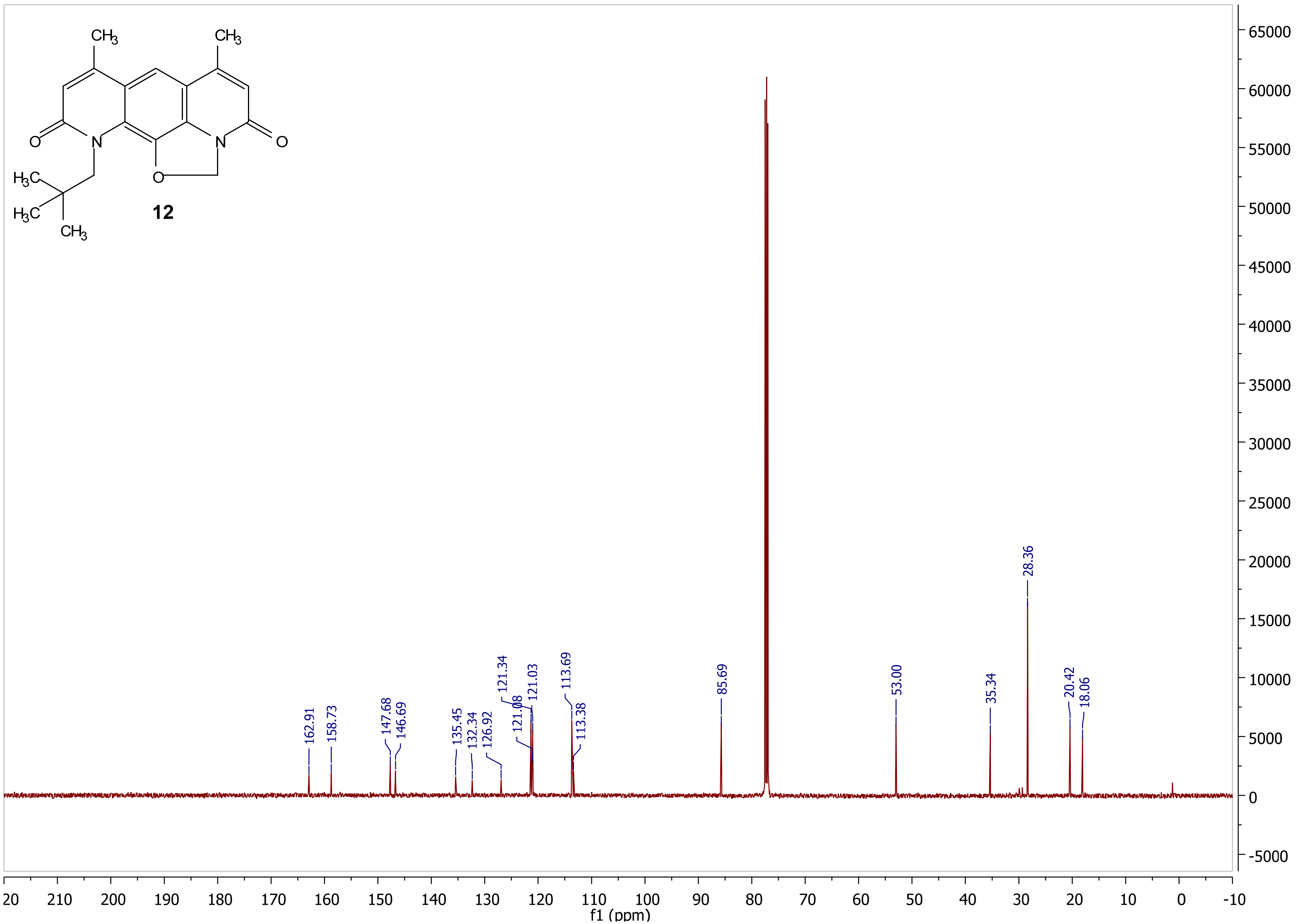
CH3
CH3
O
N
N
O
O
H3C
H3C
CH3
12
162.91
158.73
147.68
146.69
135.45
132.34
126.92
121.34
121.08
121.03
113.69
113.38
85.69
53.00
35.34
28.36
20.42
18.06
65000
60000
55000
50000
45000
40000
35000
30000
25000
20000
15000
10000
5000
0
-5000
20
210
200
190
180
170
160
150
140
130
120
110
100
90
80
70
60
50
40
30
20
10
0
-10
f1 (ppm)

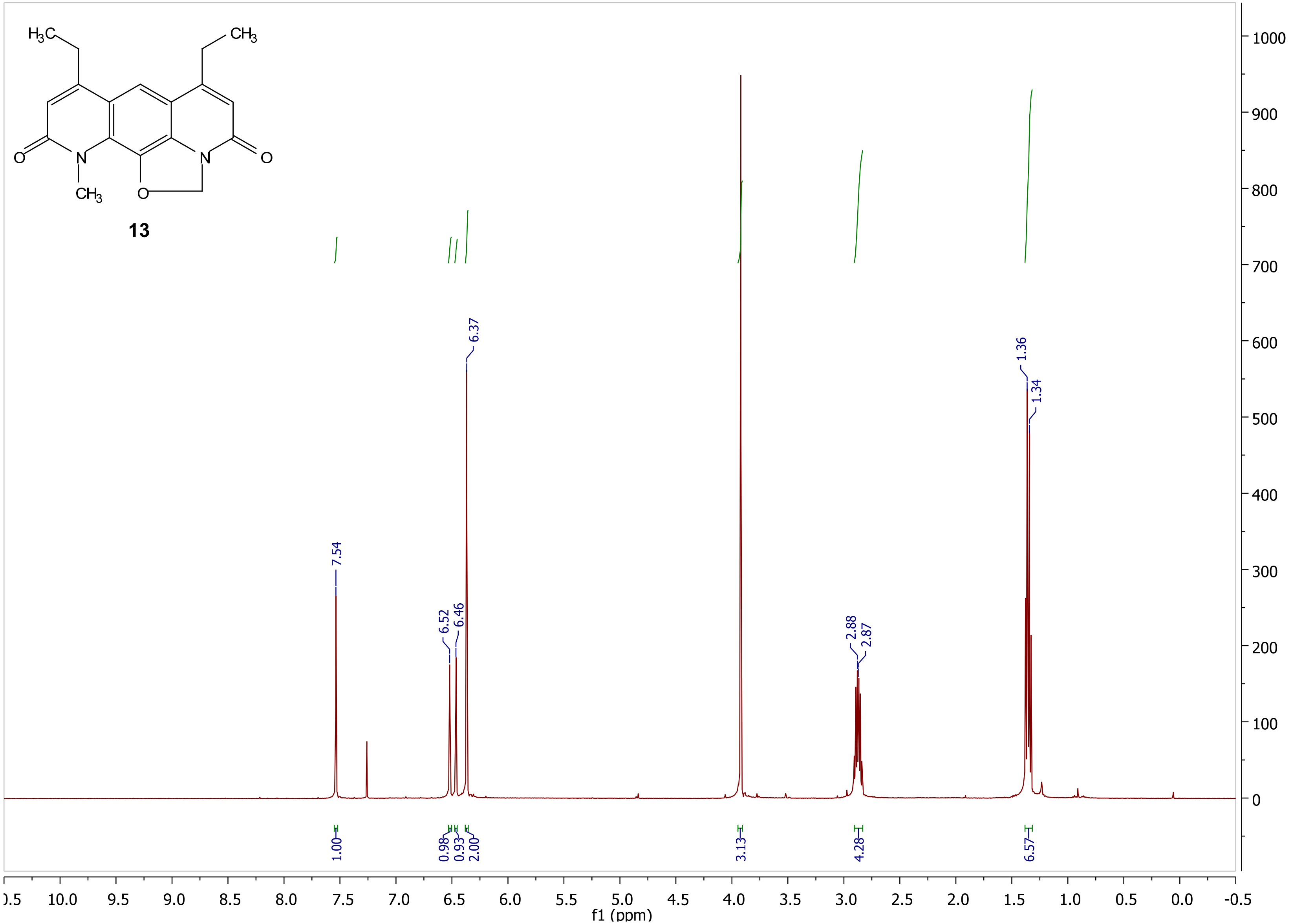
H3C
CH3
O
N
N
O
CH3
O
13
7.54
6.52
6.46
6.37
2.88
2.87
1.36
1.34
1.00
0.98
0.93
2.00
3.13
4.28
6.57
1000
900
800
700
600
500
400
300
200
100
0
10.0
9.5
9.0
8.5
8.0
7.5
7.0
6.5
6.0
5.5
5.0
4.5
4.0
3.5
3.0
2.5
2.0
1.5
1.0
0.5
0.0
-0.5
f1 (ppm)

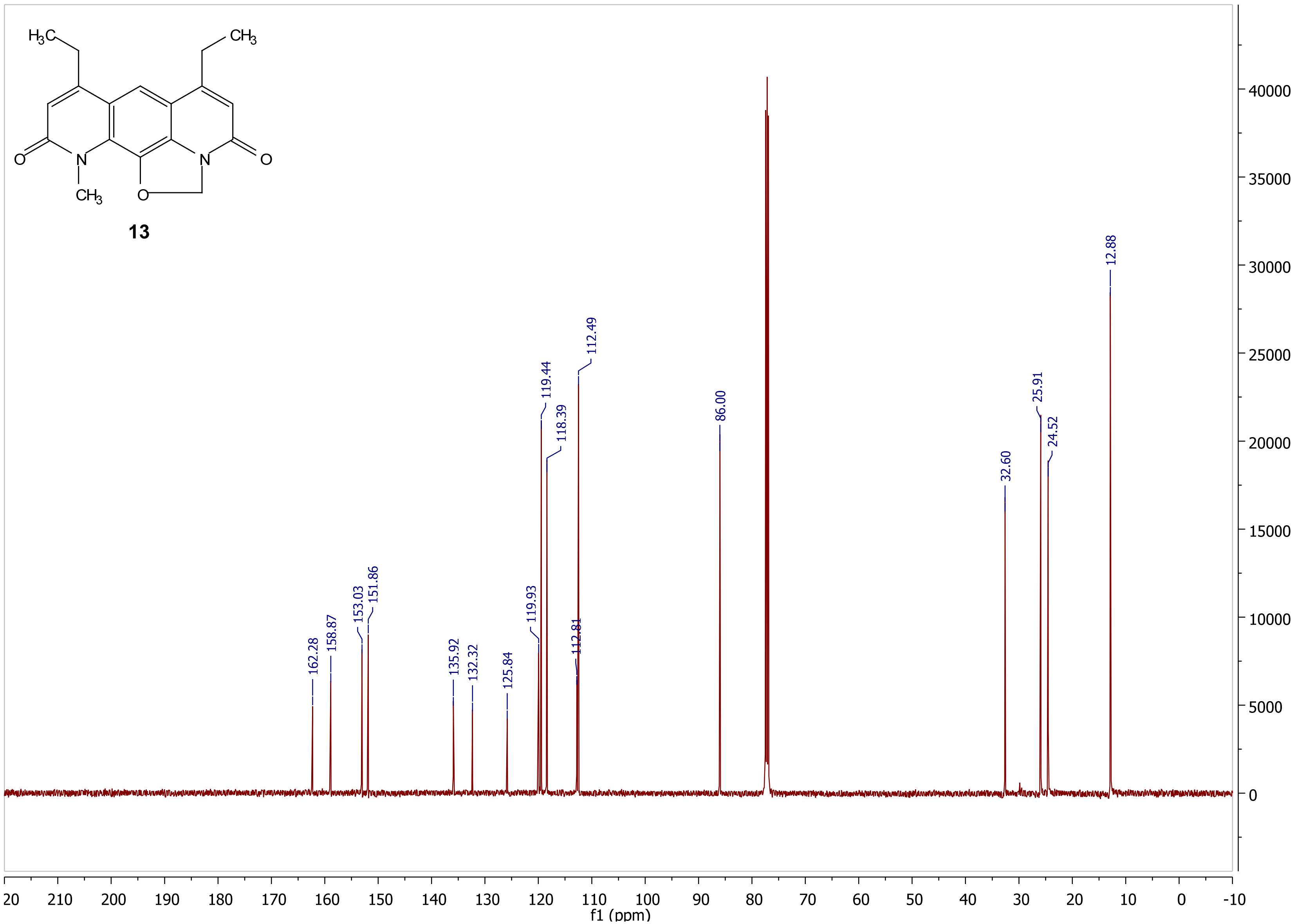
H3C
CH3
O
N
CH3
O
N
O
13
162.28
158.87
153.03
151.86
135.92
132.32
125.84
119.93
119.44
118.39
112.81
112.49
86.00
32.60
25.91
24.52
12.88
40000
35000
30000
25000
20000
15000
10000
5000
0
20
210
200
190
180
170
160
150
140
130
120
110
100
90
80
70
60
50
40
30
20
10
0
-10
f1 (ppm)

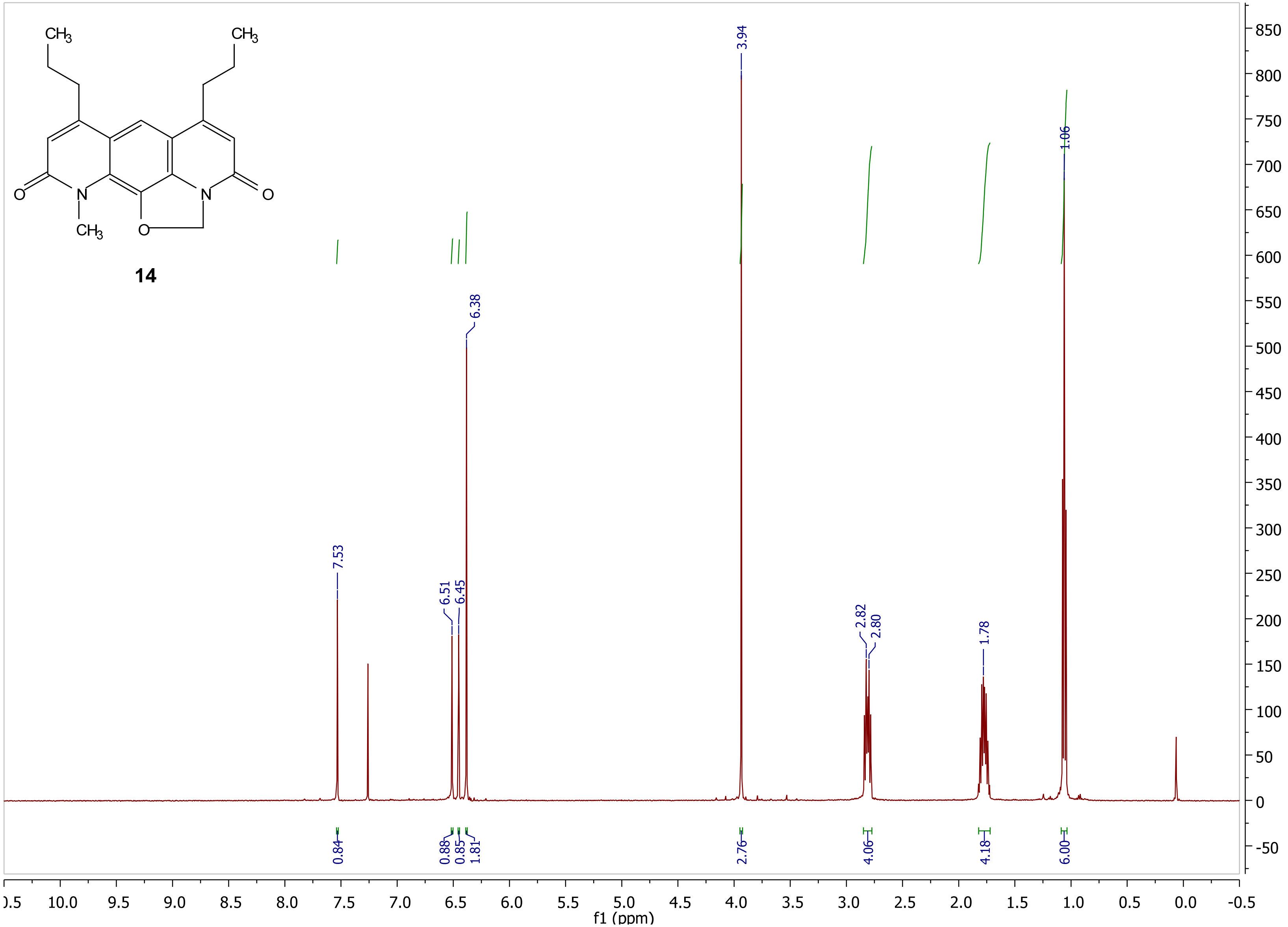
CH3
CH3
O
N
CH3
O
N
O
14
7.53
6.51
6.45
6.38
3.94
2.82
2.80
1.78
1.06
0.84
0.88
0.85
1.81
2.76
4.06
4.18
6.00
f1 (ppm)

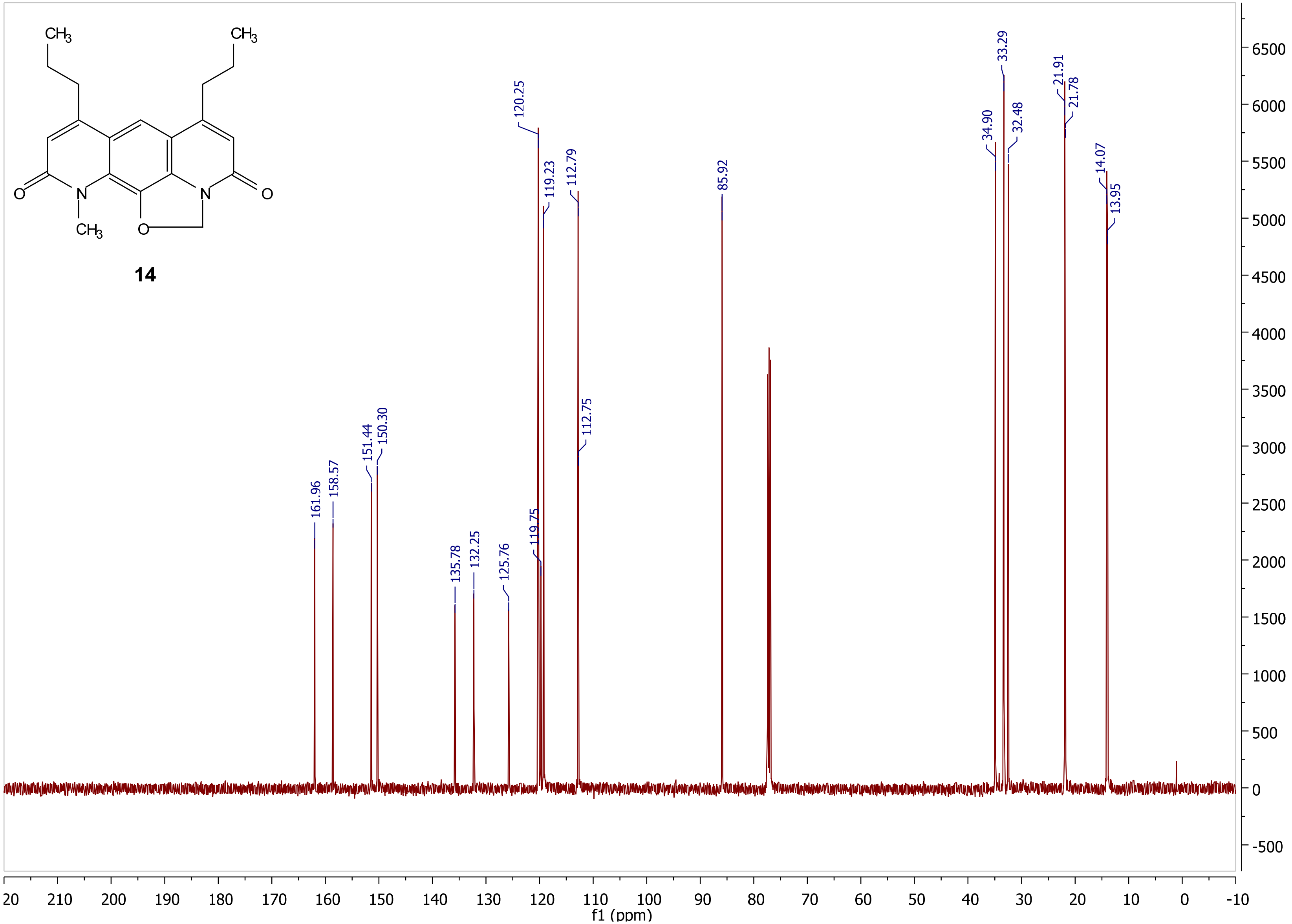
CH3
CH3
O
N
CH3
O
N
O
14
161.96
158.57
151.44
150.30
135.78
132.25
125.76
120.25
119.75
119.23
112.79
112.75
85.92
34.90
33.29
32.48
21.91
21.78
14.07
13.95
f1 (ppm)

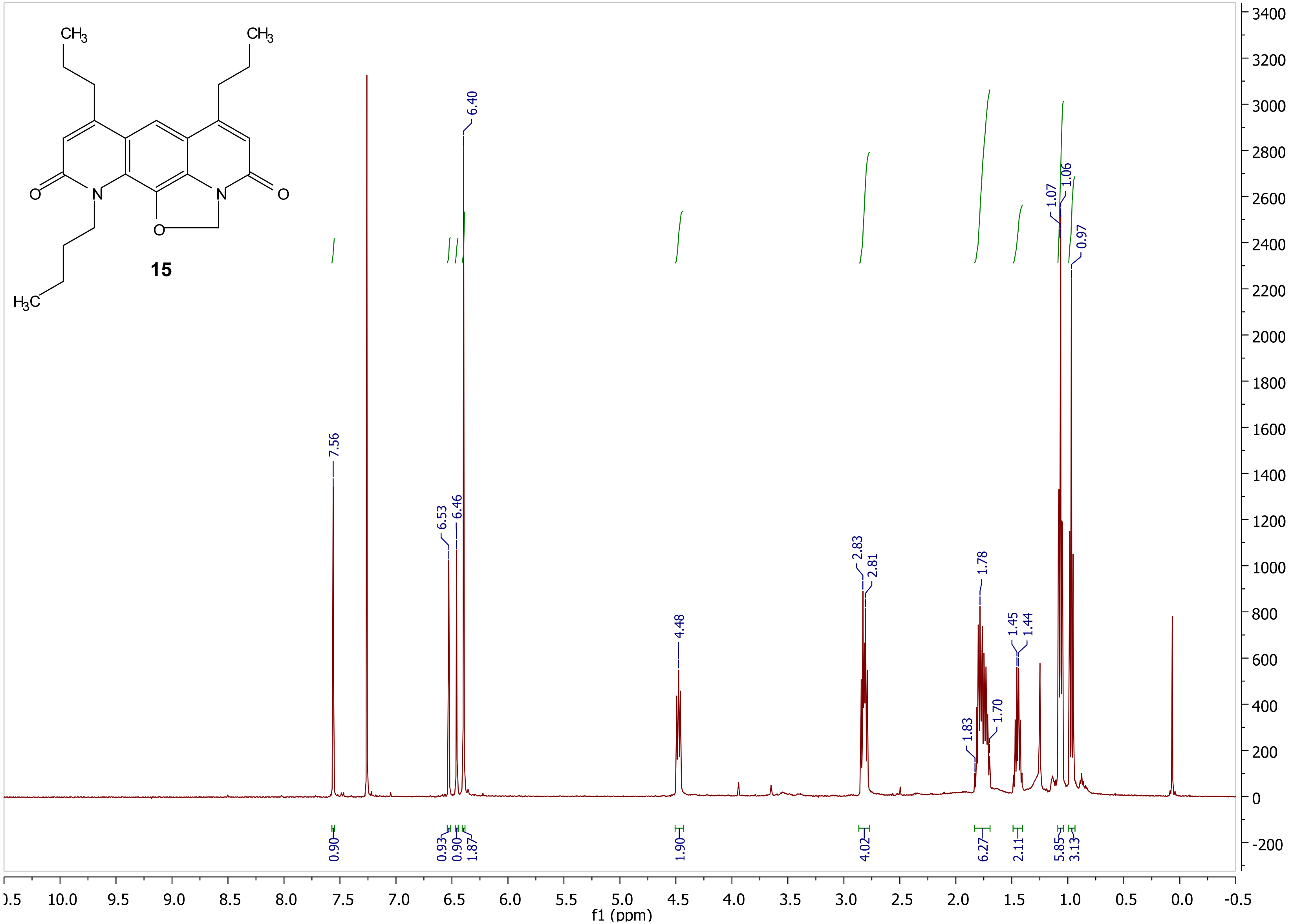
15
7.56
6.53
6.46
6.40
4.48
2.83
2.81
1.83
1.78
1.70
1.45
1.44
1.07
1.06
0.97
0.90
0.93
0.90
1.87
1.90
4.02
6.27
2.11
5.85
3.13
f1 (ppm)

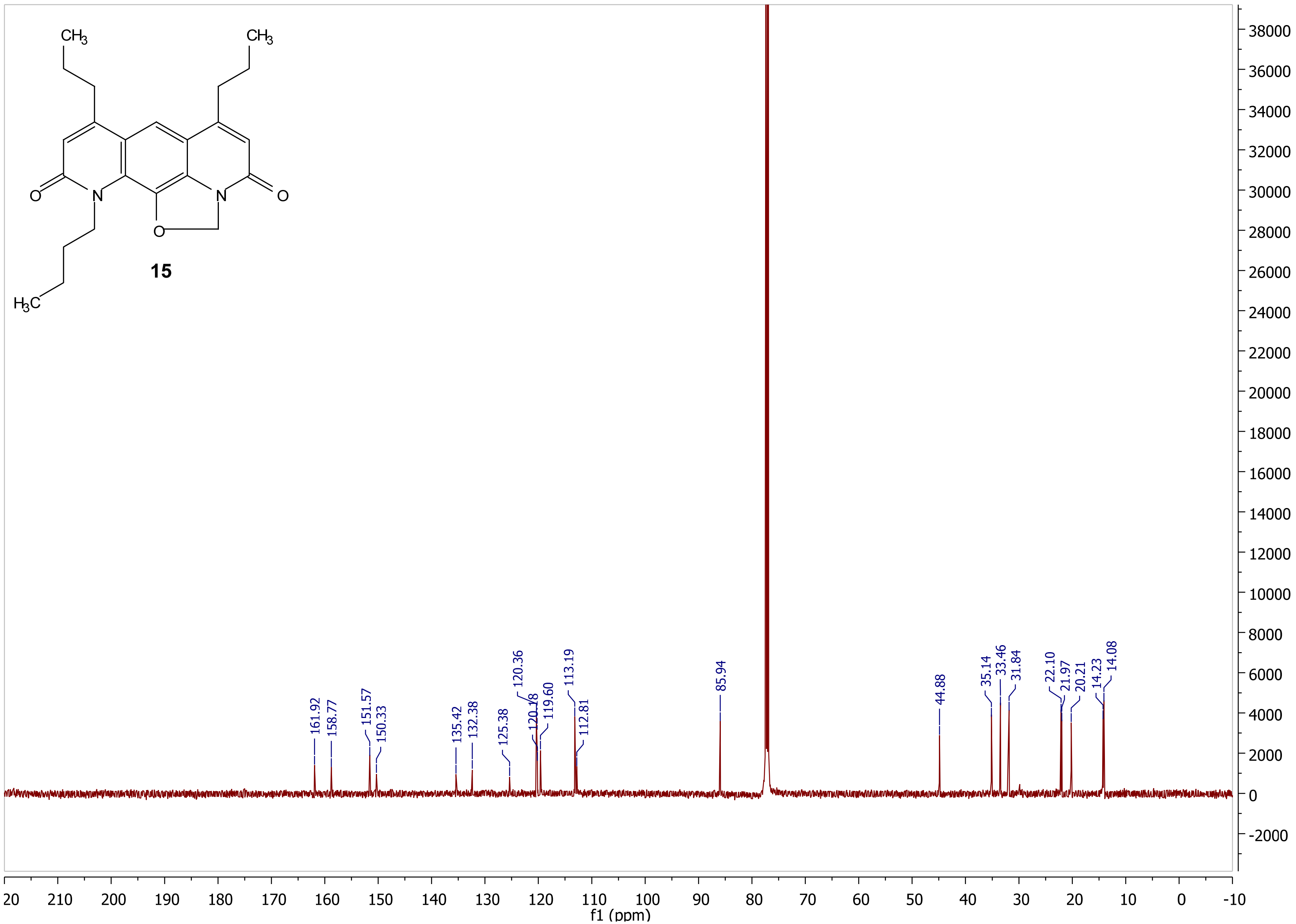
CH3
CH3
O
N
N
O
O
H3C
15
161.92
158.77
151.57
150.33
135.42
132.38
125.38
120.36
120.18
119.60
113.19
112.81
85.94
44.88
35.14
33.46
31.84
22.10
21.97
20.21
14.23
14.08
f1 (ppm)

Supplementary Methods

General chemical reagents were purchased from Sigma Aldrich. Metal catalysts and ligands were purchased from Strem Chemicals Inc. (Newburyport, MA). Alkynes were purchased from GFS Chemicals (Powell, OH) and bis-pinacolboronate was purchased from Frontier Scientific (Logan, UT). All reagents were used without further purification unless otherwise noted.

^{1}H-NMR and ^{13}C-NMR spectra were recorded on Varian Unity spectrometers at 500 MHz and 125 MHz, respectively. Spectra generated from a solution of $CDCl_3$ were referenced to residual chloroform (^{1}H: δ 7.26 ppm, ^{13}C: δ 77.16 ppm). Spectra generated in mixtures of $CDCl_3$ and CD_3OD were referenced to CD_3OD (^{1}H: δ 3.31 ppm, ^{13}C: δ 49.0 ppm).

General protocol A: Synthesis of diazaanthracenols

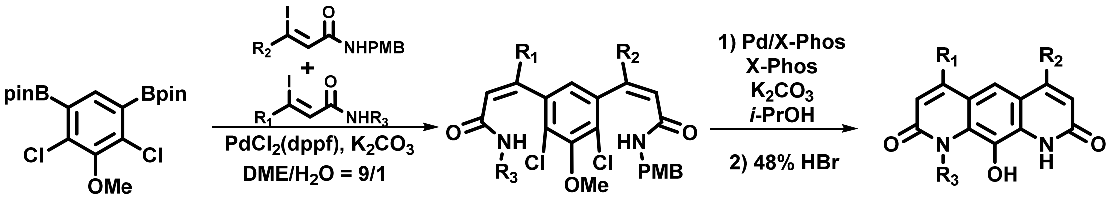

The synthesis of these diazaanthracenols has been previously described by Hergenrother and co-workers.[5-6] The only alteration from these protocols was that the phenols were further purified by reversed phase chromatography (10:90 MeCN:H_2O to 100:0 MeCN:H_2O) using a CombiFlash Rf (Teledyne Isco).

General protocol B: Synthesis of deoxynybomycins

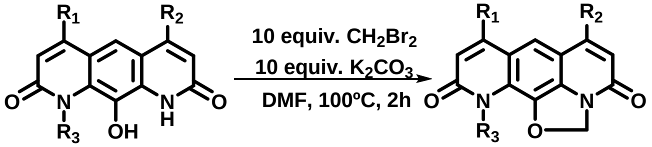

To a 20-mL vial was added diazaanthracenol (1 equiv.) and potassium carbonate (10 equiv.). The vial was evacuated and filled with argon three times. Degassed DMF (90 mL per mmol diazaanthracenol) was added followed by dibromomethane (10 equiv.). The only difference was with **5** in which 100 equiv of 1,1-dibromoethane were used in place of the dibromomethane. The vial was plunged into an oil bath preheated to 100 °C. The reaction was monitored by TLC (10% MeOH in CH_2Cl_2) with starting material appearing under UV as a green spot at the baseline and product appearing under UV as a bright blue spot at R_f = 0.5. When starting material was no longer visible by TLC (usually after 2-3 h), the solvent was evaporated and the residue was purified by silica gel chromatography (0 to 5% MeOH in CH_2Cl_2). DNM and derivatives were collected as off-white solids.

Tabulated spectra

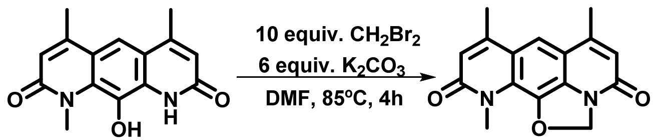

DNM

Synthesized from bispinacolborane,[5] (Z)-3-iodo-N-(4-methoxybenzyl)but-2-enamide,[5] and (Z)-3-iodo-N-methylbut-2-enamide,[5] by General Protocols A and B. 73% yield for methylene bridge insertion. 16% yield over 4 steps. 11% overall yield from commercially available starting material. Product is an off-white solid.

mp: >350 °C, 358-360 °C resulted in decomposition; ^{1}H NMR (400 MHz, 2:1 $CDCl_3$:CD_3OD): δ 7.55 (s, 1H), 6.49 (d, *J* = 1.0 Hz, 1H), 6.47 (d, *J* = 1.0 Hz, 1H), 6.39 (s, 2H), 3.92 (s, 3H), 2.54 (d, *J* = 1.0 Hz, 3H), 2.52 (d, *J* = 1.0 Hz, 3H); IR (neat, cm^{-1}): 1651 (s), 1625 (s), 1593 (s), 1558 (s), 1485 (m), 1445 (m), 1351 (s), 1327 (m), 1291 (w), 1154 (w); HRMS (m/z, ESI-TOF): $(M+H)^+$ calcd for $C_{16}H_{15}N_2O_3$, 283.1094; found, 283.1083.

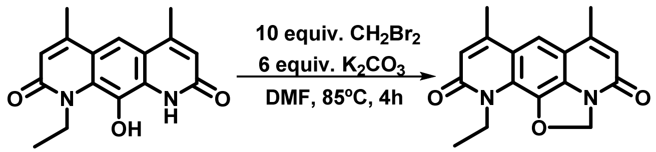

2

Synthesized from bispinacolborane,[5] (Z)-3-iodo-N-(4-methoxybenzyl)but-2-enamide,[5] and (Z)-N-ethyl-3-iodobut-2-enamide[6], by General Protocols A and B. 64% yield for methylene bridge insertion. 11% yield over 4 steps. Product is an off-white solid.

mp: >250 °C, 253 – 255 °C resulted in decomposition; ^{1}H NMR (500 MHz, $CDCl_3$): δ 7.47 (s, 1H), 6.52 (d, *J* = 1.0 Hz, 1H), 6.46 (d, *J* = 1.0 Hz, 1H), 6.40 (s, 2H), 4.54 (q, *J* = 7.0 Hz, 2H), 2.52 (d, *J* = 1.0 Hz, 3H), 2.50 (d, *J* = 1.0 Hz, 3H), 1.36 (t, *J* = 7.0 Hz, 3H); ^{13}C NMR (125 MHz, $CDCl_3$): δ 161.63, 158.64, 147.63, 146.74, 135.05, 132.32, 125.06, 121.34, 121.05, 120.79, 113.62, 113.42, 86.10, 40.27, 20.34, 18.00, 14.96; IR (neat, cm^{-1}): 1668 (m), 1657 (s), 1625 (s), 1596 (s), 1561 (m), 1485 (w), 1447 (2) 1419 (w), 1394 (m), 1380 (m), 1351 (s), 1322 (m), 1282 (w), 1243 (w), 1148 (w); HRMS (m/z, ESI-TOF): $(M+H)^+$ calcd for $C_{17}H_{17}N_2O_3$, 297.1239; found, 297.1246.

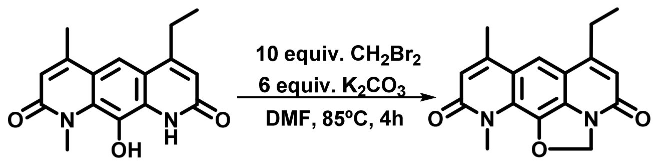

3

Synthesized from bispinacolborane,[5] (Z)-3-iodo-N-(4-methoxybenzyl)pent-2-enamide,[6] and (Z)-3-iodo-N-methylbut-2-enamide,[5] by General Protocols A and B. 72% yield for methylene bridge insertion. 13% yield over 4 steps. Product is an off-white solid.

mp: >250 °C, 274 – 275 °C resulted in decomposition; ^{1}H NMR (500 MHz, $CDCl_3$): δ 7.47 (s, 1H), 6.50 (d, *J* = 1.0 Hz, 1H), 6.47 (d, *J* = 1.0 Hz, 1H), 6.37 (s, 2H), 3.91 (s, 3H), 2.90 (q, *J* = 7.5 Hz, 2H), 2.47(s, 3H), 1.37 (t, *J* = 7.5 Hz, 3H); ^{13}C NMR (125 MHz, $CDCl_3$) δ 161.98, 158.86, 153.03, 146.76, 135.79, 132.48, 125.65, 120.71, 120.50, 119.43, 113.05, 112.86, 86.03, 32.60, 24.46, 20.27, 12.82; IR (neat, cm^{-1}): 1675 (w), 1658 (s), 1631 (s), 1598 (m), 1559 (w), 1491 (w), 1440 (w), 1414 (m), 1383 (w), 1342 (w), 1291 (2), 1147 (w); HRMS (m/z, ESI-TOF): $(M+H)^+$ calcd for $C_{17}H_{17}N_2O_3$, 297.1239; found, 297.1247

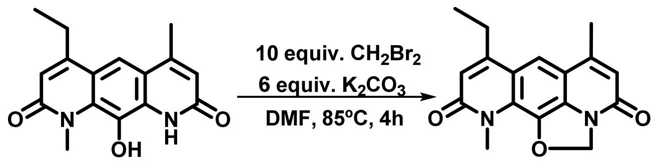

4

Synthesized from bispinacolborane,[5] (Z)-3-iodo-N-(4-methoxybenzyl)but-2-enamide,[5]and (Z)-3-iodo-N-methylpent-2-enamide,[6] by General Protocols A and B. 2% yield over 4 steps. Product is an off-white solid.

mp: >250 °C, 269 – 270 °C resulted in decomposition; ^{1}H NMR (500 MHz, $CDCl_3$): δ 7.51 (s, 1H), 6.53 (d, *J* = 1.0 Hz, 1H) 6.46 (d, *J* = 1.0 Hz, 1H), 6.38 (s, 2H), 3.94 (s, 3H), 2.88 (q, *J* = 7.5 Hz, 2H), 2.51 (d, *J* = 1.0 Hz, 3H), 1.36 (t, *J* = 7.5 Hz, 3H); ^{13}C NMR (125 MHz, $CDCl_3$) δ 162.38, 158.69, 151.94, 147.66, 135.89, 132.29, 126.09, 121.53, 120.09, 118.50, 113.56, 112.87, 86.15, 32.71, 25.95, 18.06, 12.93; IR (neat, cm^{-1}): 1651 (s), 1621 (s), 1594 (s), 1557 (w), 1490 (w), 1421 (m), 1354 (m), 1328 (m), 1292 (w), 1154 (w); HRMS (m/z, ESI-TOF): $(M+H)^+$ calcd for $C_{17}H_{17}N_2O_3$, 297.1239; found: 297.1234

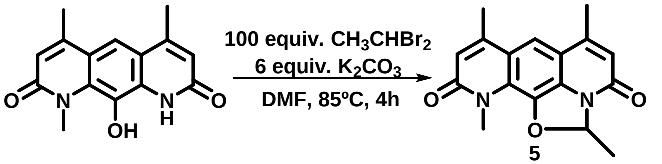

5

Synthesized from bispinacolborane,[5] (Z)-3-iodo-N-(4-methoxybenzyl)but-2-enamide,[5] and (Z)-3-iodo-N-methylbut-2-enamide,[5] by General Protocols A and B. General Protocol B was altered slightly. Specifically, 1,2-dibromoethane was used in place of dibromomethane and 100 equivalents were used instead of 10. 4% yield over 4 steps. Product is a yellow/off-white solid.

^{1}H NMR (500 MHz, $CDCl_3$): δ 7.43 (s, 1H), 6.80 (q, J = 5.5 Hz, 1H), 6.51 (d, J = 1.0 Hz, 1H), 6.44 (d, J = 1.0 Hz, 1H), 3.93 (s, 3H), 2.50 (d, J = 1.0 Hz, 3H), 2.49 (d, J = 1.0 Hz, 3H), 1.96 (d, J = 5.5 Hz, 3H); ^{13}C NMR (125 MHz, $CDCl_3$) δ 163.09, 159.71, 148.92, 148.49, 135.09, 132.19, 125.52, 121.65, 121.45, 119.96, 114.40, 113.74, 96.44, 32.99, 20.34, 20.22, 17.92; HRMS (m/z, ESI-TOF): $(M+H)^+$ calcd for $C_{17}H_{17}N_2O_3$, 297.1239; found, 297.1244.

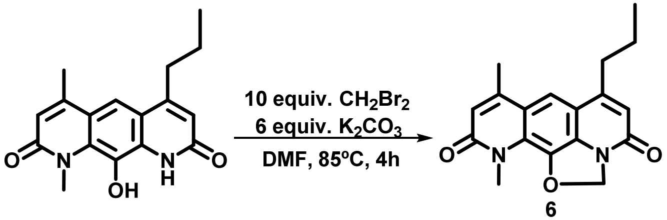

6

Synthesized from bispinacolborane,[5] (Z)-3-iodo-N-(4-methoxybenzyl)hex-2-enamide,[6] and (Z)-3-iodo-N-methylbut-2-enamide,[5] by General Protocols A and B. 2% yield over 4 steps. Product is an off-white solid.

mp: >200 °C, 228 – 230 °C resulted in decomposition; ^{1}H NMR (500 MHz, $CDCl_3$) δ 7.48 (s, 1H), 6.52 (1H), 6.46 (1H), 6.39 (s, 2H), 3.93 (s, 3H), 2.83 (t, J = 7.5 Hz, 2H), 2.49 (3H), 1.80 (m, 2H), 1.06 (t, J = 7.5 Hz, 3H); ^{13}C NMR (125 MHz, $CDCl_3$): δ 162.01, 158.76, 151.62, 146.73, 135.84, 132.60, 125.70, 120.74, 120.55, 120.40, 113.24, 113.01, 86.06, 33.39, 32.61, 21.96, 20.24, 14.05; IR (neat, cm^{-1}): 1660 (s), 1636 (s), 1600 (s), 1558 (w), 1489 (w), 1442 (w), 1416 (w), 1382 (w), 1344 (m), 1277 (w), 1148 (w); HRMS (m/z, ESI-TOF): $(M+H)^+$ calcd for $C_{18}H_{19}N_2O_3$, 311.1396; found: 311.1405

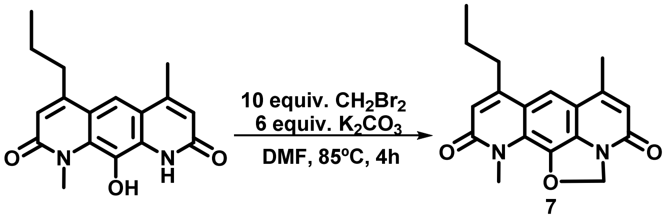

7

Synthesized from bispinacolborane,[5] (Z)-3-iodo-N-(4-methoxybenzyl)but-2-enamide,[5]and (Z)-3-iodo-N-methylhex-2-enamide,[6] by General Protocols A and B. 1% yield over 4 steps. Product is an off-white solid.

^{1}H NMR (500 MHz, $CDCl_3$): δ 7.51 (s, 1H), 6.52 (d, *J* = 1.0 Hz, 1H), 6.46 (d, *J* = 1.0 Hz, 1H), 6.39 (s, 2H), 3.95 (s, 3H), 2.81 (t, 2H, *J* = 7.5 Hz), 2.52 (d, 3H, *J* = 1.0 Hz, allylic C*H3*), 1.78 (m, 2H), 1.07 (t, 3H, *J* = 7.5 Hz); ^{13}C NMR (125 MHz, $CDCl_3$): δ 162.16, 158.61, 150.46, 147.57, 135.82, 132.19, 126.09, 121.43, 120.03, 119.38, 113.46, 113.01, 86.07, 34.98, 32.64, 21.83, 17.97, 14.19; HRMS (m/z, ESI-TOF): $(M+H)^+$ calcd for $C_{18}H_{19}N_2O_3$, 311.1396; found, 311.1393.

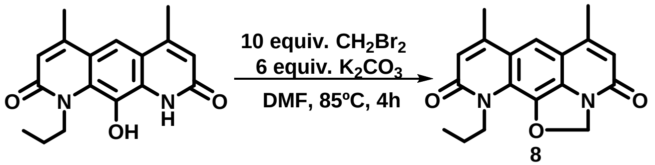

8

Synthesized from bispinacolborane,[5] (Z)-3-iodo-N-(4-methoxybenzyl)but-2-enamide,[5] and (Z)-3-iodo-N-propylbut-2-enamide,[6] by General Protocols A and B. 2% yield over 4 steps. Product is an off-white solid.

mp: >250 °C, 248 – 250 °C resulted in decomposition; ^{1}H NMR (500 MHz, $CDCl_3$): δ 7.46 (s, 1H), 6.51(1H), 6.46 (1H), 6.39 (s, 2H), 4.42 (m, 2H), 2.52 (3H), 2.49 (3H), 1.78 (m, 2H), 1.00 (t, *J* = 7.5 Hz, 3H); ^{13}C NMR (125 MHz, $CDCl_3$): δ 161.82, 158.67, 147.68, 146.74, 135.16, 132.35, 125.33, 121.37, 121.03, 120.80, 113.68, 113.43, 86.10, 46.43, 23.07, 20.42, 18.07, 11.36; IR (neat, cm^{-1}): 1655 (s), 1623 (s), 1594 (m), 1556 (w), 1485 (w), 1439 (w), 1399 (w), 1352 (m), 1229 (w), 1154 (w); HRMS (m/z, ESI-TOF): $(M+H)^+$ calcd for $C_{18}H_{19}N_2O_3$, 311.1396; found, 311.1398

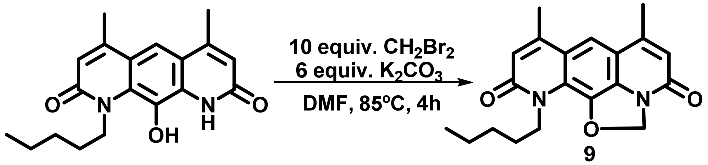

9

Synthesized from bispinacolborane,[5] (Z)-3-iodo-N-(4-methoxybenzyl)but-2-enamide,[5]and (Z)-3-iodo-N-pentylbut-2-enamide,[6] by General Protocols A and B. 6% yield over 4 steps. Product is an off-white solid.

^{1}H NMR (500 MHz, $CDCl_3$): δ 7.47 (s, 1H6.54 (d, *J* = 1.0 Hz, , 1H), 6.47 (d, *J* = 1.0 Hz, 1H), 6.40 (s, 2H), 4.46 (m, 2H), 2.52 (d, *J* = 1.0 Hz, 3H), 2.50 (d, *J* = 1.0 Hz, 3H), 1.74 (pent, *J* = 7.5 Hz, 2H) , 1.38 (m, 4H), 0.91 (t, *J* = 7.0 Hz, 3H); ^{13}C NMR (125 MHz, $CDCl_3$): δ 161.78, 158.67, 147.64, 146.64, 135.17, 132.37, 125.34, 121.38, 121.07, 120.83, 113.68, 113.43, 86.06, 45.06, 29.44, 29.11, 22.62, 20.35, 18.00, 14.22; HRMS (m/z, ESI-TOF): $(M+H)^+$ calcd for $C_{20}H_{23}N_2O_3$, 339.1709; found, 339.1704.

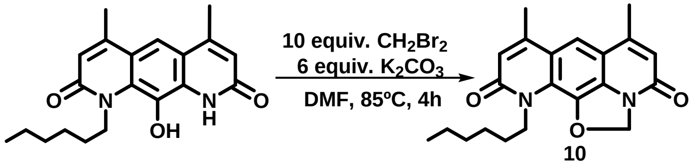

Synthesized from bispinacolborane,[5] (Z)-3-iodo-N-(4-methoxybenzyl)but-2-enamide,[5] and (Z)-N-hexyl-3-iodobut-2-enamide,[6] by General Protocols A and B. 20% yield over 4 steps. Product is an off-white solid.

^{1}H NMR (500 MHz, $CDCl_3$): δ 7.47 (s, 1H), 6.52 (d, *J* = 1.0 Hz, 1H), 6.46 (d, *J* = 1.0 Hz, 1H), 6.39 (s, 2H), 4.46 (m, 2H), 2.52 (d, *J* = 1.0 Hz, 3H), 2.49 (d, *J* = 1.0 Hz, 3H), 1.73 (pent, *J* = 7.5 Hz, 2H), 1.42 (pent, *J* = 7.5 Hz, 2H), 1.33 (m, 4H), 0.89 (t, *J* = 7.0 Hz, 3H); HRMS (m/z, ESI-TOF): $(M+H)^+$ calcd for $C_{21}H_{25}N_2O_3$, 353.1865; found: 353.1870

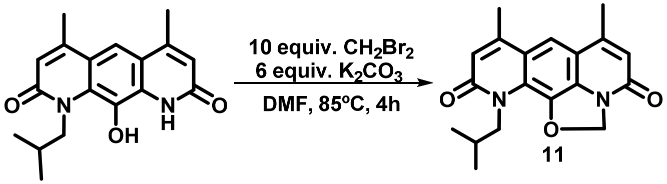

Synthesized from bispinacolborane,[5] (Z)-3-iodo-N-(4-methoxybenzyl)but-2-enamide,[5]and (Z)-3-iodo-N-isobutylbut-2-enamide,[6] by General Protocols A and B. 62% yield for methylene bridge insertion. 6% yield over 4 steps. Product is an off-white solid.

^{1}H NMR (400 MHz, $CDCl_3$): δ 7.47 (s, 1H), 6.52 (d, *J* = 1.0 Hz, 1H 6.46 (d, *J* = 1.0 Hz, 1H), 6.39 (s, 2H), 4.38 (d, *J* = 7.2 Hz, 2H), 2.52 (d, *J* = 1.0 Hz, 3H), 2.50 (d, *J* = 1.0 Hz, 3H), 2.17 (sept, *J* = 7.2 Hz, 1H), 0.95 (d, *J* = 6.8 Hz, 1H); ^{13}C NMR (125 MHz, $CDCl_3$): δ 162.13, 158.65, 147.58, 146.51, 135.23, 132.38, 125.61, 121.34, 121.02, 120.81, 113.68, 113.40, 85.91, 50.88, 29.08, 20.33, 19.87, 17.96; HRMS (m/z, ESI-TOF): $(M+H)^+$ calcd for $C_{19}H_{21}N_2O_3$, 325.1552; found, 325.1553.

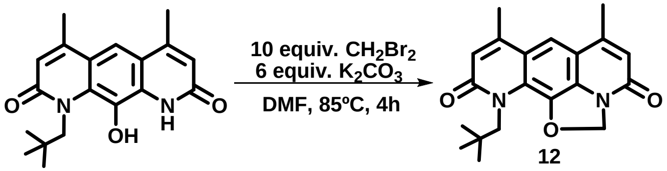

Synthesized from bispinacolborane,[5] (Z)-3-iodo-N-(4-methoxybenzyl)but-2-enamide,[5] and (Z)-3-iodo-N-neopentylbut-2-enamide,[6] by General Protocols A and B. 30% yield over 4 steps. Product is an off-white solid.

mp: >200 °C, 210 – 213 °C resulted in decomposition; ^{1}H NMR (500 MHz, $CDCl_3$): δ 7.46 (s, 1H), 6.53 (d, *J* = 1.0 Hz, 1H), 6.45 (d, *J* = 1.0 Hz, 1H), 6.38 (s, 2H), 4.56 (bs, 1H), 2.52 (d, *J* = 1.0 Hz, 3H), 2.50 (d, *J* = 1.0 Hz, 3H), 0.95 (s, 9H); ^{13}C NMR (125 MHz, $CDCl_3$): δ 162.91, 158.73, 147.68, 146.69, 135.45, 132.34, 126.92, 121.34, 121.08, 121.03, 113.69, 113.38, 85.69, 53.00, 35.34, 28.36, 20.42, 18.06; IR (neat, cm^{-1}): 1698 (w), 1658 (s),

1631 (s), 1606 (s), 1560 (w), 1474 (w), 1447 (w), 1353 (m), 1313 (w), 1258 (w), 1137 (m); HRMS (m/z, ESI-TOF): $(M+H)^+$ calcd for $C_{20}H_{23}N_2O_3$, 339.1709; found, 339.1715.

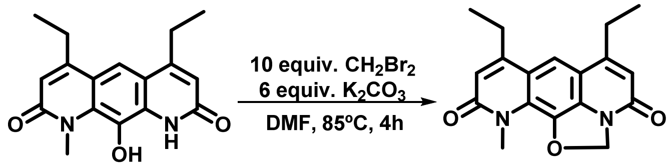

13

Synthesized from bispinacolborane,[5] (Z)-3-iodo-N-(4-methoxybenzyl)pent-2-enamide,[6] and ((Z)-3-iodo-N-methylpent-2-enamide,[6] by General Protocols A and B. 7% yield over 4 steps. Product is an off-white solid.

mp: >200 °C, 214 – 215 °C resulted in decomposition; ^{1}H NMR (500 MHz, $CDCl_3$): δ 7.54 (s, 1H), 6.52 (1H), 6.46 (1H), 6.37 (s, 2H), 3.92 (s, 3H), 2.88 (dq, J = 1.0 Hz, 7.5 Hz, 2H), 2.87 (dq, J = 1.0 Hz, 7.5 Hz, 2H), 1.36 (t, J = 7.5 Hz, 3H), 1.34 (t, J = 7.5 Hz, 3H); ^{13}C NMR (125 MHz, $CDCl_3$): δ 162.28, 158.87, 153.03, 151.86, 135.92, 132.32, 125.84, 119.93, 119.44, 118.39, 112.81, 112.49, 86.00, 32.60, 25.91, 24.52, 12.88; IR (neat, cm^{-1}): 1682 (w), 1657 (s), 1631 (s), 1595 (s), 1558 (w), 1455 (w), 1416 (m), 1370 (w), 1339 (m), 1261 (w), 1145 (w); HRMS (m/z, ESI-TOF): $(M+H)^+$ calcd for $C_{18}H_{19}N_2O_3$, 311.1396; found, 311.1393

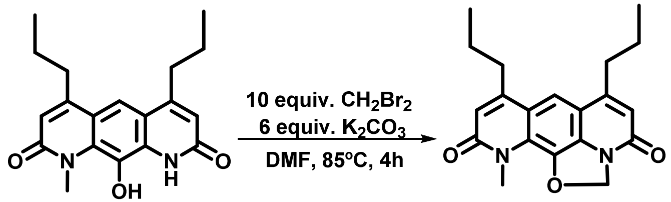

14

Synthesized from bispinacolborane,[5] (Z)-3-iodo-N-(4-methoxybenzyl)hex-2-enamide,[6] and (Z)-3-iodo-N-methylhex-2-enamide,[6] by General Protocols A and B. 5% yield over 4 steps. Product is an off-white solid.

mp: 171-173 °C; ^{1}H NMR (500 MHz, $CDCl_3$): δ 7.53 (s, 1H), 6.51 (1H), 6.45 (1H), 6.38 (s, 2H), 3.94 (s, 3H), 2.82 (t, J = 7.5 Hz, 2H), 2.80 (t, J = 7.5 Hz, 2H), 1.78 (m, 4H), 1.06 (t, J = 7.5 Hz, 6H); ^{13}C NMR (125 MHz, $CDCl_3$): δ 161.96, 158.57, 151.44, 150.30, 135.78, 132.25, 125.76, 120.25, 119.75, 119.23, 112.79, 112.75, 85.92, 34.90, 33.29, 32.48, 21.91, 21.78, 14.07, 13.95; IR (neat, cm^{-1}): 1652 (s), 1627 (s), 1596 (s), 1556 (w), 1487 (w), 1458 (w), 1427 (m), 1378 (w), 1340 (m), 1326 (m), 1282 (w), 1143 (w); HRMS (m/z, ESI-TOF): $(M+H)^+$ calcd for $C_{20}H_{23}N_2O_3$, 339.1709; found, 339.1717

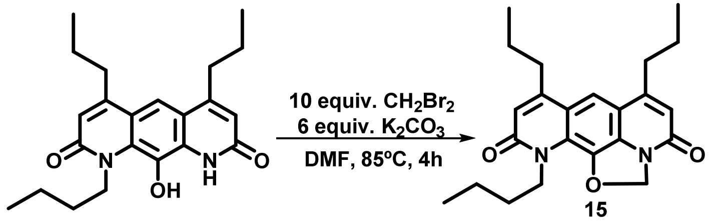

Synthesized from bispinacolborane,[5] (Z)-3-iodo-N-(4-methoxybenzyl)hex-2-enamide,[6] and (Z)-N-butyl-3-iodohex-2-enamide[6] by General Protocols A and B. 6% yield over 4 steps. Product is an off-white solid.

mp: 156-158 °C; ^{1}H NMR (500 MHz, $CDCl_3$) δ 7.56 (s, 1H), 6.53(1H), 6.46(1H), 6.40(s, 2H), 4.48 (m, 2H), 2.83 (t, *J* = 7.5 Hz, 2H), 2.81 (t, *J* = 7.5 Hz, 2H), 1.70-1.83 (m, 6H), 1.45 (sext, *J* = 7.5 Hz, 2H), 1.07 (t, *J* = 7.5 Hz, 3H), 1.06 (t, *J* = 7.5 Hz, 3H), 0.97 (t, *J* = 7.5 Hz, 3H); ^{13}C NMR (125 MHz, $CDCl_3$): δ 161.92, 158.77, 151.57, 150.33, 135.42, 132.38, 125.38, 120.36, 120.18, 119.60, 113.19, 112.81, 85.94, 44.88, 35.14, 33.46, 31.84, 22.10, 21.97, 20.21, 14.23, 14.08; IR (neat, cm^{-1}): 1658 (s), 1629 (s), 1596 (m), 1557 (w), 1485 (w), 1445 (w), 1422 (w), 1397 (w), 1336 (w), 1315 (w), 1271 (w), 1219 (w); HRMS (m/z, ESI-TOF): $(M+H)^+$ calcd for $C_{23}H_{29}N_2O_3$, 381.2178; found, 381.2167

References

1 Williams, J. J., Halvorsen, E. M., Dwyer, E. M., DiFazio, R. M. & Hergenrother, P. J. Toxin-antitoxin (TA) systems are prevalent and transcribed in clinical isolates of Pseudomonas aeruginosa and methicillin-resistant Staphylococcus aureus. *FEMS Microbiol Lett* **322**, 41-50, (2011).

2 Bratu, S., Landman, D., Martin, D. A., Georgescu, C. & Quale, J. Correlation of antimicrobial resistance with beta-lactamases, the OmpA-like porin, and efflux pumps in clinical isolates of Acinetobacter baumannii endemic to New York City. *Antimicrob Agents Chemother* **52**, 2999-3005, (2008).

3 Schnell, M. A., Hardy, C., Hawley, M., Propert, K. J. & Wilson, J. M. Effect of blood collection technique in mice on clinical pathology parameters. *Hum Gene Ther* **13**, 155-161, (2002).

4 Schneider, C. A., Rasband, W. S. & Eliceiri, K. W. NIH Image to ImageJ: 25 years of image analysis. *Nat Methods* **9**, 671-675, (2012).

5 Bair, J. S., Palchaudhuri, R. & Hergenrother, P. J. Chemistry and biology of deoxynyboquinone, a potent inducer of cancer cell death. *J. Am. Chem. Soc.* **132**, 5469-5478, (2010).

6 Parkinson, E. I., Bair, J. S., Cismesia, M. & Hergenrother, P. J. Efficient NQO1 substrates are potent and selective anticancer agents. *ACS Chem Biol* **8**, 2173-2183, (2013).
